# Activation of ERBB4 Pathway Inhibits Pathological Transdifferentiation of Lung Epithelial Progenitors into CD66c^+^ Basal Cells in Severe Lung Injury

**DOI:** 10.1002/advs.202519151

**Published:** 2026-04-07

**Authors:** Kaijun Lin, Xinran Deng, Haonan Wang, Yamei Jiang, Jian Sun, Hailin Ding, Ming Ye, Xiaoting Wang, Yu Wang, Li Yuan, Zhenju Song, Xinhua Lin, Shenfei Sun, Ning Jiang

**Affiliations:** ^1^ State Key Laboratory of Genetic and Development of Complex Phenotypes School of Life Sciences Zhongshan Hospital, Fudan University Shanghai China; ^2^ Greater Bay Area Institute of Precision Medicine Guangzhou China; ^3^ Department of Emergency Medicine Zhongshan Hospital, Fudan University Shanghai China; ^4^ Department of Cardiothoracic Surgery Children's Hospital Fudan University Shanghai China; ^5^ State Key Laboratory of Reproductive Regulation and Breeding of Grassland Livestock Institutes of Biomedical Sciences School of Life Sciences Inner Mongolia University Hohhot China; ^6^ Lead contact

**Keywords:** CD66c^+^ basal cells, distal lung organoids, epithelial plasticity, ERBB4 signaling, idiopathic pulmonary fibrosis (IPF)

## Abstract

Aberrant accumulation of basal cells in the distal lung is a hallmark of impaired epithelial regeneration and is closely associated with fibrotic remodeling; however, their cellular origins and the mechanisms governing their expansion remain unclear. Here, this study establishes human distal lung organoids (DLOs) as a physiologically relevant model to investigate epithelial repair. Single‐cell transcriptomic and functional analyses identify a CD66c^+^ basal cell subset in DLOs that resembles basal cell states enriched in idiopathic pulmonary fibrosis (IPF) lungs and exhibits inflammatory and profibrotic transcriptional programs. The data further demonstrate that secretory cells, alveolar type 2 (AT2) cells, and resident basal cells can each generate CD66c^+^ basal cells, indicating substantial epithelial lineage plasticity. Mechanistically, the GSK3 inhibitor CHIR99021 stabilizes secretory and AT2 identities and prevents their conversion into CD66c^+^ basal cells, accompanied by activation of ERBB4‐MAPK signaling. Moreover, exogenous NRG1 directly restricts this transdifferentiation through ERBB4‐dependent signaling, reinforcing the role of ERBB4 in maintaining epithelial lineage stability. Notably, ERBB4 expression is reduced in IPF tissues, coinciding with expansion of the CD66c^+^ basal cell population. Together, these findings identify ERBB4 signaling as a critical regulator that constrains pathological epithelial remodeling during severe lung injury.

## Graphical abstract

In fibrotic distal lung regions, CD66c^+^ basal cells emerge as a pathological state. Using human distal lung organoids, this study identifies CD66c^+^ basal cells as a pro‐fibrotic state arising through transdifferentiation from secretory, AT2, and basal cells. NRG1‐ERBB4 signaling stabilizes epithelial identity and restrains this convergence, whereas ERBB4 loss in pulmonary fibrosis coincides with CD66c^+^ basal cell expansion.

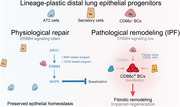

## 1 Introduction

Lung repair following injury depends on the coordinated activation and differentiation of epithelial progenitor populations [1, 2, 3, 4, 5, 6, 7]. When this regenerative program becomes dysregulated, epithelial plasticity may shift toward maladaptive remodeling, ultimately leading to chronic tissue scarring. Such maladaptive repair responses are characteristic of severe lung diseases, including idiopathic pulmonary fibrosis (IPF), acute respiratory distress syndrome (ARDS), and post‐viral sequelae [8, 9, 10, 11, 12, 13, 14, 15, 16]. A prominent pathological feature of these conditions is the ectopic expansion of basal cells (BCs) into distal alveolar regions, where they form atypical epithelial structures associated with impaired regeneration and progressive fibrosis [9, 12, 13, 14, 15, 16].

Recent single‐cell analyses have revealed substantial heterogeneity within BC populations and identified transitional progenitor states that persist in fibrotic niches and correlate with adverse clinical outcomes [10, 17, 18, 19, 20, 21, 22, 23, 24]. Among these, CD66c^+^ BCs—defined by expression of CD66c, the surface protein encoded by *CEACAM6*, and enriched for profibrotic mediators—represent a distinct basal subset expanded in fibrotic lungs and functionally linked to myofibroblast activation and extracellular matrix deposition [17, 25]. Two recent studies independently described CD66c^+^ BCs in IPF lungs, designating them as secretory‐primed basal cells (SPBs) [17] or Cluster B [25], with substantial transcriptional overlap between these populations. Despite these advances, the developmental origins and regulatory mechanisms underlying the emergence of CD66c^+^ BCs remain poorly defined. Notably, ERBB4 signaling has been implicated in lung injury and fibrosis, and activation of the NRG1‐ERBB4 axis exerts anti‐fibrotic and cytoprotective effects in injured mouse lungs [26]. However, whether ERBB4 contributes to epithelial lineage stability or regulates the formation of CD66c^+^ BCs remains unknown. Multiple epithelial sources—including preexisting airway BCs, secretory cells, and alveolar type 2 (AT2) cells—have been proposed as contributors to ectopic BC emergence in diverse experimental models [15, 27, 28, 29, 30]. However, species‐specific differences and the complexity of the in vivo microenvironment pose challenges to mechanistic dissection [4, 5, 31]. In particular, observations from human IPF and ARDS tissues suggest that AT2 cells can contribute to aberrant basalization [8, 32, 33], underscoring the need for human‐relevant models to resolve these processes [34].

Organoid technology provides a reductionist system to model epithelial dynamics under defined conditions. Human distal lung organoids (DLOs) preserve distal epithelial diversity and lineage potential, enabling analysis of epithelial cell‐intrinsic fate decisions in a human‐relevant context that is difficult to achieve in vivo. Here, we used DLOs to recapitulate the emergence of CD66c^+^ BCs and to delineate their lineage origins. Through integrated single‐cell transcriptomic analyses, functional assays, and pharmacological perturbations, we demonstrate that CD66c^+^ BCs arise from multiple epithelial sources and exhibit profibrotic features. Furthermore, we identify ERBB4 signaling as a critical pathway that stabilizes secretory and AT2 cell identities by restricting pathological BC conversion. Collectively, these findings provide insight into epithelial plasticity during severe lung injury and highlight ERBB4 as a potential therapeutic target to limit maladaptive remodeling.

## 2 Results

### 2.1 Expansion of Basal Cells in Organoids Derived from Human Distal Lung Epithelial Cells

**FIGURE 1:**
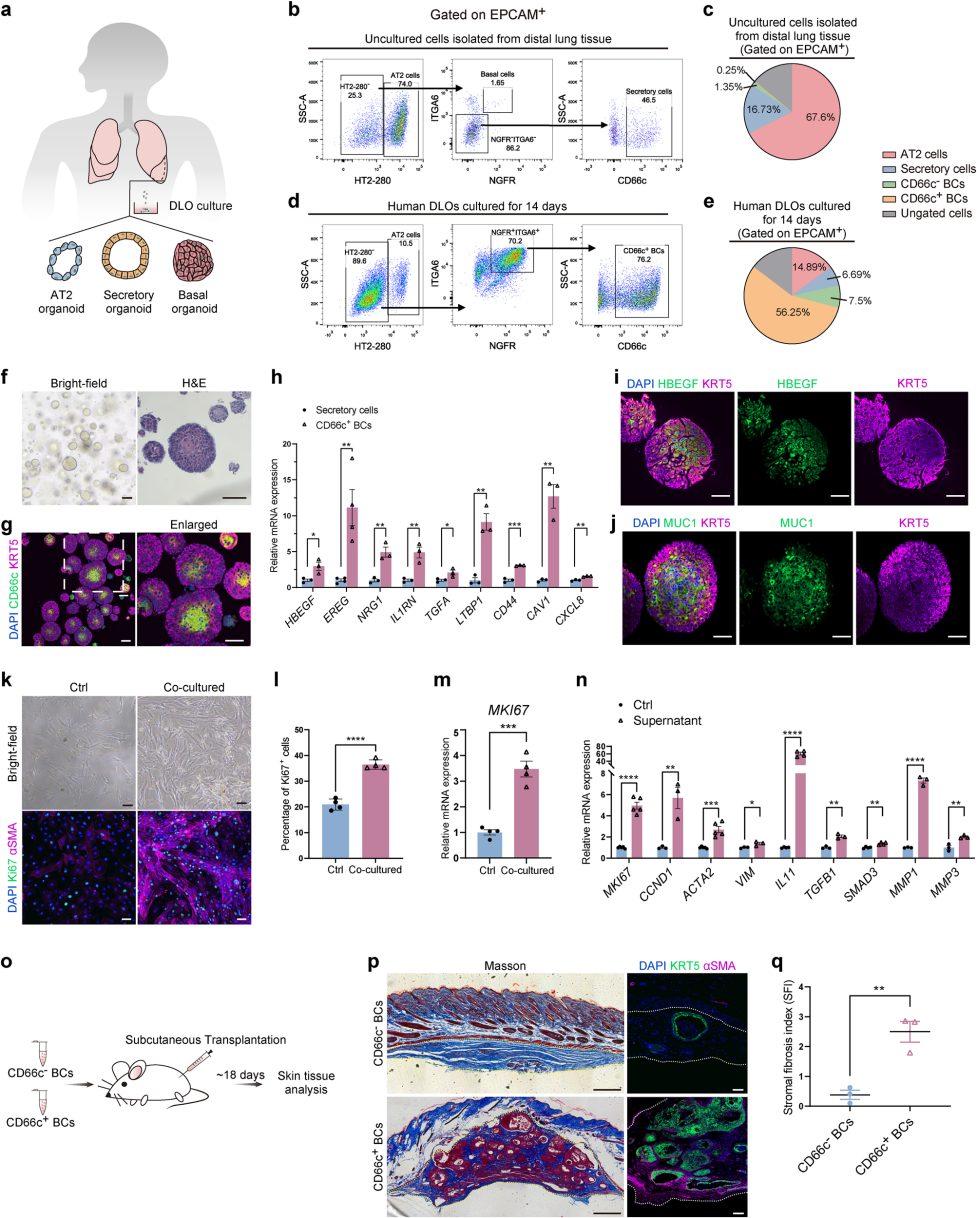
Emergence of CD66c^+^ basal cells (BCs) in distal lung organoids and their pro‐fibrotic properties in vitro and in vivo. (a) Schematic diagram of human distal lung organoid (DLO) cultures generating alveolar type 2 (AT2) organoids, secretory cell organoids (SCOs), and basal cell organoids (BCOs). (b–e) Flow cytometry analysis of EPCAM^+^ epithelial cells from uncultured distal lung tissue (b, c) and day 14 DLOs (d, e), showing proportions of AT2 cells, secretory cells, CD66c^−^ BCs, and CD66c^+^ BCs (*n* = 3). (f) Morphology of DLOs. Bright‐field (left) and H&E staining (right) show representative BCO structures. Scale bars, 50 µm. (g) Immunofluorescence (IF) staining of BCOs showing CD66c (green) and KRT5 (magenta) double‐positive cells. Boxed region is enlarged on the right. Scale bars, 50 µm. (h) RT‐qPCR analysis of ERBB ligands and fibrosis‐related genes in FACS‐sorted CD66c^+^ BCs compared with secretory cells (*n* = 3). (i, j) IF staining of BCOs showing HBEGF (i) or MUC1 (j) co‐expressed with KRT5. Nuclei counterstained with DAPI. Scale bars, 50 µm. (k) Representative bright‐field images and immunofluorescence staining for αSMA (magenta) and Ki67 (green) of MRC‐5 fibroblasts cultured alone or co‐cultured with CD66c^+^ BCs. (l) Percentage of Ki67^+^ MRC‐5 fibroblasts cultured alone or co‐cultured with CD66c^+^ BCs (*n* = 4). (m) Relative mRNA expression of *MKI67* of MRC‐5 fibroblasts cultured alone or co‐cultured with CD66c^+^ BCs. Data are shown as mean ± SEM (*n* = 4). (n) RT‐qPCR of MRC‐5 fibroblasts treated with conditioned medium from CD66c^+^ BC organoids, showing upregulation of fibroblast activation‐associated genes (*n* = 3–5). (o–q) In vivo fibrosis assay. (o) Experimental scheme of subcutaneous transplantation of CD66c^−^ or CD66c^+^ BCs into NSG mice. (p) Representative Masson's trichrome and IF staining showing collagen deposition and αSMA^+^ myofibroblasts in CD66c^+^ BC grafts. Scale bars, 250 µm (Masson), 100 µm (IF). (q) Quantification of the stromal fibrosis index (SFI). Data represent mean ± SEM (*n* = 3). Data are shown as mean ± SEM. Statistical significance was determined by unpaired two‐tailed Student's *t*‐test. *n* represents independent biological replicates. *p < 0.05, **p < 0.01, ***p < 0.001, ****p < 0.0001.

Human DLOs provide a robust system for studying epithelial progenitor dynamics [35, 36]. In established DLO cultures, we observed three morphologically distinct organoid subtypes: AT2 organoids (AT2‐Org; spherical structures approximately 50 µm in diameter), dense spherical organoids (hereafter termed basal cell organoids, BCOs; compact, non‐luminal aggregates expressing KRT5), and secretory cell organoids (SCOs; organized luminal structures) (Figure 1a, Figure S1a). These distinct morphologies enabled classification for quantitative analysis and subsequent functional characterization.

To compare the epithelial composition of uncultured distal lung tissue with that of derived organoids, we performed flow cytometry using established lineage markers: HT2‐280 (AT2), NGFR and ITGA6 (basal), and CD66c (secretory) (Figure S2). In uncultured distal lung epithelium (EPCAM^+^ gated), AT2 cells predominated (∼67.6%), followed by secretory cells (∼16.7%) and rare BCs (∼1.6%). Within the BC compartment, CD66c^+^ cells were uncommon (∼0.25% of EPCAM^+^ epithelial cells), whereas CD66c^−^ BCs accounted for ∼1.35% (Figure 1b,c; *n* = 3 donors). Immunofluorescence staining of distal lung tissue confirmed that KRT5^+^ BCs were largely confined to airway regions and comprised only a small fraction of epithelial cells (Figure S1b). After 14 days in culture, DLOs exhibited marked cellular remodeling. BCs expanded to ∼63.8% of EPCAM^+^ epithelial cells, with a substantial proportion acquiring CD66c expression—representing a pronounced increase compared with uncultured tissue (Figure 1d,e). Correspondingly, BCOs became the dominant organoid type (Figure 1f). Immunostaining confirmed co‐expression of CD66c and KRT5 within BCOs (Figure 1g). This phenotype resembles the CD66c^+^ BC population enriched in IPF lungs but rare in healthy lungs [17, 25]. RT‐qPCR further demonstrated significant upregulation of basal markers (*KRT5*, *TP63*) in DLOs relative to uncultured tissue (Figure S1d,e).

### 2.2 CD66c^+^ Basal Cells Promote Fibroblast Activation In Vitro and Induce Fibrotic Remodeling In Vivo

We next investigated whether CD66c^+^ BCs in DLOs possess functional properties relevant to fibrotic remodeling. Compared with secretory cells isolated by fluorescence‐activated cell sorting (FACS), CD66c^+^ BCs exhibited markedly increased expression of fibrosis‐associated factors, including ERBB family ligands (e.g., *HBEGF*, *EREG*, *NRG1*, and *TGFA*), fibrosis‐related regulators (e.g., *LTBP1*, *CD44*, and *CAV1*), and inflammatory mediators (e.g., *IL1RN* and *CXCL8*) (Figure 1h). This transcriptional profile parallels that reported for IPF‐associated CD66c^+^ BCs [25]. Immunofluorescence staining confirmed strong protein expression of profibrotic factors such as heparin‐binding EGF‐like growth factor (HB‐EGF) in DLO cultures (Figure 1i). Moreover, markers characteristic of SPBs in IPF lungs [17], including MUC1 and KRT16, were highly expressed in DLOs (Figure 1j, Figure S1c).

To assess the impact of CD66c^+^ BCs on stromal activation, we co‐cultured CD66c^+^ BCs with MRC‐5 fetal lung fibroblasts. Fibroblasts co‐cultured with CD66c^+^ BCs showed increased proliferative activity, as indicated by a higher proportion of Ki67^+^ cells quantified by immunofluorescence compared with controls (Figure 1k,l). αSMA expression was also elevated in co‐cultured fibroblasts, consistent with enhanced myofibroblast activation (Figure 1k). In parallel, *MKI67* transcript levels were increased in fibroblasts following co‐culture with CD66c^+^ BCs (Figure 1m). Conditioned medium derived from CD66c^+^ BC organoids recapitulated these effects, resulting in increased Ki67 positivity and αSMA expression (Figure S3a,b). At the transcriptional level, CD66c^+^ BC‐conditioned medium induced upregulation of proliferation‐associated genes (*MKI67*, *CCND1*), extracellular matrix regulators (*MMP1*, *MMP3*), myofibroblast markers (*ACTA2*, *VIM*), and fibrogenic mediators (*IL11*, *TGFB1*, *SMAD3*) (Figure 1n). Together, these data indicate that CD66c^+^ BCs promote fibroblast proliferation and activation in vitro.

We next evaluated whether CD66c^+^ BCs induce fibrotic remodeling in vivo. FACS‐sorted CD66c^+^ BCs were transplanted subcutaneously into NSG mice (Figure 1o). By day 18 post‐injection, Masson's trichrome staining revealed increased collagen deposition at CD66c^+^ BC engraftment sites, whereas transplants of CD66c^−^ BCs elicited minimal fibrotic responses (Figure 1p). Immunofluorescence analysis further demonstrated dense accumulation of αSMA^+^ myofibroblasts surrounding CD66c^+^KRT5^+^ BC‐derived organoids, a feature largely absent in CD66c^−^ BC transplants (Figure 1p, Figure S3c,d). Quantification of the stromal fibrosis index (SFI; αSMA^+^ area/total stromal area) revealed a 6.6‐fold increase in CD66c^+^ BC‐injected mice compared with controls (2.50% ± 0.31% vs. 0.38% ± 0.15%; *p* < 0.01; Figure 1q; *n* = 3 mice). Collectively, these findings demonstrate that CD66c^+^ BCs promote fibroblast activation and fibrotic remodeling both in vitro and in vivo.

### 2.3 DLO‐Derived Basal Cells Resemble IPF‐Associated Metaplastic States

**FIGURE 2:**
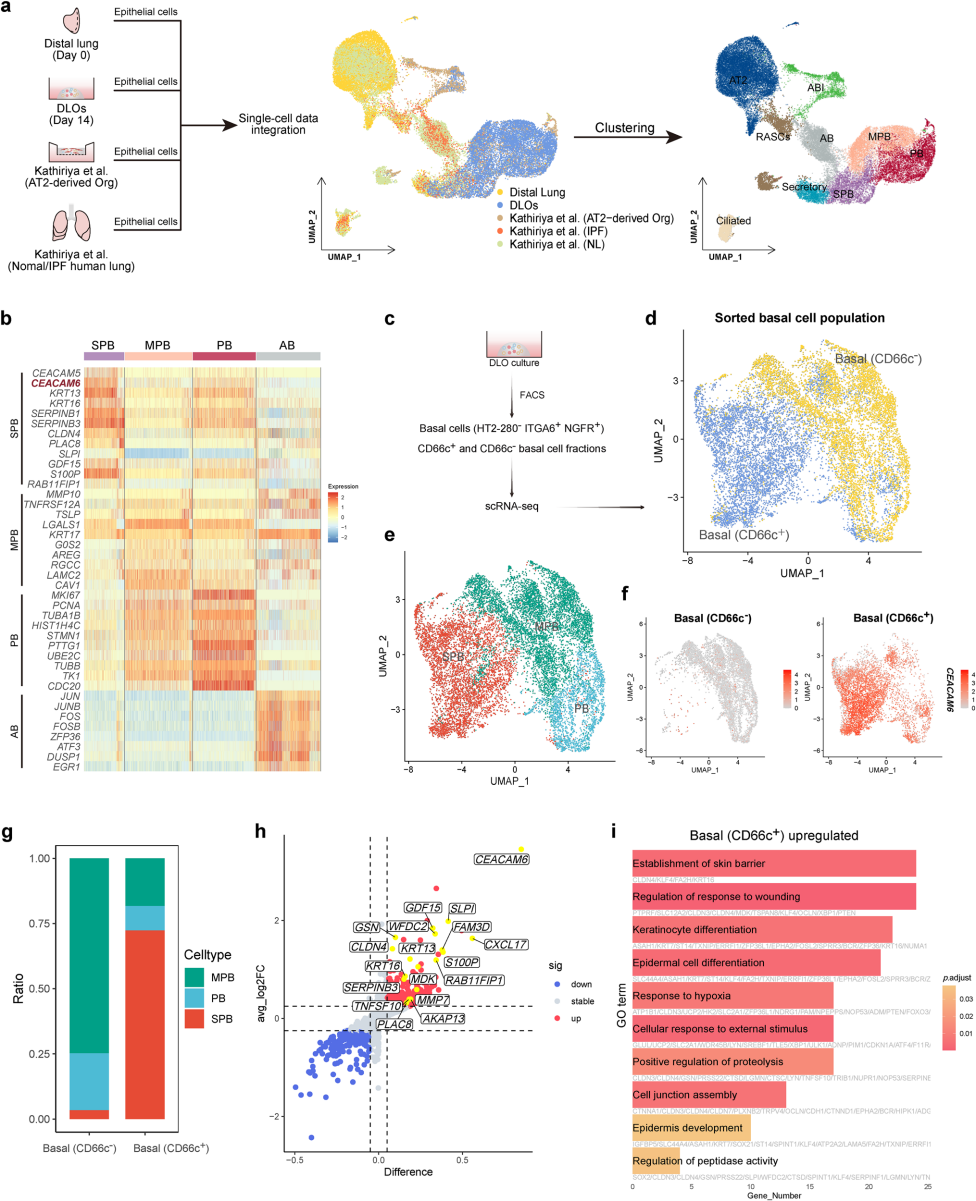
Integrated single‐cell RNA‐seq analysis delineates basal cell heterogeneity conserved between distal lung organoids (DLOs) and idiopathic pulmonary fibrosis (IPF) Lungs. (a) EPCAM^+^ epithelial cells isolated from uncultured distal lung tissue (Day 0) and day‐14 distal lung organoids (DLOs) were integrated with published single‐cell RNA‐seq datasets from AT2‐derived organoids and normal or IPF human lungs (Kathiriya et al.). Uniform Manifold Approximation and Projection (UMAP) embeddings are shown with cells colored by sample origin (middle) and by epithelial cell type annotation (right), demonstrating alignment of major epithelial populations across systems. (b) Heatmap summarizing representative transcriptional signatures across basal cell (BC) subtypes identified in the integrated dataset. Secretory‐primed basal cells (SPBs) are enriched in the CD66c^+^ BC fraction, whereas multipotent basal cells (MPBs), proliferating basal cells (PBs), and activated basal cells (ABs) are predominantly associated with the CD66c^−^ fraction. (c) Experimental scheme and UMAP visualization of basal cells isolated from DLO cultures. BCs were FACS‐sorted based on the phenotype HT2‐280^−^ ITGA6^+^ NGFR^+^ and further separated into CD66c^+^ and CD66c^−^ fractions prior to single‐cell RNA sequencing. The UMAP shows the transcriptional landscape of the sorted BC populations. (d) UMAP embedding of sorted BCs colored by transcriptionally defined subtypes, including SPB, MPB, and PB populations, revealing structured heterogeneity within the basal compartment. (e) UMAP visualization of sorted BCs split by CD66c status and projected onto a shared embedding. Cells are colored by *CEACAM6* expression, highlighting enrichment of *CEACAM6* within the CD66c^+^ BC population. (f) Stacked bar plot showing the relative proportions of SPB, PB, and MPB subtypes within FACS‐sorted CD66c^−^ and CD66c^+^ BC fractions, based on single‐cell transcriptomic classification. (g) Volcano plot depicting differentially expressed genes between CD66c^+^ and CD66c^−^ BCs, plotted by average log_2_ fold change and statistical significance. (h) Gene Ontology enrichment analysis of genes upregulated in FACS‐sorted CD66c^+^ BCs (adjusted *p* < 0.05).

To evaluate the similarity between DLO‐derived BCs and IPF‐associated metaplastic states, we integrated scRNA‐seq data from FACS‐sorted EPCAM^+^ epithelial cells derived from uncultured distal lung tissue and DLOs. These datasets were combined with published epithelial profiles [8], including AT2‐derived organoids, normal lungs, and IPF tissues (Figure 2a, Figure S4c,e,f). Cell identities were assigned based on canonical marker expression, resolving ciliated, secretory, basal, AT2, respiratory airway secretory cells (RASCs), and alveolar‐basal intermediate (ABI) populations (Figure S4a,f). Integrated UMAP analysis identified four transcriptionally conserved BC subsets shared across datasets, with variable representation across samples: SPBs (*CEACAM6* ^+^ *KRT5* ^+^), multipotent basal cells (MPBs), activated basal cells (ABs), and proliferating basal cells (PBs) (Figures S4d and S5a). ABs were readily detected in IPF lungs but were largely absent from organoid samples (Figure 2b, Figure S4b,f). Functional enrichment analysis comparing SPBs and ABs showed that SPBs were enriched for epithelial‐intrinsic programs related to keratinization, cell–cell junction organization, and protease‐related remodeling, whereas ABs preferentially exhibited inflammatory and cytokine‐associated signatures (Figure S5b). Together, these analyses indicate that SPBs are linked to epithelial remodeling programs, while ABs represent inflammation‐responsive activation states. Consistently, SPBs were enriched in both DLOs and IPF lungs relative to normal controls (Figure S4b). Moreover, SPBs were derived from DLOs localized adjacent to their IPF counterparts in UMAP space (Figure S4e,f), indicating strong transcriptional concordance.

To define CD66c‐associated BC states independently of cluster‐based inference, we isolated CD66c^+^ and CD66c^−^ BCs from DLO cultures by FACS. HT2‐280^−^ NGFR^+^ ITGA6^+^ epithelial cells were stratified according to surface CD66c expression and subjected to separate scRNA‐seq analysis (Figure 2c,d). Integrated analysis confirmed comparable expression of canonical BC markers in both fractions, validating their basal identity (Figure S6b). Reclustering resolved three principal BC states—SPBs, MPBs, and PBs (Figure 2e, Figure S6a,c). FACS‐sorted CD66c^+^ BCs were strongly enriched for the SPB state, whereas CD66c^−^ BCs were depleted of SPBs and predominantly comprised MPBs and PBs (Figure 2f,g, Figure S6c), demonstrating selective enrichment of SPBs through CD66c‐based sorting. Differential expression analysis showed that CD66c^+^ BCs upregulated a transcriptional program consistent with secretory priming and epithelial stress adaptation. This program included *CEACAM6* and genes associated with metaplastic or secretory‐primed basal states, such as *KRT13*, *KRT16*, *CLDN4*, *WFDC2*, *SLPI*, and *PLAC8*, as well as injury‐ and inflammation‐related mediators (e.g., *CXCL17*, *GDF15*, *MDK*) and extracellular matrix remodeling factors including *MMP7* (Figure 2h). Gene ontology analysis further indicated enrichment of epithelial‐intrinsic processes among genes upregulated in CD66c^+^ BCs, including keratinocyte and epidermal differentiation, epithelial barrier establishment, protease‐associated tissue remodeling, cell junction assembly, and responses to wounding and cellular stress (Figure 2i). In contrast, genes upregulated in CD66c^−^ BCs were primarily associated with cell cycle progression and mitotic regulation, including nuclear division and microtubule cytoskeleton organization involved in mitosis (Figure S6d). Enrichment was also observed for integrin signaling, cell–substrate adhesion, and cytoskeletal organization pathways, consistent with the predominance of MPB and PB states in the CD66c^−^ basal compartment. Collectively, these data define functionally distinct basal compartments in DLOs and demonstrate that CD66c^+^ BCs adopt a secretory‐primed, stress‐responsive program resembling metaplastic basal states observed in fibrotic human lungs.

We next integrated DLO‐derived BC transcriptomes with published in vitro IPF BC libraries [25] (Figure S7a). Two conserved transcriptional clusters (A and B) were identified across DLOs and IPF basal clones (Figure S7c). The proportion of Cluster B cells in DLOs (30.8%) was comparable to that observed in IPF basal clones (IPF1: 21.3%; IPF2: 30.3%) (Figure S7b). Cluster B represented a pro‐fibrotic subtype characterized by *CEACAM6* expression together with induction of mediators such as *CXCL17*, *IL1RN*, and *CLDN4*, alongside canonical SPB markers including *CEACAM5*, *KRT16*, *PLAC8*, and *SLPI* (Figure S7e,f). These findings further demonstrate that DLO‐derived BCs recapitulate key transcriptional features of IPF‐associated BC subpopulations.

### 2.4 Transdifferentiation Potential of Different Distal Lung Progenitors into CD66c^+^ Basal Cells

**FIGURE 3:**
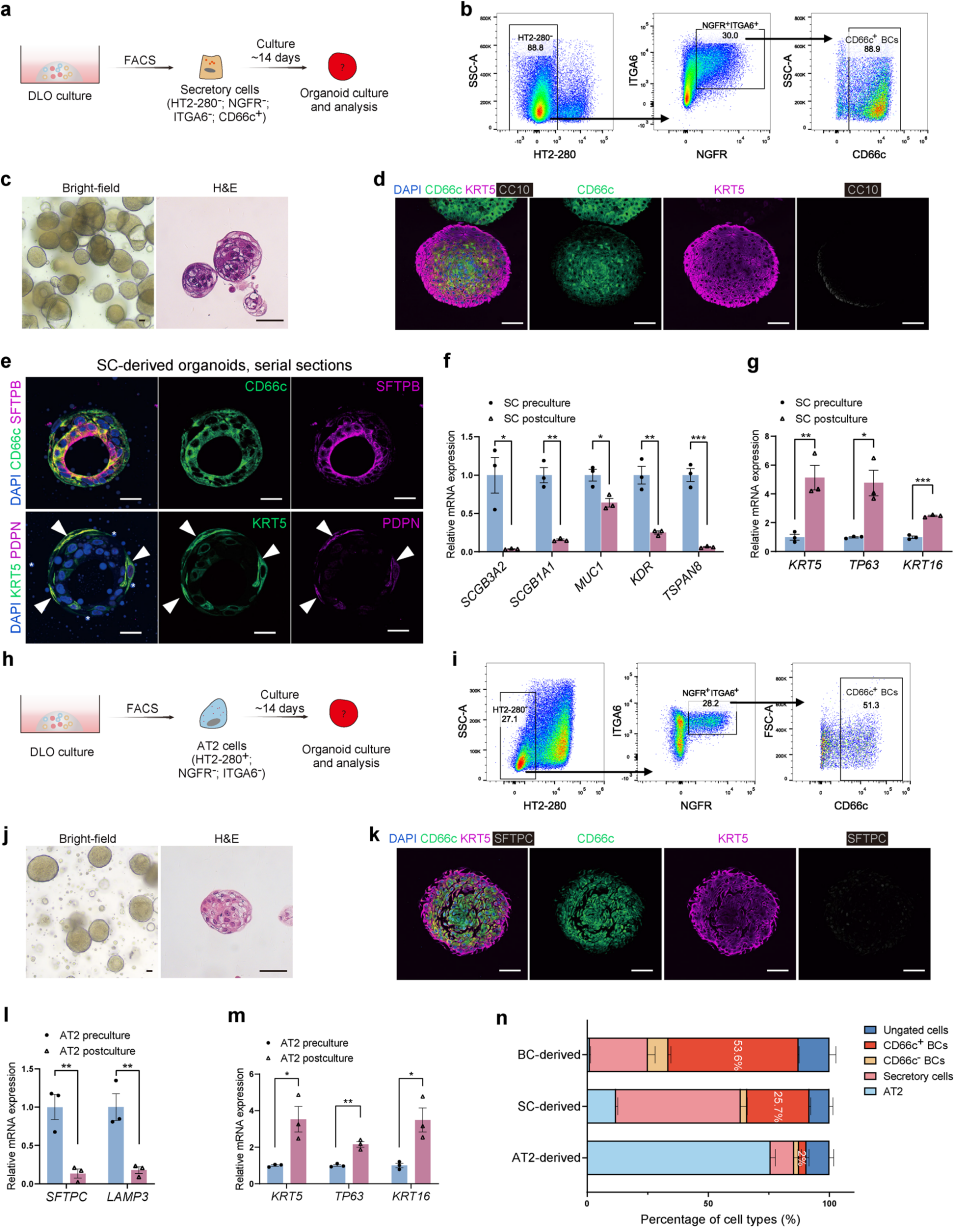
Distal lung progenitors generate CD66c^+^ basal cells (BCs) in organoid cultures. (a) Experimental workflow: FACS isolation of secretory cells (HT2‐280^−^ NGFR^−^ ITGA6^−^ CD66c^+^) from DLOs, followed by approximately 14 days of three‐dimensional culture. (b) Flow cytometry analysis of epithelial populations in secretory cell‐derived organoids at day 14, highlighting the proportion of CD66c^+^ basal cells. (c) Bright‐field (left) and H&E staining (right) of secretory‐derived organoids. Scale bar, 50 µm. (d) Immunofluorescence showing DAPI (blue), CD66c (green), KRT5 (magenta), and CC10 (gray). Scale bar, 50 µm. (e) Serial sections of secretory‐derived organoids showing CD66c with SFTPB (top) and KRT5 with PDPN (bottom). Arrowheads indicate squamous basal cells; asterisks denote nonspecific staining. Scale bars, 50 µm. (f, g) RT‐qPCR analysis of secretory cells before and after culture, showing loss of secretory markers (f) and induction of basal markers (*KRT5*, *TP63*) and the SPB‐associated marker *KRT16* (g) (*n* = 3). (h) Experimental workflow: FACS isolation of AT2 cells (HT2‐280^+^ NGFR^−^ ITGA6^−^) from DLOs, followed by approximately 14 days of culture. (i) Flow cytometry of AT2‐derived organoids showing the emergence of CD66c^+^ BCs. (j) Bright‐field (left) and H&E staining (right) of AT2‐derived organoids, showing compact spheroid structures. Scale bar, 50 µm. (k) Immunofluorescence showing DAPI (blue), CD66c (green), KRT5 (magenta), and the AT2 marker SFTPC (gray). Scale bar, 50 µm. (l, m) RT‐qPCR analysis of AT2 cells before and after culture, showing decreased expression of AT2 markers (l) and increased expression of basal markers and the SPB‐associated marker *KRT16* (m) (*n* = 3). (n) Quantification of cell type composition in BC‐, SC‐, and AT2‐derived organoids by flow cytometry. Populations include ungated cells, CD66c^+^ BCs, CD66c^−^ BCs, secretory cells, and AT2 cells, with CD66c^+^ BC proportions highlighted (*n* = 3). CD66c^+^ BC proportions differed significantly across groups (one‐way ANOVA with Tukey's multiple comparisons test). Data are shown as mean ± SEM. Statistical significance was determined by unpaired two‐tailed Student's *t*‐test for comparisons between two groups and by one‐way ANOVA with Tukey's multiple comparisons test for multiple group comparisons. *n* represents independent biological replicates. *p < 0.05, **p < 0.01, ***p < 0.001. [Correction added on 21 April 2026 after first online publication: Figure 3m has been updated.]

To elucidate the cellular sources of CD66c^+^ BCs, we hypothesized that these cells arise either through transdifferentiation of non‐basal epithelial progenitors or through conversion of pre‐existing BCs. To test this, we FACS‐sorted three epithelial populations from DLOs: secretory cells (CD66c^+^ NGFR^−^ ITGA6^−^ HT2‐280^−^), AT2 cells (HT2‐280^+^ NGFR^−^ ITGA6^−^), and CD66c^−^ BCs (CD66c^−^ NGFR^+^ ITGA6^+^ HT2‐280^−^). Each population was cultured independently in 3D to assess its capacity to generate CD66c^+^ BCs (Figure 3a,h; Figure S10a,g). The purity of sorted populations was confirmed by scRNA‐seq and cytospin analyses (Figure S8b–i, Figure S9).

Under organoid‐forming conditions, secretory cells predominantly generated compact spheroid structures with minimal lumen formation (Figure 3c). Immunofluorescence and flow cytometry demonstrated enrichment of BCs, the majority of which expressed CD66c (Figure 3b,d). Serial sectioning revealed progressive architectural remodeling, with secretory‐derived cells transitioning into BCs and forming organoids composed of an inner polarized epithelium surrounded by a squamous‐like outer layer (Figure 3e, Figure S10f). RT‐qPCR analysis of secretory cells before and after culture showed downregulation of secretory markers and induction of BC markers (*KRT5*, *TP63*), along with the SPB‐associated marker *KRT16* (Figure 3f,g).

AT2 cells also generated organoids containing CD66c^+^ BCs after 14 days in culture (Figure 3h). Bright‐field microscopy and hematoxylin and eosin (H&E) staining indicated that AT2‐derived organoids were predominantly cystic, although basal‐like structures were also observed (Figure 3j,k). Flow cytometry detected CD66c^+^ BCs at approximately 2% of total epithelial cells (Figure 3i,n). Importantly, CD66c^+^ BCs represent only a subset of the basal compartment and do not account for all basal‐like cells arising under these conditions. In addition to the canonical gated epithelial subsets, a minor EPCAM^+^ HT2‐280^−^ ITGA6^+^ NGFR^−^ epithelial fraction was consistently detected (Figure 3n, Figure S8j). Cytospin immunofluorescence further showed that approximately 40% of cells within this ungated fraction expressed KRT5, indicating basal features (Figure S8k,l). RT‐qPCR analysis of AT2‐derived organoids corroborated this trend, demonstrating loss of AT2 markers and induction of basal and SPB‐associated genes, including *KRT16* (Figure 3l,m). In parallel, CD66c^−^ BCs efficiently converted to form densely packed, solid organoids (Figure S10a,b), with approximately 53.6% of cells acquiring CD66c expression by day 14 (Figure 3n, Figure S10d). Immunostaining confirmed co‐expression of CD66c and KRT5 (Figure S10e), and RT‐qPCR demonstrated upregulation of SPB‐associated genes, including *CEACAM6*, *KRT16*, and *KRT13* (Figure S10c).

### 2.5 Progenitor‐Specific Trajectories Converge toward a Conserved CD66c^+^ Basal Cell Transcriptional Program

**FIGURE 4:**
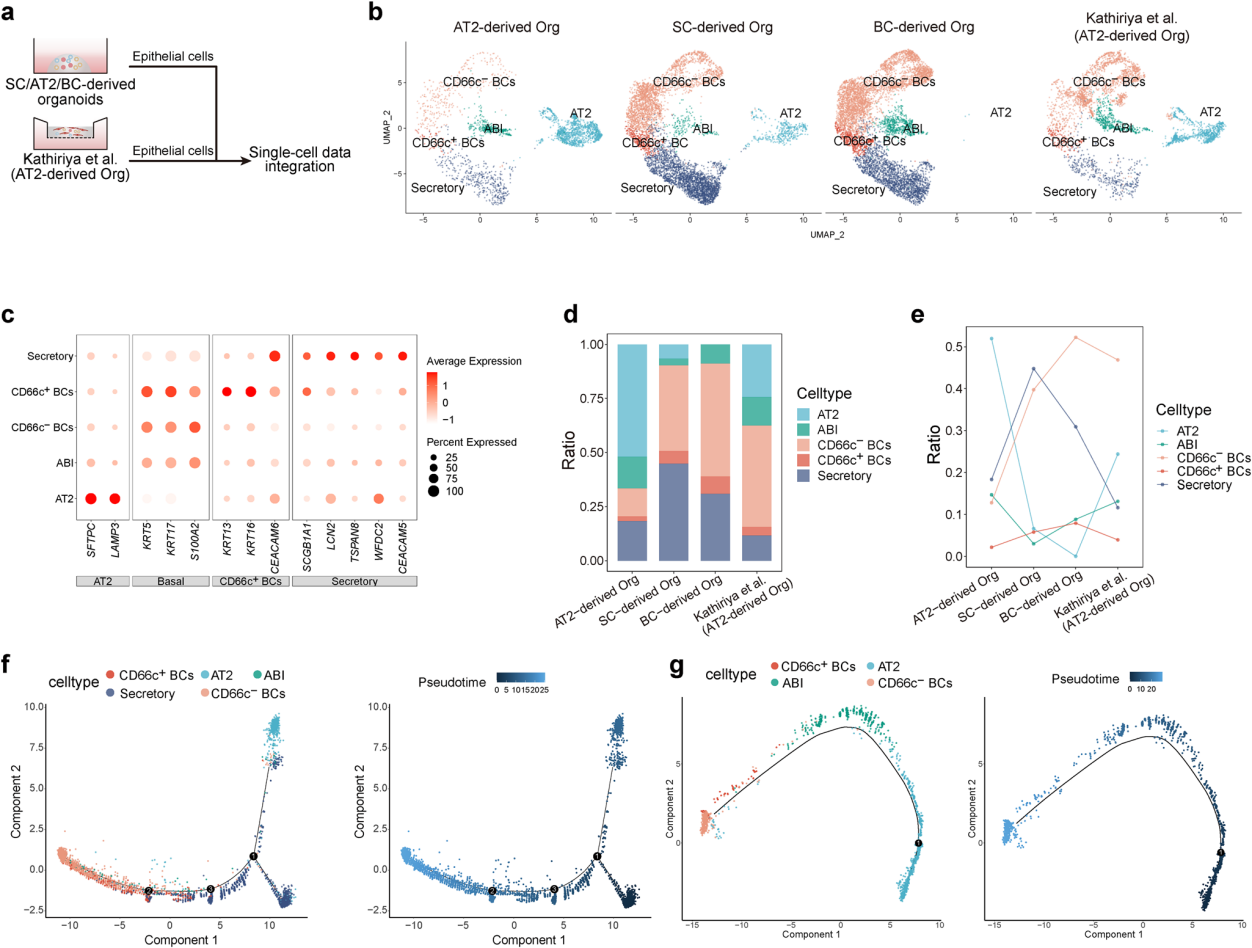
Single‐cell transcriptomic analysis of lineage plasticity in secretory and AT2‐derived organoids. (a) Schematic diagram of the experimental and computational workflow. Organoids derived from AT2 cells, secretory cells (SCs), and CD66c^−^ basal cells (BCs) were profiled by scRNA‐seq and integrated with a published AT2‐derived organoid reference dataset (Kathiriya et al.). (b) UMAP projection of the integrated data showing five epithelial populations: AT2 cells, alveolar–basal intermediates (ABIs), secretory cells, CD66c^−^ BCs, and CD66c^+^ BCs. (c) Dot plot of representative markers validating cluster identities. CD66c^+^ BCs selectively expressed *KRT13*/*KRT16*, whereas ABIs displayed transitional profiles between AT2 and basal states. (d, e) Quantification of epithelial composition across organoids derived from distinct progenitors and the reference dataset. (d) Stacked bar plot showing the relative proportions of epithelial subtypes. (e) Line plot illustrating lineage contributions across progenitor‐derived organoids, highlighting the enrichment of CD66c^+^ BCs in SC‐ and BC‐derived organoids. (f) Pseudotime trajectory of SC‐derived organoids reconstructed with Monocle2, showing bifurcation toward basal and AT2 cell fates. (g) Pseudotime analysis of AT2‐derived organoids (with secretory cells excluded), revealing a continuous trajectory from AT2 cells through ABIs toward CD66c^+^ and CD66c^−^ BC states.

To investigate cellular heterogeneity and transdifferentiation dynamics, we performed scRNA‐seq on organoids derived from AT2 cells, secretory cells, and CD66c^−^ BCs. These datasets were integrated with a published AT2‐derived organoid dataset [8] to refine cell identity definition and improve lineage resolution (Figure 4a). UMAP projection resolved five major transcriptional clusters: CD66c^+^ BCs, CD66c^−^ BCs, secretory cells, AT2 cells, and ABIs (Figure 4b,c). CD66c^+^ BCs expressed canonical SPB‐associated genes, including *KRT13*, *KRT16*, *IL1RN*, and *SERPINB*1/3/5 [17] (Figure S11c). Lineage quantification indicated that all three progenitor populations contributed to the CD66c^+^ BC pool, albeit with distinct efficiencies. Secretory‐derived organoids generated a substantial basal compartment, including a CD66c^+^ fraction (Figure 4d,e), whereas CD66c^−^ BC‐derived organoids produced the highest proportion of CD66c^+^ BCs. AT2‐derived organoids also gave rise to CD66c^+^ BCs, although at lower frequency under these conditions (Figure 4d,e). Trajectory inference suggested that CD66c^−^ BCs contributed to CD66c^+^ BC formation along a largely unidirectional path, progressing primarily toward secretory fates and, to a lesser extent, toward AT2 fates (Figure S11a). In contrast, secretory‐derived organoids exhibited bifurcated trajectories: one transitioning from secretory cells to CD66c^+^ and CD66c^−^ BCs, and another maintaining an alveolar‐directed differentiation route (Figure 4f). For AT2‐derived organoids, the initial trajectory inferred across all epithelial subsets showed an atypical topology in which secretory and BC populations appeared upstream of AT2 cells (Figure S11b). This configuration suggested that secretory cell contamination may have confounded lineage ordering. To address this, we reconstructed pseudotime after excluding secretory cells. The refined trajectory demonstrated a linear progression from AT2 cells through ABIs to CD66c^+^ and CD66c^−^ BCs (Figure 4g), supporting a model in which AT2 cells transdifferentiate into basal fates via an intermediate ABI state.

To determine whether CD66c^+^ BCs derived from different progenitors share a common transcriptional identity, we performed bulk RNA‐seq on FACS‐purified CD66c^+^ and CD66c^−^ BCs from AT2‐, secretory‐, and BC‐derived organoids. Principal component analysis (PCA) showed that CD66c^+^ BCs from all three origins clustered closely in principal component space (PC1: 67.7%; PC2: 11.3%), indicating a conserved transcriptional program (Figure S12b). In contrast, CD66c^−^ BCs exhibited greater heterogeneity across samples. Hierarchical clustering further confirmed that CD66c^+^ BCs grouped together irrespective of progenitor source (Figure S12c). Collectively, these findings indicate that CD66c^+^ BCs converge toward a shared transcriptional state independent of progenitor origin.

### 2.6 CHIR99021 Suppresses Transdifferentiation of Secretory and AT2 Cells Into CD66c^+^ Basal Cells

**FIGURE 5:**
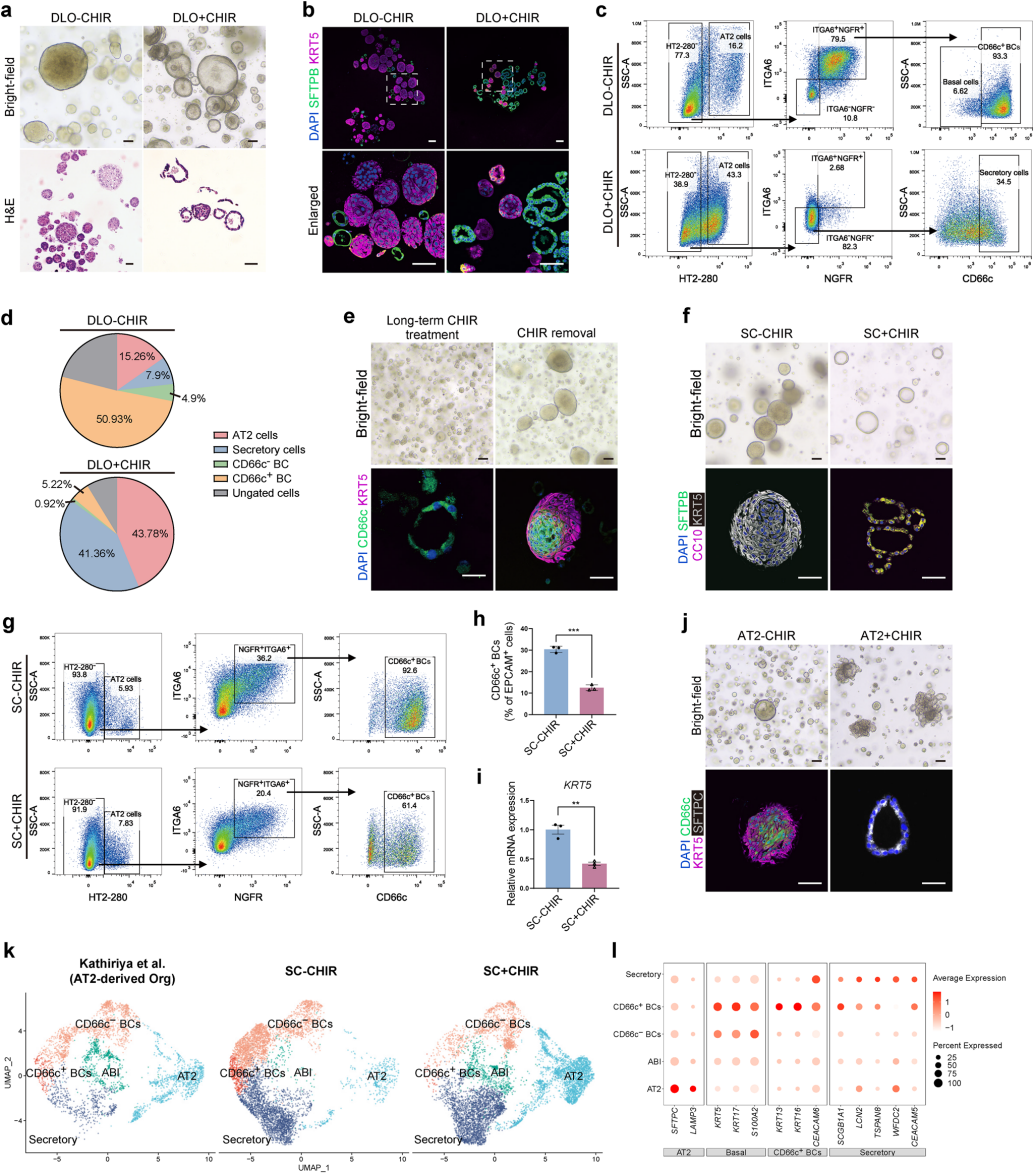
CHIR99021 treatment preserves secretory and AT2 cell identities and suppresses their transdifferentiation Into CD66c^+^ basal cells. (a) Bright‐field (top) and H&E (bottom) images of distal lung organoids (DLOs) cultured without (DLO–CHIR) or with CHIR99021 (DLO+CHIR). CHIR promoted a lumenized organoid morphology and reduced the formation of solid spheroids. Scale bars, 50 µm. (b) Immunofluorescence staining of DLOs showing nuclei (DAPI, blue), SFTPB (green), and KRT5 (magenta). Enlarged views of boxed regions are shown below. Scale bars, 50 µm. (c) Flow cytometry of EPCAM^+^ epithelial cells from DLO–CHIR and DLO+CHIR showing reduced proportions of CD66c^+^ basal cells (BCs) upon CHIR treatment. (d) Pie charts summarizing epithelial composition in −CHIR versus +CHIR DLOs (*n* = 3). (e) Bright‐field (top) and immunofluorescence (bottom) of DLOs after long‐term CHIR exposure (>60 days) versus CHIR withdrawal, showing reappearance of CD66c^+^ BCs upon CHIR removal. Scale bars, 50 µm. (f) Bright‐field (top) and immunofluorescence (bottom) of secretory‐derived organoids cultured 14 days without (SC–CHIR) or with CHIR (SC+CHIR). SC–CHIR organoids formed solid spheroids enriched in KRT5^+^ BCs, whereas SC+CHIR organoids formed hollow structures enriched for SFTPB^+^ and CC10^+^ secretory cells. Scale bars, 50 µm. (g) Representative FACS plots of EPCAM^+^ cells from SC‐derived organoids (−CHIR vs. +CHIR). (h) Quantification of CD66c^+^ BCs within EPCAM^+^ cells showing a significant reduction in the CHIR‐treated group (*n* = 3). (i) RT‐qPCR analysis showing reduced *KRT5* expression in SC+CHIR organoids compared with SC–CHIR controls (*n* = 3). (j) Bright‐field (top) and immunofluorescence (bottom) of AT2‐derived organoids cultured 14 days without or with CHIR. −CHIR organoids formed dense spheroids expressing CD66c (green) and KRT5 (magenta), whereas +CHIR organoids retained AT2 identity, marked by SFTPC (gray). Scale bars, 50 µm. (k) UMAP of scRNA‐seq data from SC‐derived organoids (−CHIR vs. +CHIR) integrated with the published AT2‐derived dataset (Kathiriya et al.) to assess CHIR‐induced shifts in epithelial cell states, showing AT2, alveolar–basal intermediates (ABIs), secretory cells, CD66c^−^ BCs, and CD66c^+^ BCs. (l) Dot plot of representative marker genes for each population. Dot size indicates the percentage of positive cells; color intensity reflects average expression levels. Data are shown as mean ± SEM. Statistical significance was determined by unpaired two‐tailed Student's *t*‐test. *n* represents independent biological replicates. **p < 0.01, ***p < 0.001.

To investigate mechanisms regulating epithelial plasticity, we treated DLOs with CHIR99021 (CHIR), a GSK3β inhibitor reported to stabilize alveolar identity [37, 38, 39, 40]. In untreated cultures, DLOs predominantly generated dense BCOs, whereas CHIR treatment shifted organoid morphology toward hollow, lumenized structures (Figure 5a). Immunofluorescence staining supported this morphological change, showing reduced KRT5^+^ BCs and an increased abundance of SFTPB^+^ organoids in CHIR‐treated cultures (Figure 5b). Flow cytometry at day 14 demonstrated that CHIR reduced the proportion of BCs by approximately 49.7% relative to controls while expanding secretory and AT2 cell populations (Figure 5c,d). Prolonged CHIR exposure (>3 passages) nearly eliminated BCs, while CD66c^+^ BCO formation re‐emerged upon CHIR withdrawal (Figure 5e). These findings indicate that CHIR stabilizes secretory and AT2 identities while limiting CD66c^+^ BC formation.

To dissect cell type‐specific effects, we cultured FACS‐sorted secretory cells with or without CHIR. Under control conditions, secretory cells readily formed solid BCOs, whereas CHIR‐treated cultures generated hollow organoids (Figure 5f). Immunofluorescence confirmed maintenance of secretory markers (CC10, SFTPB) alongside reduced KRT5^+^ BCs (Figure 5f). Flow cytometry revealed a substantial decrease in CD66c^+^ NGFR^+^ ITGA6^+^ BCs, and RT‐qPCR revealed significant downregulation of *KRT5* mRNA (Figure 5g–i). Notably, recombinant WNT3A did not recapitulate the inhibitory effects of CHIR (Figure S14a,b), suggesting that CHIR‐mediated suppression extends beyond canonical Wnt/β‐catenin activation. Similarly, CHIR preserved AT2 identity in AT2‐derived organoids, whereas BCO formation occurred in the absence of CHIR (Figure 5j, Figure S13c–f), consistent with the increased AT2 representation observed in CHIR‐treated DLOs. Single‐cell profiling of secretory‐derived organoids cultured with or without CHIR further revealed divergent lineage outcomes. CHIR treatment reduced BC clusters and nearly abolished CD66c^+^ BCs (Figure 5k, Figure S13a), while maintaining secretory identity and redirecting differentiation trajectories toward alveolar (AT2) lineages, as indicated by pseudotime analysis (Figure S13b). When FACS‐sorted CD66c^−^ BCs were treated with CHIR, these cells showed increased differentiation toward secretory lineages (Figure S15). Collectively, these results demonstrate that CHIR preserves secretory and AT2 identities while inhibiting their transdifferentiation into CD66c^+^ BCs.

### 2.7 CHIR99021 Stabilizes AT2 and Secretory Cell Identities Associated with Enhanced ERBB4‐Related Signaling Programs

**FIGURE 6:**
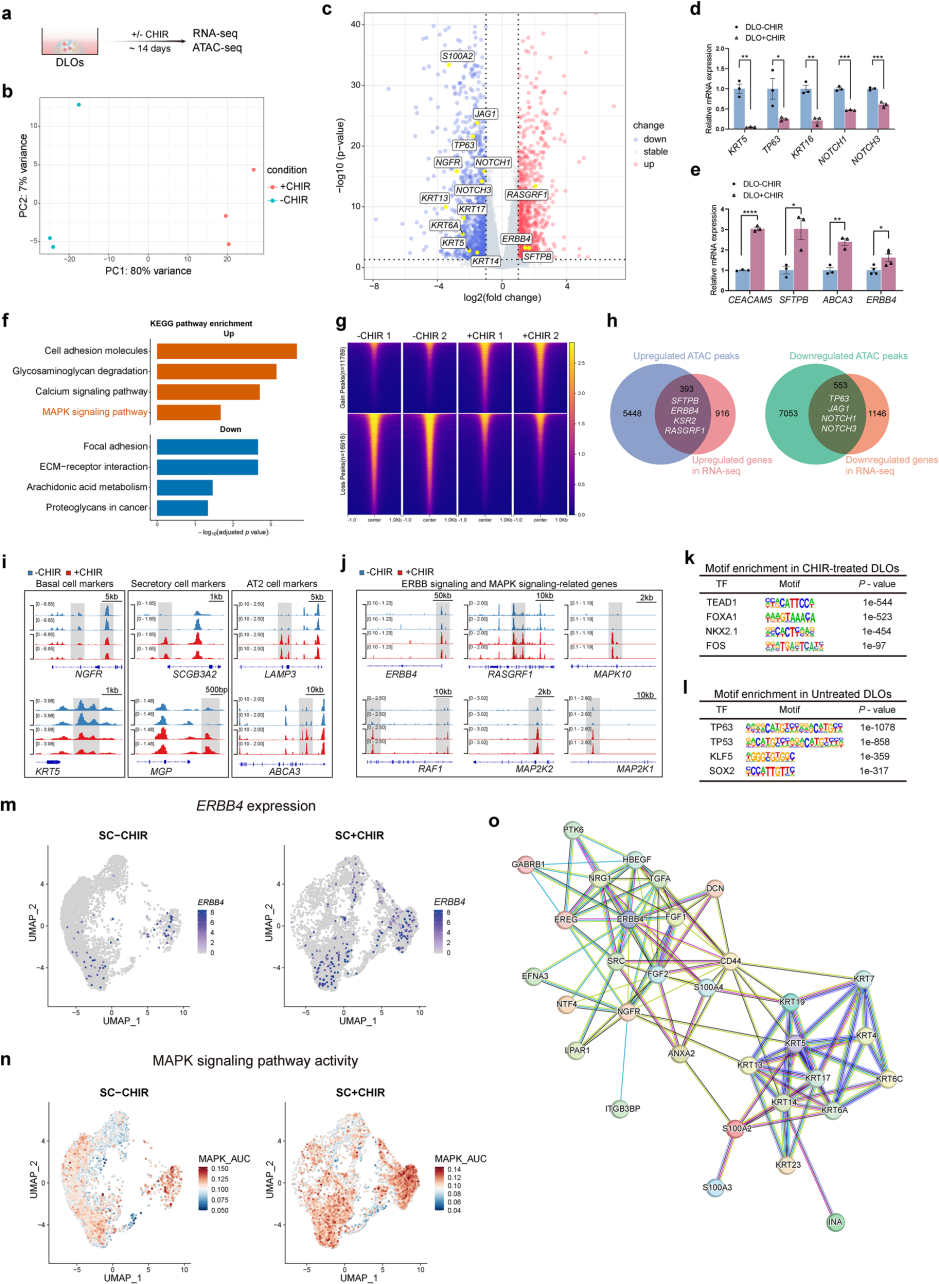
CHIR preserves secretory and AT2 cell identities through activation of ERBB4‐MAPK signaling. (a) Schematic diagram of the experimental workflow: distal lung organoids (DLOs) were cultured for 14 days with or without CHIR99021, followed by bulk RNA‐seq and ATAC‐seq. (b) Principal component analysis (PCA) of RNA‐seq data showing clear separation between −CHIR and +CHIR groups (*n* = 3). (c) Volcano plot of differentially expressed genes (DEGs) between −CHIR and +CHIR DLOs (|log_2_ fold change| > 1). Representative genes are highlighted. (d, e) RT‐qPCR validation of selected DEGs showing reduced expression of basal and NOTCH‐associated genes and increased expression of secretory and AT2 lineage markers, together with *ERBB4*, upon CHIR treatment (*n* = 3). (f) KEGG pathway enrichment analysis of DEGs. Upregulated pathways are shown in red and downregulated pathways in blue (*x*‐axis: −log_10_ adjusted *p*‐value). MAPK signaling was among the most significantly enriched pathways. (g) Heatmaps of ATAC‐seq signals centered on transcription start sites (±1 kb), showing regions with increased (+CHIR) or decreased (−CHIR) chromatin accessibility. (h) Integrated RNA‐seq and ATAC‐seq analysis. Left: overlap between gain peaks and upregulated genes. Right: overlap between loss peaks and downregulated genes, demonstrating coordinated transcriptional and chromatin changes. (i, j) ATAC‐seq tracks of representative loci showing decreased accessibility at basal genes and increased accessibility at secretory, AT2, and ERBB4‐MAPK‐associated loci. (k, l) Motif enrichment analysis showing enrichment of TEAD, FOXA1, NKX2.1, and FOS motifs in +CHIR samples, and basal lineage‐associated motifs in −CHIR samples. (m, n) Single‐cell analysis of secretory‐derived organoids. (m) UMAP plots showing increased *ERBB4* expression in +CHIR relative to −CHIR organoids. (n) UMAP visualization of MAPK pathway activity scores calculated using AUCell, showing elevated MAPK signaling in +CHIR organoids. (o) Protein–protein interaction (PPI) network of DEGs from bulk RNA‐seq, highlighting potential interactions among *ERBB4*, *NRG1*, and basal cell markers, consistent with roles in regulating epithelial plasticity. Data are shown as mean ± SEM where applicable. Statistical significance for RT‐qPCR analyses was determined by unpaired two‐tailed Student's *t*‐test. *n* represents independent biological replicates. *p < 0.05, **p < 0.01, ***p < 0.001, ****p < 0.0001.

To investigate how CHIR maintains secretory and AT2 cell identities, we performed bulk RNA‐seq and ATAC‐seq on human DLOs with or without CHIR treatment (Figure 6a). PCA revealed clear separation between CHIR‐treated and control samples, with PC1 accounting for 80% of the variance (Figure 6b). Differential expression analysis identified 1,146 downregulated genes in CHIR‐treated organoids, including BC markers (*KRT5*, *TP63*, *KRT17*, *NGFR*) and NOTCH pathway components (*JAG1* and *NOTCH1/3*). These findings were validated by RT‐qPCR (Figure 6c,d, Figure S14c). Notably, NOTCH signaling is a central regulator of BC fate [41], and NOTCH3 restricts SPB differentiation toward secretory lineages [17]. In parallel, CHIR treatment upregulated *ERBB4* and multiple downstream effectors, as confirmed by quantitative transcript analysis (Figure 6c,e). Pathway enrichment analysis further revealed enrichment of ERBB4‐associated signaling networks, including MAPK pathways, alongside suppression of focal adhesion and ECM–receptor interaction pathways (Figure 6f). Together, these results suggest that CHIR stabilizes AT2 and secretory identities in association with reduced NOTCH activity and enhanced ERBB4 signaling, consistent with a role in limiting aberrant lineage conversion.

ATAC‐seq profiling revealed extensive chromatin remodeling in CHIR‐treated DLOs, with 11,789 regions showing increased accessibility and 16,918 regions showing decreased accessibility relative to controls (Figure 6g). Integration with RNA‐seq data identified 393 genes exhibiting both transcriptional upregulation and increased chromatin accessibility, including *ERBB4*, the alveolar/secretory marker *SFTPB*, and MAPK regulators (*KSR2*, *RASGRF1*) (Figure 6h). Conversely, 553 downregulated genes exhibited reduced accessibility, including the BC marker *TP63* and NOTCH pathway components (*JAG1*, *NOTCH1*/*3*) (Figure 6h). These patterns were further supported at the locus level: promoters and gene bodies of BC‐associated genes (e.g., *NGFR* and *KRT5*) displayed decreased accessibility, whereas secretory and AT2 markers (e.g., *SCGB3A2*, *LAMP3*) showed increased accessibility in CHIR‐treated DLOs (Figure 6i). Genes encoding ERBB‐MAPK signaling components, such as *MAPK10*, *RAF1*, and *MAP2K1*, also exhibited increased chromatin accessibility consistent with their transcriptional upregulation (Figure 6j).

Motif enrichment analysis of ATAC‐seq data identified transcription factors potentially mediating transcriptional reprogramming in CHIR‐treated DLOs. TEAD‐binding sites, transcriptional effectors previously linked to ERBB4‐YAP signaling [42, 43], were among the most significantly enriched motifs in regions with increased accessibility (Figure 6k). Additional enriched motifs included NKX2.1 and FOXA1, key regulators of lung epithelial differentiation [44, 45, 46]. FOS motifs were also enriched, consistent with activation of MAPK‐dependent transcriptional programs (Figure 6k). In contrast, motifs for transcription factors associated with BC identity, including TP63, KLF5, and SOX2, were enriched in regions with reduced accessibility in CHIR‐treated DLOs (Figure 6l). Untreated DLOs also showed enrichment of TP53 motifs, consistent with stress‐associated programs (Figure 6l). Together, these findings indicate that CHIR99021 reshapes chromatin accessibility landscapes and reinforces lineage‐specific regulatory networks.

scRNA‐seq analysis further corroborated CHIR's regulatory effects. Secretory cell‐derived organoids treated with CHIR displayed increased *ERBB4* expression and elevated MAPK pathway activity scores (Figure 6m,n). *ERBB4* expression was most prominent in AT2 cells and also detectable in secretory cells (Figure 6m, Figure S14e), consistent with its proposed role in maintaining lineage fidelity. Pseudotime analysis showed progressive upregulation of *NRG1* along the secretory/AT2‐to‐basal trajectory (Figure S14f,g). Given *ERBB4* enrichment in AT2 and secretory cells, this pattern suggests potential NRG1‐ERBB4 crosstalk mediated through paracrine interactions. In addition, protein–protein interaction (PPI) network analysis using STRING database (v12.0) positioned the NRG1‐ERBB4 axis and BC markers within the same signaling cluster, further highlighting potential functional connectivity (Figure 6o).

### 2.8 NRG1‐ERBB4 Signaling Suppresses Basal Cell Transdifferentiation from Secretory and AT2 Progenitors

**FIGURE 7:**
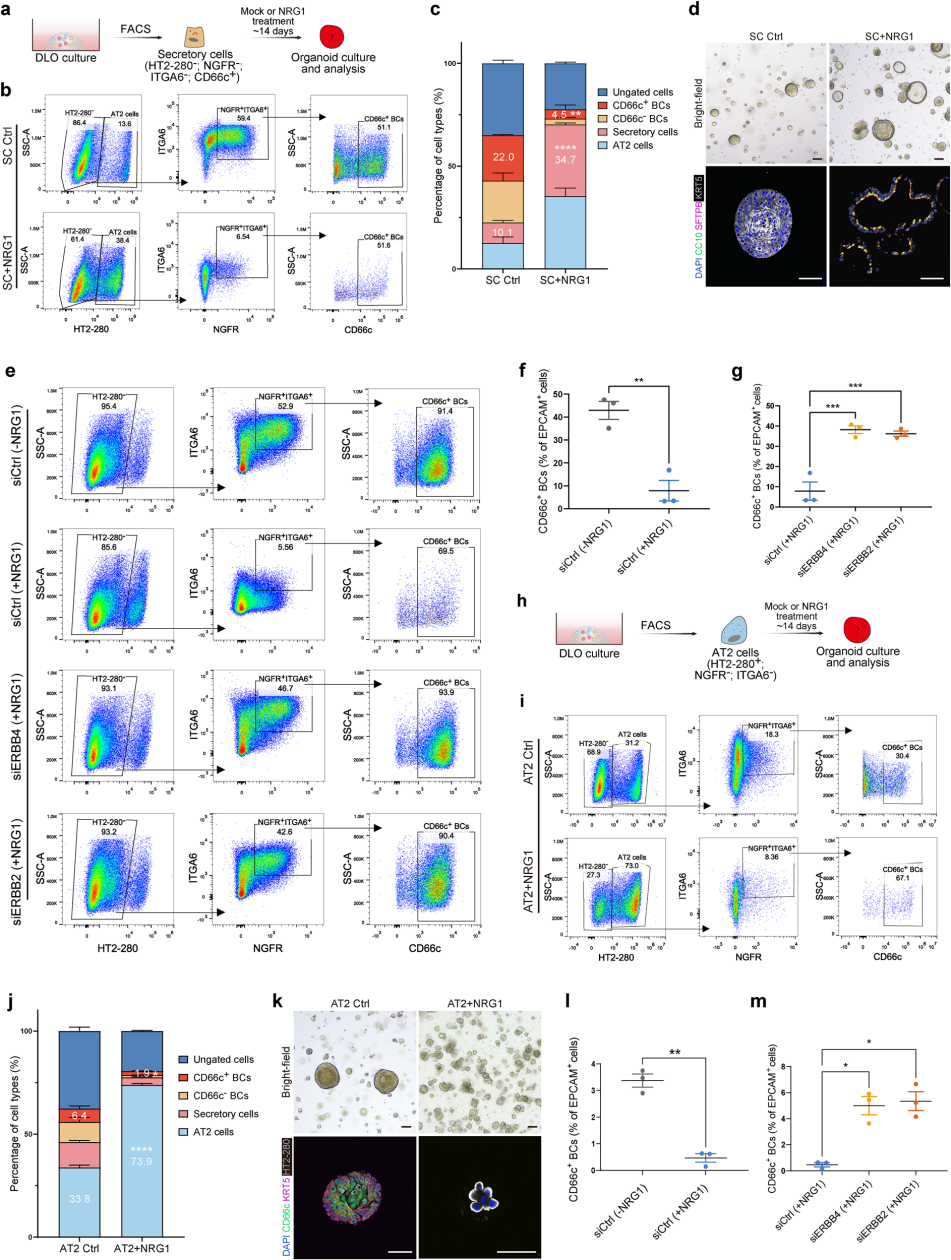
NRG1 signaling inhibits transdifferentiation of secretory and AT2 CELLS Into CD66c^+^ basal cells. (a) Experimental workflow: Secretory cells were isolated by fluorescence‐activated cell sorting (FACS) from distal lung organoids (DLOs) and cultured for ∼14 days in control medium or recombinant NRG1. (b) Representative flow cytometry plots of EPCAM^+^ epithelial cells from secretory cell‐derived organoids showing reduced CD66c^+^ basal cells (BCs) upon NRG1 treatment. (c) Quantification of epithelial subsets in secretory cell‐derived organoids cultured ± NRG1 (*n* = 3). (d) Bright‐field and immunofluorescence (IF) images of secretory‐derived organoids ± NRG1. NRG1 suppressed solid organoid formation and preserved expression of secretory markers (SFTPB, CC10), while reducing KRT5^+^ BCs. Nuclei were counterstained with DAPI. Scale bars, 50 µm. (e) Representative flow cytometry plots showing phenotypic changes of secretory cell‐derived basal cells following siRNA transfection and NRG1 treatment, with siCtrl (−NRG1) included as the untreated control. (f, g) Flow cytometric quantification of CD66c^+^ basal cells among EPCAM^+^ epithelial cells under the indicated conditions (*n* = 3). (f) Comparison between siCtrl (−NRG1) and siCtrl (+NRG1), and (g) comparison between siERBB4 or siERBB2 under NRG1 treatment. (h) Experimental workflow for AT2‐derived organoids: FACS‐sorted AT2 cells (HT2‐280^+^ NGFR^−^ ITGA6^−^) were cultured ± NRG1 for ∼14 days. (i) Flow cytometry plots of EPCAM^+^ cells from AT2‐derived organoids, showing decreased CD66c^+^ BCs after NRG1 treatment. (j) Quantification of epithelial subsets in AT2‐derived organoids cultured ± NRG1 (*n* = 3). (k) Bright‐field and IF images of AT2‐derived organoids cultured ± NRG1 for 14 days. NRG1 promoted cystic morphology, preserved AT2 marker HT2‐280 (gray), and reduced CD66c^+^ KRT5^+^ BCs. Nuclei were counterstained with DAPI (blue). Scale bars, 50 µm. (l, m) Flow cytometric quantification of CD66c^+^ basal cells derived from AT2 cells under the indicated conditions (*n* = 3). (l) Comparison between siCtrl (−NRG1) and siCtrl (+NRG1), and (m) comparison between siERBB4 or siERBB2 under NRG1 treatment. Data are shown as mean ± SEM. Statistical significance was determined by unpaired two‐tailed Student's *t*‐test for comparisons between two groups and by one‐way ANOVA with appropriate multiple comparisons tests for analyses involving three groups. *n* represents independent biological replicates. *p < 0.05, **p < 0.01, ***p < 0.001, ****p < 0.0001.

To investigate the role of NRG1‐ERBB4 signaling in epithelial plasticity, we treated secretory cell‐derived organoids with recombinant NRG1 (Figure 7a). NRG1‐treated organoids maintained secretory morphology and exhibited significantly reduced transdifferentiation into CD66c^+^ BC, as confirmed by immunofluorescence staining and flow cytometric quantification (Figure 7b–d, Figure S18a). To evaluate the functional impact of lineage stabilization, NRG1‐treated and control secretory organoids were subcutaneously engrafted into NSG mice (Figure S16a). NRG1 treatment significantly reduced fibrotic remodeling at day 18 post‐engraftment, evidenced by reduced collagen deposition, decreased accumulation of αSMA^+^ myofibroblast, and a lower SFI (Figure S16b,c). Bulk RNA‐seq analysis showed downregulation of BC markers (*KRT5*, *TP63*), along with upregulation of secretory markers (*SCGB3A1*, *LCN2*) and MAPK‐related genes (Figure S18b–d), which was further validated by RT‐qPCR (Figure S18e,f).

Pharmacological inhibition of ERBB4 signaling with AG1478 attenuated the suppressive effect of NRG1 on CD66c^+^ BC generation, leading to a significant increase in CD66c^+^ BCs (Figure S18h,i). Moreover, inhibiting MAPK signaling with the MEK inhibitor U0126 partially restored CD66c^+^ BC formation in the presence of NRG1, suggesting that MAPK activity contributes to NRG1‐mediated lineage stabilization in secretory progenitors (Figure S18j,k). To examine the requirement for ERBB receptors, secretory‐derived organoids were treated with siRNA targeting *ERBB4* or *ERBB2*, with or without NRG1. Efficient transfection was confirmed using a FAM‐labeled control siRNA (Figure S17a), and knockdown efficiency was validated by RT‐qPCR (Figure S17b–e). Depletion of either receptor significantly increased the proportion of CD66c^+^ BCs compared with NRG1‐treated controls, indicating that both ERBB4 and its co‐receptor ERBB2 are involved in NRG1‐mediated suppression of CD66c^+^ BC expansion (Figure 7e–g). Notably, while ERBB2 is broadly expressed in multiple lung epithelial populations (Figure S21), ERBB4 expression is relatively enriched in AT2 and secretory cells, suggesting its role as the lineage‐relevant receptor for NRG1‐dependent epithelial stabilization.

NRG1 also regulates AT2 plasticity. In AT2‐derived organoids, NRG1 treatment significantly reduced CD66c^+^ BC generation and preserved AT2 morphology, as shown by flow cytometry, bright‐field imaging, and immunostaining (Figure 7h–k). Transcriptomic profiling revealed upregulation of AT2 identity genes and concurrent downregulation of BC and SPB markers, which was validated by RT‐qPCR (Figure S19a–e). In addition, MAPK pathway activity was enhanced (Figure S19f,g), and Gene Ontology analysis further showed enrichment of MAPK‐related pathways and BMP receptor binding. In contrast, TGFβ, Hedgehog/Smoothened, and inflammatory signaling pathways associated with pathological epithelial plasticity were suppressed [8, 47] (Figure S19f). Consistent with these transcriptional changes, pharmacological inhibition of MAPK or BMP signaling significantly increased CD66c^+^ BC generation in NRG1‐treated AT2 organoids (Figure S19h,i). These results suggest that NRG1‐mediated stabilization of AT2 identity is linked to MAPK signaling activation and modulation of BMP‐associated pathways. Furthermore, siRNA‐mediated knockdown of *ERBB4* or *ERBB2* reversed NRG1's inhibitory effect on CD66c^+^ BC formation from AT2 progenitors (Figure 7l,m, Figure S17f).

Together, these findings demonstrate that NRG1 suppresses CD66c^+^ BC transdifferentiation from both secretory and AT2 epithelial progenitors via ERBB4‐dependent signaling, involving ERBB2. This highlights a conserved mechanism of lineage stabilization across distal lung epithelial compartments.

### 2.9 Spatial Proximity of CD66c^+^ Basal Cells to Myofibroblasts Coincides with Epithelial ERBB4 Loss in IPF

**FIGURE 8:**
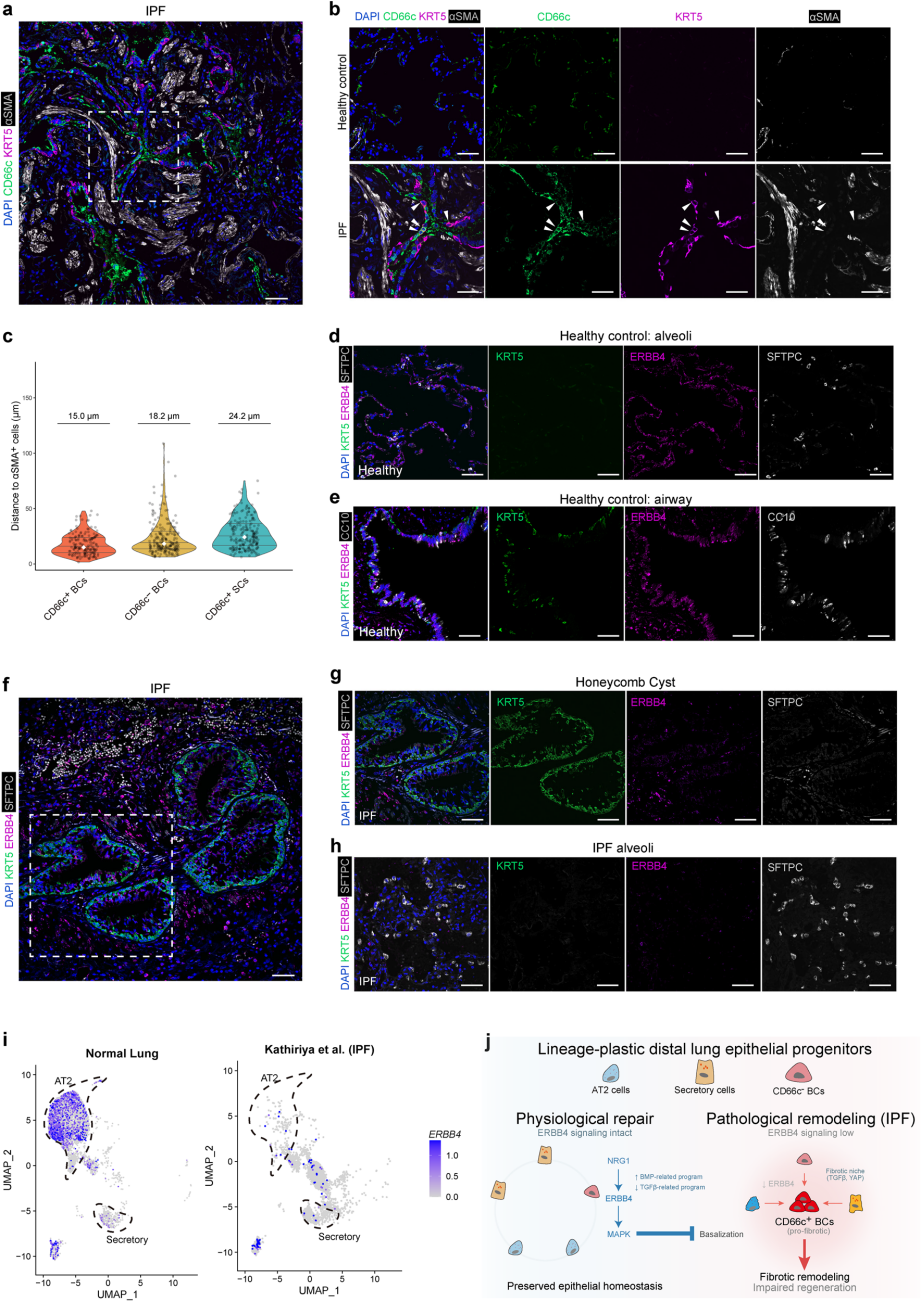
Loss of ERBB4 expression in epithelial cells of IPF lungs. (a) Multiplex immunofluorescence (IF) staining of lung tissue from patients with idiopathic pulmonary fibrosis (IPF) showing nuclei (DAPI, blue), CD66c (green), KRT5 (magenta), and αSMA (gray). The white dashed box marks the region shown in panel (b). Scale bars, 50 µm. (b) Higher‐magnification views of the boxed region in panel (a) show CD66c^+^KRT5^+^ basal cells (BCs; arrows) adjacent to αSMA^+^ myofibroblasts in IPF lungs (lower row). Representative healthy lung tissue is shown in the upper row for comparison. Scale bars, 50 µm. (c) Spatial proximity of epithelial subsets to αSMA^+^ regions in IPF lungs. Violin plots show the distribution of nearest‐neighbor distances from epithelial cells to αSMA^+^ myofibroblasts for CD66c^+^ BCs, CD66c^−^ BCs, and CD66c^+^ secretory cells (SCs; CD66c^+^ KRT5^−^). White squares and thick lines denote medians and interquartile ranges, respectively. Median distances (µm) are shown above. Data were obtained from multiplex IF images of two IPF patients (*n* = 2). (d) Multiplex IF of normal alveoli shows strong ERBB4 (magenta) colocalized with the AT2 marker SFTPC (white), with minimal or no KRT5^+^ BCs (green). Nuclei: DAPI (blue). Scale bars, 50 µm. (e) In normal airways, ERBB4 (magenta) is expressed in CC10^+^ SCs (green) but is minimal or absent in KRT5^+^ BCs (white). Nuclei: DAPI (blue). Scale bars, 50 µm. (f) Low‐magnification view of IPF parenchyma reveals honeycomb cysts labeled for DAPI (blue), KRT5 (green), ERBB4 (magenta), and SFTPC (gray). Scale bars, 50 µm. (g) Enlarged view of the boxed region in panel (f) highlights honeycomb cysts densely lined with KRT5^+^ BCs and reduced ERBB4 expression, with few SFTPC^+^ AT2 cells. Scale bars, 50 µm. (h) Residual alveolar regions in IPF lungs exhibit reduced ERBB4 expression in SFTPC^+^ AT2 cells. Scale bars, 50 µm. (i) UMAP plots of scRNA‐seq data comparing normal distal lung epithelium (left, this study) and IPF lung tissue (right, Kathiriya et al., 2022). ERBB4 expression is enriched in normal epithelium, particularly in AT2 and SC clusters (outlined), but is markedly reduced in IPF lungs. (j) Proposed model of ERBB4 signaling in regulating epithelial plasticity during lung injury. Distal lung epithelial progenitors (AT2 cells, secretory cells, and CD66c^−^ BCs) exhibit lineage plasticity and can converge toward a CD66c^+^ basal state under injury‐associated conditions. During physiological repair, NRG1‐ERBB4‐MAPK signaling maintains epithelial identity and restrains aberrant basalization, in association with balanced BMP‐ and TGFβ‐related programs. In IPF, attenuation of ERBB4 signaling coincides with increased generation of pro‐fibrotic CD66c^+^ BCs within disease‐associated niches enriched for TGFβ‐ and YAP‐related activity, contributing to pathological remodeling and impaired regeneration.

To assess the clinical relevance of CD66c^+^ BCs, we performed multiplex immunofluorescent staining on lung tissues from patients with IPF and healthy controls. CD66c^+^ KRT5^+^ dual‐positive BCs were detected exclusively within fibrotic regions of IPF lungs, where they were frequently juxtaposed to αSMA^+^ myofibroblasts (Figure 8a,b, Figure S20a). These cells were absent in control tissues. Spatial quantification demonstrated that CD66c^+^ BCs were located closer to αSMA^+^ fibroblasts (median distance: 15.0 µm, IQR 10.1–23.5) than CD66c^−^ BCs (18.2 µm, IQR 13.4–28.3) or CD66c^+^ secretory cells (24.2 µm, IQR 15.4–35.1) (Figure 8c). This spatial enrichment within fibrotic niches suggests a potential association between CD66c^+^ BCs and fibroblast activation.

We next examined ERBB4 expression in lung tissues. In control lungs, strong membrane‐associated ERBB4 staining was detected in SFTPC^+^ AT2 cells within alveolar regions and in club/secretory cells of bronchiolar epithelium, whereas KRT5^+^ BCs exhibited minimal ERBB4 expression (Figure 8d,e, Figure S20b). In IPF lungs, ERBB4 expression was markedly reduced across pathological regions. Honeycomb cysts enriched for KRT5^+^ BCs showed substantially diminished ERBB4 staining (Figure 8f,g), and residual alveolar regions similarly displayed reduced epithelial ERBB4 signals (Figure 8h). Analysis of published scRNA‐seq datasets further corroborated these findings, revealing *ERBB4* expression in AT2 and secretory cells of normal lungs but markedly decreased levels in IPF epithelia (Figure 8i).

To determine whether the spatial enrichment of CD66c^+^ BCs observed histologically was reflected at the transcriptomic level, we analyzed a publicly available 10× Visium spatial transcriptomics dataset derived from mildly fibrotic regions of IPF lung tissue [31] (Figure S22a). Histopathological annotations from the original study were used to delineate diseased and relatively preserved alveolar regions (Figure S22b). Spatial mapping of inferred BCs revealed their preferential localization within disease‐associated regions (Figure S22c). Consistently, SPB marker genes, including *CEACAM6, SERPINB1, GDF15*, and *RAB11FIP1*, were enriched in fibrotic areas (Figure S22d). In contrast, markers of other BC subtypes showed more heterogeneous and less spatially restricted distributions, including AB markers (*FOSB, ATF3*), the MPB marker *LGALS1*, and the PB marker *MKI67* (Figure S22e).

We further examined the spatial distribution of genes associated with the NRG1‐ERBB4‐MAPK signaling axis. Consistent with our immunostaining results, NRG1, ERBB4, and downstream MAPK‐related genes exhibited generally low expression across diseased regions (Figure S22f). Conversely, genes associated with signaling pathways implicated in epithelial plasticity and fibrotic remodeling, including TGFβ (TGFB1, TGFBR2), NF‐κB (CCL2), and Hippo (YAP1) pathways [8, 47, 48], were prominently enriched within diseased regions (Figure S22g). Spatial pathway activity scoring further clarified these regional differences (Figure S23). Disease‐associated regions were characterized by reduced MAPK signaling, with BMP pathway activity showing a similar attenuation (Figure S23a,b). In contrast, TGFβ and Hippo signaling were elevated in the same regions (Figure S23c,d). Together, these spatial patterns reveal a regionally segregated signaling states in IPF lungs.

Collectively, these findings demonstrate that CD66c^+^ BC‐associated transcriptional programs preferentially localize to disease‐associated niches marked by attenuated ERBB4‐MAPK signaling, consistent with a microenvironment permissive for aberrant BC state transitions.

## 3 Discussion

This study identifies aberrant transdifferentiation of distal lung epithelial progenitors, particularly secretory and AT2 cells, into CD66c^+^ BCs as an epithelial mechanism that may contribute to fibrotic remodeling. Using human DLOs, we observed robust emergence and expansion of CD66c^+^ BCs, a BC subset previously reported to be enriched in IPF lungs [17, 25] yet difficult to interrogate experimentally. Although CD66c (*CEACAM6*) has often been viewed as a marker of secretory‐lineage programs [49], recent single‐cell studies have described a CD66c^+^ TP63^+^ KRT5^+^ BC population enriched in fibrotic regions [17, 25], suggesting that CD66c expression may accompany pathological epithelial reprogramming rather than stable lineage identity.

The DLO system recapitulates the emergence of this state under injury‐like culture conditions and enables functional interrogation of its downstream consequences. In both co‐culture and conditioned‐medium settings, CD66c^+^ BCs promoted fibroblast proliferation and myofibroblast activation, and xenograft assays further demonstrated increased collagen deposition and αSMA^+^ stromal accumulation at CD66c^+^ BC engraftment sites. Together, these findings provide functional evidence that CD66c^+^ BCs are not merely a bystander marker of injury but possess intrinsic pro‐fibrotic activity capable of shaping stromal responses.

Our lineage‐resolved analyses further indicate that CD66c^+^ BCs arise through multiple epithelial routes that converge on a shared transcriptional state. Pre‐existing CD66c^−^ BCs efficiently acquired CD66c expression, while non‐basal progenitors, including secretory and AT2 cells, also generated CD66c^+^ BCs under organoid‐forming conditions. The higher apparent efficiency observed from secretory cells relative to AT2 cells is consistent with prior observations in murine injury models, in which secretory‐lineage cells can enter p63^+^ progenitor‐like states following epithelial damage [30]. Together with reports that H2‐K1^high^ secretory populations contribute to alveolar regeneration in mice [28], these findings highlight secretory cells as a highly plastic progenitor pool within the distal lung epithelium. Notably, the predominance of BC‐like states during DLO cultures [50, 51] likely reflects plasticity that becomes unmasked or amplified ex vivo, rather than a direct mirror of steady‐state tissue composition. Importantly, across progenitor sources, CD66c^+^ BCs converged toward a conserved transcriptional program resembling IPF‐associated metaplastic basal states, supporting the concept that distinct injury‐adapted progenitors can funnel into a common pathological endpoint.

Mechanistically, our multi‐omic analyses suggest that CHIR99021 suppresses CD66c^+^ BC formation and preserves secretory and AT2 identities through coordinated transcriptional and epigenetic remodeling. Although CHIR is widely used as a canonical WNT pathway activator, the inability of recombinant WNT3A to phenocopy its effects, together with the chromatin and transcriptomic changes observed here, indicates that its influence on epithelial fate cannot be attributed to β‐catenin signaling alone. Instead, CHIR treatment was accompanied by increased regulatory activity at core epithelial lineage determinants (e.g., NKX2‐1 and FOXA1), induction of MAPK‐associated transcriptional programs, and coordinated upregulation of *ERBB4* alongside repression of basal and NOTCH‐associated regulatory modules. These findings support a framework in which CHIR reshapes the epithelial regulatory landscape and engages ERBB4‐linked signaling programs to favor lineage stabilization and restrict aberrant basal conversion under regenerative stress.

Building on this framework, we identify the NRG1‐ERBB4 axis as a signaling module capable of actively constraining pathological basal conversion. Exogenous NRG1 markedly reduced CD66c^+^ BC generation from both secretory and AT2 progenitors, attenuated stromal remodeling in xenograft assays, and its protective effect was diminished by ERBB4 inhibition or *ERBB4* knockdown. These functional perturbation experiments support a model in which ERBB4‐dependent signaling, together with downstream MAPK activity, stabilizes distal epithelial identity and limits entry into the CD66c^+^ basal state (Figure 8j). Finally, analysis of human IPF lung tissues places this regulatory logic in a clinically relevant spatial context. CD66c^+^ BCs were confined to disease‐associated niches and localized in close proximity to αSMA^+^ myofibroblasts, coinciding with reduced epithelial ERBB4 expression in honeycomb cysts and residual alveolar regions. Spatial transcriptomic analyses further supported regional signaling alterations in diseased areas, characterized by attenuation of ERBB4/MAPK‐ and BMP‐associated activity alongside elevation of profibrotic programs. Together, these observations suggest that loss of ERBB4‐linked lineage‐stabilizing inputs within fibrotic microenvironments may permit aberrant basal state transitions and reinforce epithelial‐stromal feedback that sustains remodeling.

In summary, our study defines CD66c^+^ BCs as a convergent pathological epithelial state with intrinsic pro‐fibrotic potential arising from multiple distal epithelial progenitors. By establishing ERBB4 signaling as a central axis that stabilizes epithelial identity and restricts pathological basal conversion, our work provides mechanistic insight into how epithelial plasticity becomes dysregulated in IPF. These findings further raise the possibility that reinforcing NRG1‐ERBB4 signaling may represent a therapeutic strategy to preserve epithelial stability and limit fibrotic progression.

## 4 Experimental Section

### 4.1 Animals

All animal experiments were approved by the Animal Care and Use Committee of Fudan University (approval number: 2025JS140). Male NOD.Cg‐*Prkdc* ^scid^ *Il2rg* ^tm1Wjl^/SzJ (NSG) mice, 7–8 weeks of age, were purchased from the Shanghai Model Organisms Center (China). Mice were housed in a specific pathogen‐free (SPF) facility under controlled environmental conditions with ad libitum access to food and water.

### 4.2 Cell Line

The human lung fibroblast cell line MRC‐5 was obtained from the American Type Culture Collection (ATCC, Manassas, VA, USA). Cells were maintained in DMEM/F12 medium (Gibco) supplemented with 10% fetal bovine serum (FBS; Lonsera, China) and 1% penicillin‐streptomycin at 37°C in a humidified incubator with 5% CO_2_. The absence of mycoplasma contamination was confirmed by PCR‐based testing.

### 4.3 Human Tissues

Human lung tissue collection was approved by the Institutional Ethics Committee of Zhongshan Hospital, Fudan University (approval number: B2024‐463R). Written informed consent was obtained from all patients, confirming voluntary donation of discarded lung tissues for research purposes on severe lung injury using organoid technology. Exclusion criteria included pregnancy or lactation, severe psychiatric disorders, and pathological stage IV lung cancer. All research procedures complied with the principles of the Declaration of Helsinki.

### 4.4 Organoid Culture

Human lung specimens were maintained in ice‐cold Advanced DMEM/F12 medium (Gibco) supplemented with Antibiotic–Antimycotic solution (Gibco) until further processing. Tissues were minced and enzymatically digested at 37°C for 30 min (110 rpm) in a rotating incubator using a digestion cocktail containing 1.68 mg/mL collagenase type I (Worthington, LS004194), 5 U/mL Dispase (Corning, 354235), and 10 U/mL DNase I (Sigma, D5025). Following digestion, the cell suspension was filtered through a 100‐µm cell strainer and washed with DMEM/F12 medium supplemented with 10% fetal bovine serum (FBS). Cells were pelleted by centrifugation at 300× *g* for 5 min, subjected to red blood cell lysis for 3 min, resuspended in Growth Factor Reduced Matrigel (Corning), and seeded into tissue culture‐treated 24‐well plates (Jet Biofil, China). Organoids were maintained in DLO expansion medium, the detailed composition of which is provided in Table S1. Medium was refreshed every 2–3 days, and organoids were cultured at 37°C in a humidified incubator with 5% CO_2_.

For functional perturbation experiments, organoids were treated with CHIR99021 (3 µM; MedChemExpress (MCE), USA), recombinant human NRG1 (50 ng/mL; Sino Biological, China), or the ERBB4 inhibitor AG1478 (5 µM; T2047, TargetMol, USA). For pathway modulation, BMP signaling was inhibited by withdrawing Noggin (Nog; Sino Biological, China) and adding the BMP type I receptor inhibitor LDN193189 (LDN; 100 nM; T1935, TargetMol, USA), while the MEK inhibitor U0126 (2.5 µM; T21332, TargetMol, USA) was applied to block MAPK signaling.

### 4.5 Immunofluorescent Staining

Organoids were harvested using Cell Recovery Solution (Corning, USA) and gently dissociated into clusters, fixed in 4% paraformaldehyde (PFA) in PBS for 30 min at 4°C, embedded in 3% agarose and subsequently paraffin‐embedded. Sections (4–5 µm) were deparaffinized and rehydrated through graded ethanol solutions, followed by antigen retrieval in either 10 mM sodium citrate (pH 6.0) or Tris‐EDTA buffer (pH 9.0) using a pressure cooker for 20 min. After cooling to room temperature, sections were blocked with 5% normal donkey or goat serum for 1 h and incubated overnight at 4°C with primary antibodies diluted in 1% BSA. Primary antibodies included: HT2‐280 (1:200, Terrace Biotechnologies, TB‐27AHT2‐280), KRT5 (1:500, ABclonal, 9661), CD66c (1:50, BioLegend, 365002), HBEGF (1:200, Bioss, bs‐3576R), MUC1 (1:200, BD Pharmingen, 555925), αSMA (1:500, Sigma, C6198), Ki67 (1:500, BD Biosciences, 550609), HER2 (1:200, Abcam, ab134182), SCGB1A1 (1:500, R&D Systems, MAB4218‐SP), PDPN (1:200, BioLegend, 337001), SFTPB (1:200, Santa Cruz, sc‐133143), SFTPC (1:200, Millipore, AB3786; alternatively, Abcam, ab312850), ERBB4 (1:50, Invitrogen, MA1‐861), and CDH1 (1:400, BD Biosciences, 610181). [Correction added on 16 April 2026 after first online publication: The antibody “NAPSA (1:200, ABclonal, A5594)” is removed because it was not used in the experiments reported in this study.]

After washing, sections were incubated with fluorochrome‐conjugated secondary antibodies and DAPI (Solarbio) diluted in 1% BSA for 1 h at room temperature. Secondary antibodies included: CoraLite488 donkey anti‐mouse IgG (H+L) (Proteintech, SA00013‐5), CoraLite594 donkey anti‐rabbit IgG (H+L) (Proteintech, SA00013‐8), CoraLite488 donkey anti‐rabbit IgG (H+L) (Proteintech, SA00013‐6), CoraLite647 donkey anti‐rabbit IgG (H+L) (Proteintech, SA00014‐7), CoraLite594 donkey anti‐mouse IgG (H+L) (Proteintech, SA00013‐7), Alexa Fluor 647 donkey anti‐goat IgG (H+L) (Jackson ImmunoResearch, 305‐605‐003), Alexa Fluor 647 donkey anti‐rat IgG (H+L) (Jackson ImmunoResearch, 712‐605‐153), Alexa Fluor 647 donkey anti‐chicken IgY (H+L) (Jackson ImmunoResearch, 703‐605‐155), Cy3 donkey anti‐chicken IgG (H+L) (Jackson ImmunoResearch, 703‐166‐155), and Cy3 donkey anti‐rat IgG (H+L) (Jackson ImmunoResearch, 712‐165‐153). Sections were then washed, mounted with an antifade mounting medium, and imaged using an FV3000 confocal microscope (Olympus, Japan).

### 4.6 RNA Purification and RT‐qPCR

Organoids were lysed in RL buffer (Tiangen Biotech, China), and total RNA was extracted using the Micro RNA Extraction Kit (Tiangen Biotech). RNA quality was assessed with NanoDrop spectrophotometry. For reverse transcription, 500 ng of total RNA was converted into cDNA using the FastKing Reverse Transcription Kit with gDNA removal (Tiangen Biotech). RT‐qPCR was performed with SYBR Green PCR Master Mix (Bimake, USA) on a CFX384 Touch Real‐Time PCR Detection System (Bio‐Rad, USA). Relative mRNA expression levels were calculated using the 2^−ΔΔCt^ method, with *GAPDH* as the internal control. Primer sequences are provided in Table S2.

### 4.7 Organoid Dissociation and FACS

Organoids were dissociated into single cells as follows. Culture medium was aspirated, and 400 µL TrypLE Express (Gibco) was added per well. To disrupt Matrigel, the suspension was pipetted vigorously 6–10 times with a P1000 pipette. Dissociation was monitored under an inverted bright‐field microscope and repeated until no intact organoids remained. Cells were resuspended in FACS buffer (PBS supplemented with 2% FBS, 5 µM EDTA, and Antibiotic‐Antimycotic) and incubated with the anti‐HT2‐280 antibody (1:100, Terrace Biotechnologies) for 30 min at 4°C. After washing, cells were incubated for 30 min at 4°C with AF488 goat anti‐mouse IgM secondary antibody (1:200, Invitrogen, A‐21042) together with directly conjugated antibodies appropriate for each staining panel. These included: anti‐NGFR conjugated to PE/Cy7 (BioLegend, 345110) or AF700 (BioLegend, 345117), anti‐CD49f‐APC (BioLegend, 313615), anti‐CD66c‐PE (BioLegend, 386105), and anti‐CD326 conjugated to either PerCP/Cy5.5 (BioLegend, 369803) or BV421 (BioLegend, 369822), depending on the staining panel. Dead cells were excluded using Fixable Viability Dye eFluor506 (1:1000, eBioscience). Following filtration through a 40 µm strainer, single cells were analyzed or sorted on an MA900 flow cytometer (Sony, Japan) following exclusion of doublets and dead cells. FACS‐sorted cells were seeded at 10,000 cells per well in Matrigel in 24‐well plates and cultured in expansion medium for 14 days at 37°C in a humidified incubator with 5% CO_2_.

### 4.8 siRNA‐Mediated Knockdown

For siRNA‐mediated gene silencing, FACS‐sorted AT2 and secretory cells were transfected with siRNAs targeting *ERBB4* or *ERBB2*, or with a non‐targeting control siRNA (siCtrl). Individual siRNAs targeting *ERBB4* (si‐h‐ERBB4_001/002/003) or *ERBB2* (si‐h‐ERBB2_001/003/011) (RiboBio, China) were used either individually or as pooled siRNAs, as indicated. A FAM‐labeled control siRNA (MCE) was included to monitor transfection efficiency. Transfections were performed using Lipofectamine RNAiMAX (Thermo Fisher Scientific) according to the manufacturer's instructions. Briefly, siRNAs and RNAiMAX were diluted in Opti‐MEM, mixed gently, and incubated at room temperature for 10–20 min to allow formation of siRNA–lipid complexes, which were then added to the sorted cells plated in 24‐well low‐attachment plates (final siRNA concentration, 80 nM). After 6–8 h, the medium was replaced with fresh complete organoid culture medium.

Knockdown efficiency of individual siRNAs was validated by RT‐qPCR at 72 h post‐transfection, and pooled siRNAs were subsequently used for all functional experiments. For phenotypic analyses, cells were maintained in organoid culture and collected at day 7 post‐transfection for downstream assays, including flow cytometry. Where indicated, recombinant human NRG1 was added after transfection for functional assays.

### 4.9 Xenografts in Immunodeficient Mice

For xenografts, 1 × 10^6^ FACS‐sorted organoid‐derived cells were resuspended in 80% Matrigel (Corning) to a final volume of 100 µL. The suspension was kept on ice and injected subcutaneously into the dorsal flank of each mouse using a 1‐mL insulin syringe. The contralateral flank was injected with Matrigel containing CD66c^−^ BCs as a control. Skin tissues at the injection sites were harvested 2–3 weeks post‐transplantation for histological analysis.

### 4.10 CD66c^+^ Basal Cell Organoid and MRC‐5 Co‐culture

FACS‐sorted CD66c^+^ BCs (HT2‐280^−^ ITGA6^+^ NGFR^+^ CD66c^+^) were expanded as organoids until sufficient growth was achieved. For co‐culture experiments, 16,000 MRC‐5 cells were seeded into 24‐well plates one day before co‐culture to achieve ∼40%–50% confluence. Organoids were dissociated with TrypLE Express (Gibco) and co‐cultured with fibroblasts in a 1:1 mixture of fibroblast medium and organoid growth medium at 37°C in a humidified incubator with 5% CO_2_. Co‐cultures were maintained for 5 days, after which cells were collected for downstream analyses.

### 4.11 Treatment of MRC‐5 Cells with CD66c^+^ Basal Cell‐Conditioned Medium

CD66c^+^ BC organoids were cultured in 24‐well plates for 5 days, with medium replaced every other day. On day 5, conditioned medium was collected, centrifuged at 300× *g* for 5 min to remove cellular debris, and filtered through a 0.45‐µm filter. MRC‐5 fibroblasts were seeded and cultured to approximately 50% confluence. On day 2 of fibroblast culture, 50% of the fibroblast culture medium was replaced with CD66c^+^ BC‐conditioned medium, while control wells received an equal volume of fresh organoid medium. Cells were further incubated for 5 days before collection for RT‐qPCR and immunostaining.

### 4.12 Masson's Trichrome Staining

Collagen deposition was assessed using a Masson's trichrome staining kit (Solarbio, G1340) according to the manufacturer's instructions. After deparaffinization and rehydration, sections were sequentially stained with hematoxylin (5 min), Ponceau S (5 min), phosphomolybdic acid (2 min), and aniline blue (2 min), followed by dehydration and mounting with neutral resin. Images were acquired in bright‐field mode using a 20× objective on a Leica DM4B microscope (Leica Microsystems, Wetzlar, Germany).

### 4.13 Stromal Fibrosis Index Calculation

To assess stromal fibrotic changes, multiplex immunofluorescence was performed on mouse skin sections using antibodies against CDH1 and αSMA, with DAPI as a nuclear counterstain. Subcutaneous regions were manually outlined based on tissue morphology and DAPI localization. The stromal compartment was defined by digitally excluding CDH1‐positive epithelial areas. Within this compartment, the SFI was calculated as the proportion of αSMA‐positive area relative to the total stromal area. Images were acquired using a Zeiss LSM880 confocal microscope (Carl Zeiss, Germany). All morphometric analyses, including thresholding (with a consistent threshold applied across images) and area quantification, were performed using ImageJ (NIH, version 1.54).

### 4.14 Hematoxylin and Eosin Staining

H&E staining was carried out using a commercial kit (Solarbio, G1120). After deparaffinization in xylene and rehydration through graded ethanol solutions, sections were stained with hematoxylin (5 min) followed by eosin (5 min), dehydrated, and mounted with neutral resin. Images were acquired in bright‐field mode using a light microscope (Carl Zeiss, Germany).

### 4.15 ATAC‐seq and RNA‐seq Library Preparation and Sequencing

ATAC‐seq libraries were prepared from 5 × 10^4^ to 1 × 10^5^ cells using the Hyperactive ATAC‐seq Library Prep Kit for Illumina (Vazyme, China) according to the manufacturer's instructions. For RNA‐seq, total RNA was extracted, and only samples with an RNA integrity number (RIN) ≥ 8.0 were used. Poly(A)^+^ mRNA was purified using magnetic beads (Vazyme), fragmented, and subjected to library preparation according to the manufacturer's protocol. Library quality was assessed using an Agilent 2100 Bioanalyzer (Agilent Technologies, USA). Sequencing was performed on an Illumina NovaSeq 6000 platform, generating more than 20 million paired‐end 150‐bp reads per sample.

### 4.16 scRNA‐seq Library Preparation and Data Preprocessing

Single‐cell suspensions from organoids were prepared according to the 10× Genomics protocol (CG000315). Approximately 8,000–10,000 cells per sample were loaded onto a Chromium Next GEM Chip G using the Chromium Single Cell 3′ Library Kit v3.1 (10× Genomics, USA). Libraries were sequenced on an Illumina HiSeq 6000 platform to generate 150‐bp paired‐end reads. Raw sequencing data were processed with Cell Ranger software (v6.0.2, 10× Genomics), including demultiplexing, barcode assignment, and UMI counting. Reads were aligned to the human genome (hg38) using the reference dataset refdata‐gex‐GRCh38‐2020‐A.

Downstream analyses were performed in R (v4.2.2) using Seurat (v4.3.0). Cell Ranger outputs were imported into Seurat with the Read10X_h5 function. Quality control was applied to exclude low‐quality cells, defined as those with fewer than 200 or more than 5,000 detected genes, or with mitochondrial gene proportions greater than 10%. Potential doublets were removed, and only high‐quality cells were retained for further analysis.

### 4.17 Batch Effect Correction and Cell Clustering

Batch effects among samples were corrected using the Harmony algorithm. Gene expression matrices were normalized to total cellular read counts using the LogNormalize method with a scale factor of 10,000, followed by log transformation. Highly variable genes (*n* = 2,000) were identified using the vst method in Seurat and were used for principal component analysis (PCA). Batch correction was performed on the PCA embeddings using the RunHarmony function. The first 13 harmonized principal components were used for UMAP dimensionality reduction. A shared nearest neighbor (SNN) graph was constructed using the FindNeighbors function, and unsupervised clustering was performed with the FindClusters function at a resolution of 0.5. Differentially expressed genes (DEGs) between CD66c^+^ and CD66c^−^ BC populations were identified using the Wilcoxon signed‐rank test. Genes with an adjusted *p*‐value < 0.05, a minimum expression fraction (min.pct) > 0.25, and an absolute log_2_ fold change > 0.25 were considered significant. Gene Ontology enrichment analysis was performed on DEGs using the clusterProfiler package (v4.6.0).

### 4.18 Trajectory Inference

Developmental trajectories were inferred using Monocle v2.18.0. Gene expression matrices and raw counts were extracted from Seurat objects and imported into Monocle. The top 1,000 highly variable genes were selected to order cells in pseudotime. Dimensionality reduction was performed using the “DDRTree” method, and trajectories were visualized with the plot_cell_trajectory function. Branch‐specific regulatory genes were identified using BEAM (Branched Expression Analysis Modeling). Dynamic expression changes of selected genes along pseudotime were visualized using the plot_genes_in_pseudotime function. Monocle2 (v2.18.0) was used to enable branch‐dependent trajectory analyses.

### 4.19 Integration of Public Data

Single‐cell transcriptomes of healthy lungs, IPF lungs, and AT2‐derived organoids were obtained from the NCBI Gene Expression Omnibus (GEO) (accession numbers GSE135893 and GSE132771) and processed using the Seurat pipeline (v4.3.0). Approximately 8,000–10,000 cells per sample were retained after quality control filtering (removal of cells with fewer than 200 or more than 5,000 detected genes, or >10% mitochondrial gene content). Epithelial populations were selected by excluding mesenchymal cells, and then integrated with our in‐house single‐cell datasets from distal lung tissues, DLOs, and organoids derived from AT2, secretory, or BCs. Integration anchors were identified from the first 20 principal components (PCs), and datasets were merged using the IntegrateData function in Seurat with default parameters. Batch effects were further corrected using the Harmony package (v1.2). The integrated objects were annotated by cell type and used to assess epithelial subtype composition across samples. Significantly enriched gene sets were identified using Gene Ontology enrichment analysis.

BCs from distal lung tissues, DLOs, and FACS‐sorted organoid samples were additionally integrated with BC libraries from two IPF cases reported by Wang et al. (NCBI SRA: SRR22542111 and SRR22542112). CD66c^+^ BCs were annotated based on marker genes defining the “cluster B” subpopulation reported by Wang et al., and comparative analyses were performed to quantify the proportion of CD66c^+^ BCs across different samples. All public datasets were downloaded from open‐access repositories in compliance with the relevant data use policies.

### 4.20 Processing of Visium Spatial Transcriptomic Data

Visium spatial transcriptomic data were performed and analyzed in R (v4.2.3) using Seurat (v4.4.0) and semla (v1.3.2). For the human IPF sample, raw Visium data were imported using the Load10X_Spatial function in Seurat. Quality control was performed according to the filtering criteria described in the original publication [31]. Briefly, only tissue‐covered spots were retained. Spots were required to contain at least 350 unique molecular identifiers (UMIs) and express a minimum of 10 detected genes. Genes detected in fewer than five spots or with fewer than 100 total UMIs across the dataset were excluded. Spots with high mitochondrial or hemoglobin gene content (>30% of total UMIs) were removed. Data were subsequently normalized and scaled using Seurat's NormalizeData and ScaleData functions. Histopathological annotations provided in the original study were used to define disease‐associated regions and preserved alveolar regions and visualized using SpatialDimPlot. To assess the spatial distribution of BCs, BC density estimates inferred by cell2location in the original publication were directly adopted without re‐estimation [31]. Spatial expression patterns of marker genes associated with distinct BC states and key signaling pathways were visualized using Seurat's SpatialFeaturePlot function. For pathway‐level analysis, signaling pathway activity scores were computed using PaaSc (Pathway Activity Analysis for Single Cells) [52]. Multiple correspondence analysis (MCA) was first performed using the computeMCA function to project genes and spots into a shared low‐dimensional space. Gene occurrence rates for each target pathway gene set were calculated using getGeneRate. KEGG signaling pathway gene sets from the MSigDB database were used as background gene sets. Linear regression analysis was conducted with the doRegression function to identify dimensions associated with specific pathways. Pathway activity scores were subsequently calculated using the computeScore function with default parameters and standardized as z‐scores for downstream visualization.

### 4.21 Quantification and Statistical Analysis

All statistical analyses were performed using GraphPad Prism 9 (GraphPad Software, USA). Data are presented as mean ± standard error of the mean (SEM) unless otherwise specified. Comparisons between two groups were performed using an unpaired two‐tailed Student's *t*‐test. For analyses involving three or more groups, one‐way ANOVA followed by an appropriate multiple comparison test was used. When equal variances were not assumed, Welch's one‐way ANOVA followed by Dunnett T3 multiple comparisons test was applied. For data that did not meet normality assumptions, nonparametric tests were used. Statistical significance was defined as *p* < 0.05 and indicated as follows: *p* < 0.05 (*), *p* < 0.01 (**), *p* < 0.001 (***), *p* < 0.0001 (****). Sample sizes (*n*) indicated in the Figure legends represent biological replicates. No data points were excluded unless otherwise stated. All experiments were independently repeated at least three times, unless otherwise specified.

## Author Contributions

Z.S., X.L., S.S., N.J., and K.L. conceived the project and supervised the research. K.L. and H.W. performed the in vitro and in vivo experiments, analyzed the data, and wrote the original draft. X.D. conducted bioinformatics analyses with assistance from Y.J. and Y.W. J.S. and H.D. were responsible for tissue processing. M.Y. contributed to the acquisition and clinical coordination of human lung tissue samples. X.W. and L.Y. contributed to organoid experiments. Z.S., X.L., S.S., and N.J. provided overall supervision and revised the manuscript.

## Conflicts of Interest

The authors declare no conflict of interest.

## Code Availability Statement

Custom scripts used in this study are available from the corresponding author upon reasonable request.

## Supporting information




**Supporting File 1**: advs75185‐sup‐0001‐SuppMat.pdf.

**Supporting File 2**: advs75185‐sup‐0002‐TableS1‐S2.docx.

## Data Availability

The raw sequencing data generated in this study have been deposited in the Genome Sequence Archive (GSA) at the National Genomics Data Center, China National Center for Bioinformation / Beijing Institute of Genomics, Chinese Academy of Sciences, under accession number HRA009633 (https://ngdc.cncb.ac.cn/gsa‐human). Previously published scRNA‐seq datasets re‐analyzed in this study are available in the NCBI GEO and SRA databases under the accession numbers GSE150068 and GSE150247 (GEO), and SRR22542111 and SRR22542112 (SRA). All other data supporting the findings of this study are available from the corresponding author upon reasonable request.
