# Supplementary material for: Activation of ERBB4 Pathway Inhibits Pathological Transdifferentiation of Lung Epithelial Progenitors into CD66c+ Basal Cells in Severe Lung Injury
Source: Adv Sci (Weinh). 2026 Apr 7:e19151. Online ahead of print. doi: 10.1002/advs.202519151 (PMC13334660; doi:10.1002/advs.202519151)
Supplement: Supplementary file 1 — Supporting File 1: advs75185‐sup‐0001‐SuppMat.pdf. [file ADVS-9999-e19151-s002.pdf]

Supplementary Information

for

Activation of ERBB4 Pathway Inhibits Pathological Transdifferentiation of Lung Epithelial Progenitors into CD66c⁺ Basal Cells in Severe Lung Injury

Kaijun Lin[1,2,6], Xinran Deng[1,6], Haonan Wang[1,2,6], Yamei Jiang[1,2], Jian Sun[3], Hailin Ding[3], Ming Ye[5], Xiaoting Wang[1,2], Yu Wang[1], Li Yuan[3], Zhenju Song[3,*], Xinhua Lin[1,2,4,*], Shenfei Sun[1,2,4,*], and Ning Jiang[1,7*]

[6] These authors contributed equally: Kaijun Lin, Xinran Deng, Haonan Wang.

*Correspondence to: Xinhua Lin (xlin@fudan.edu.cn); Zhenju Song (song.zhenju@zs-hospital.sh.cn); Shenfei Sun (shenfei_sun@fudan.edu.cn); or Ning Jiang (ningjiang@fudan.edu.cn).

This PDF file includes:

Supplementary Figures S1–S23

Supplementary Figure Legends

Supplementary References

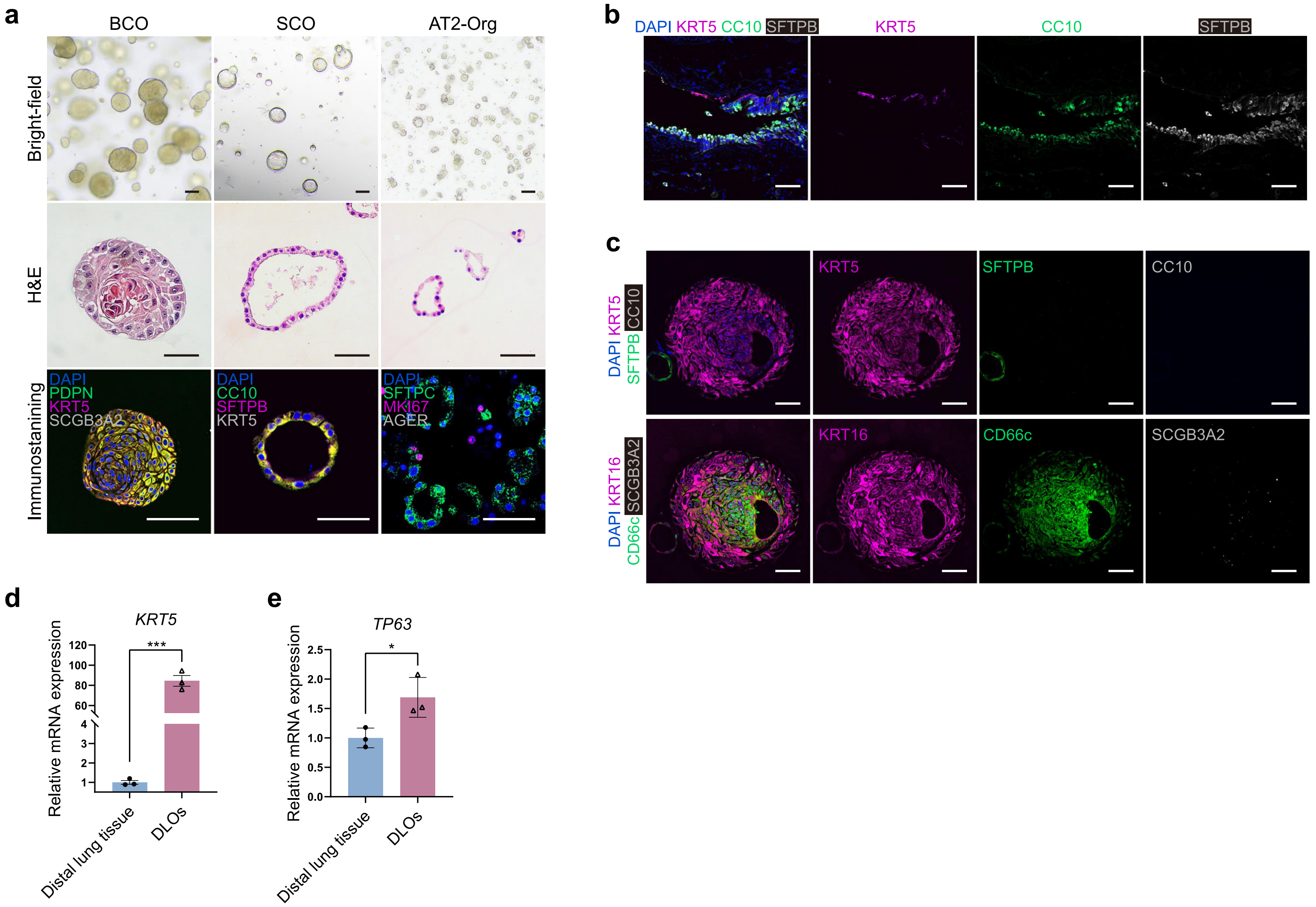

Figure S1. Morphological and lineage characterization of distinct human distal lung organoid subtypes. (a) Representative images of three morphologically distinct human distal lung organoid (DLO) subtypes generated from lineage-sorted epithelial populations cultured under DLO conditions: basal cell organoids (BCOs; dense spherical structures), secretory cell organoids (SCOs; organized luminal architecture), and alveolar type 2 organoids (AT2-Orgs; ~50 μm in diameter). Shown are bright-field images, H&E staining, and immunofluorescence staining for lineage markers. Scale bars: 50 μm.

(b) Representative immunofluorescence images of human distal lung sections stained for KRT5 (magenta), CC10 (green), SFTPB (gray), and nuclei (DAPI, blue). Scale bar: 50 μm.

(c) Immunofluorescence staining of serial DLO sections showing expression of KRT5, SFTPB, CC10, KRT16, CD66c, and SCGB3A2. Scale bars: 50 μm.

(d, e) Relative mRNA expression of *KRT5* (d) and *TP63* (e) in distal lung tissue versus DLOs. Data are presented as mean ± SEM (n = 3; unpaired two-tailed Student's t-test). *$p < 0.05$, ***$p < 0.001$.

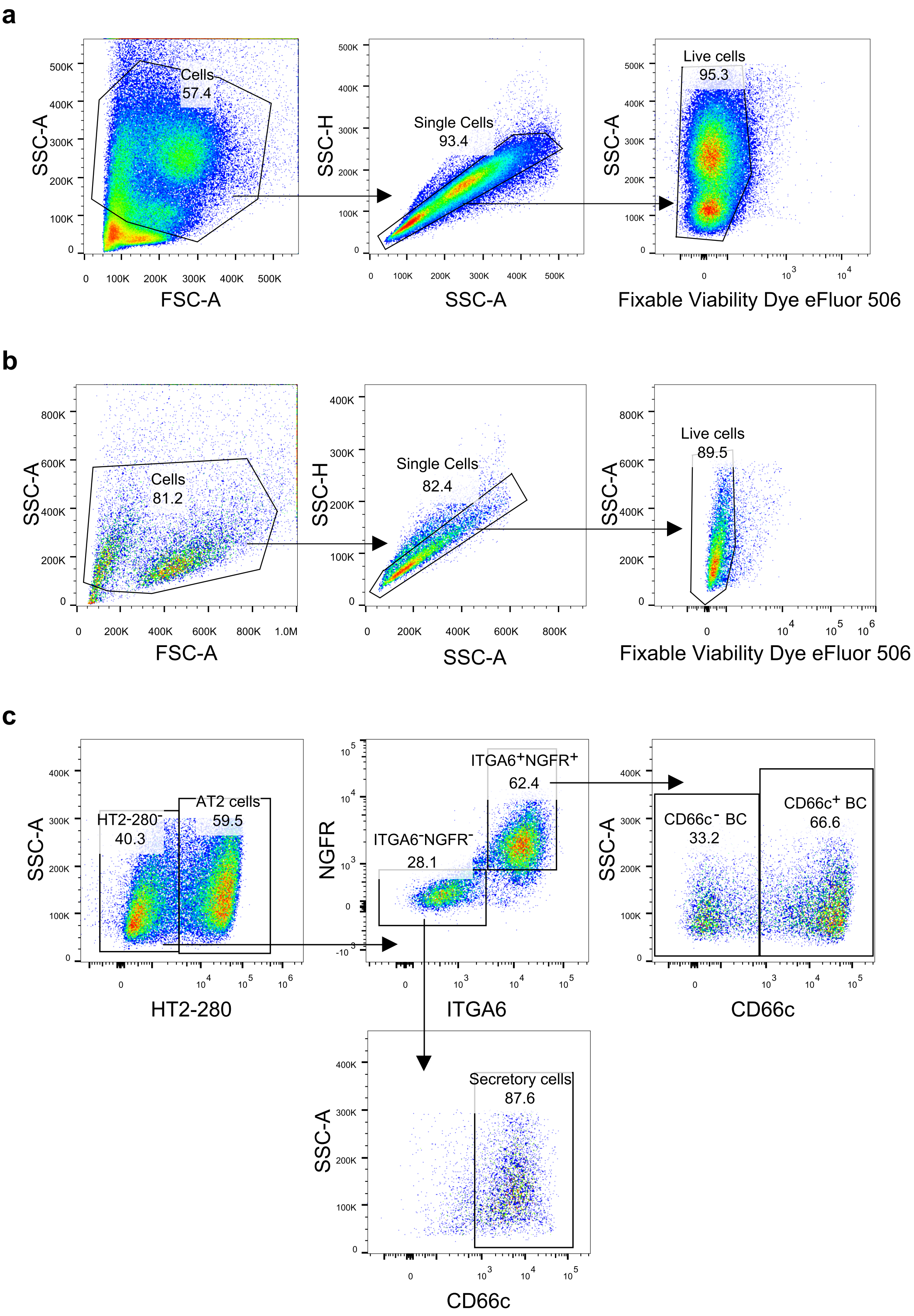

Figure S2. Flow cytometry gating strategy for epithelial cell identification and sorting in human distal lung tissue and organoids.

(a, b) Flow cytometry gating strategy for identification of single, viable epithelial cells from freshly isolated human distal lung tissue (a) and cultured DLOs (b).

(c) Flow cytometry gating and sorting strategy for epithelial subsets within human DLOs, including AT2 cells (HT2-280^{+} ITGA6^{-} NGFR^{-}), secretory cells (HT2-280^{-} ITGA6^{-} NGFR^{-} CD66c^{+}), CD66c^{-} basal cells (HT2-280^{-} ITGA6^{+} NGFR^{+} CD66c^{-}), and CD66c^{+} basal cells (HT2-280^{-} ITGA6^{+} NGFR^{+} CD66c^{+}).

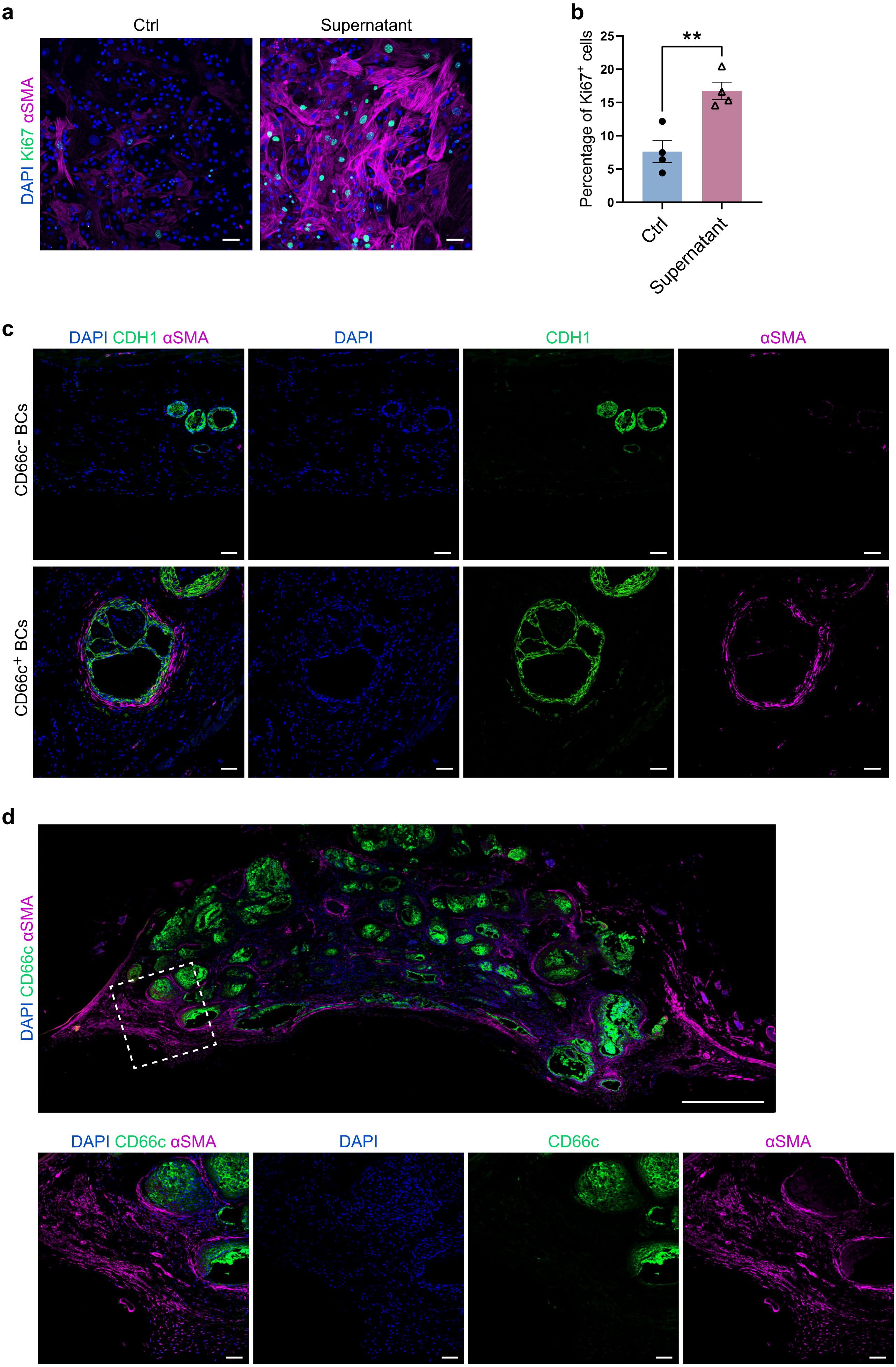
a
Ctrl
Supernatant
DAPI Ki67 αSMA
b
Percentage of Ki67+ cells
25
20
15
10
5
0
**
Ctrl
Supernatant
c
DAPI CDH1 αSMA
DAPI
CDH1
αSMA
CD66c⁻ BCs
CD66c+ BCs
d
DAPI CD66c αSMA
DAPI CD66c αSMA
DAPI
CD66c
αSMA

Figure S3. CD66c$^+$ basal cells promote fibroblast activation and myofibroblast accumulation in vitro and in vivo.

(a) Immunofluorescence staining of MRC-5 fibroblasts cultured under control conditions or treated with conditioned medium from CD66c$^+$ basal cells (BCs). Cells were stained for Ki67 (green), αSMA (magenta), and DAPI (blue). Scale bars, 50 µm.

(b) Quantification of Ki67$^+$ MRC-5 fibroblasts shown in (a). Data are presented as mean ± SEM (n = 4; unpaired two-tailed Student's t-test). $^{**}p < 0.01$.

(c) Representative immunofluorescence images of subcutaneous grafts in NSG mice 18 days after transplantation of FACS-sorted CD66c$^-$ or CD66c$^+$ BCs. Sections were stained for CDH1 (green), αSMA (magenta), and DAPI (blue). Scale bars, 50 µm.

(d) Multiplex immunofluorescence staining of CD66c$^+$ BC–derived grafts showing CD66c (green), αSMA (magenta), and DAPI (blue). Upper, low-magnification overview; lower, higher-magnification views of the boxed region shown as merged and single-channel images. Scale bars, 1000 µm (upper) and 50 µm (lower).

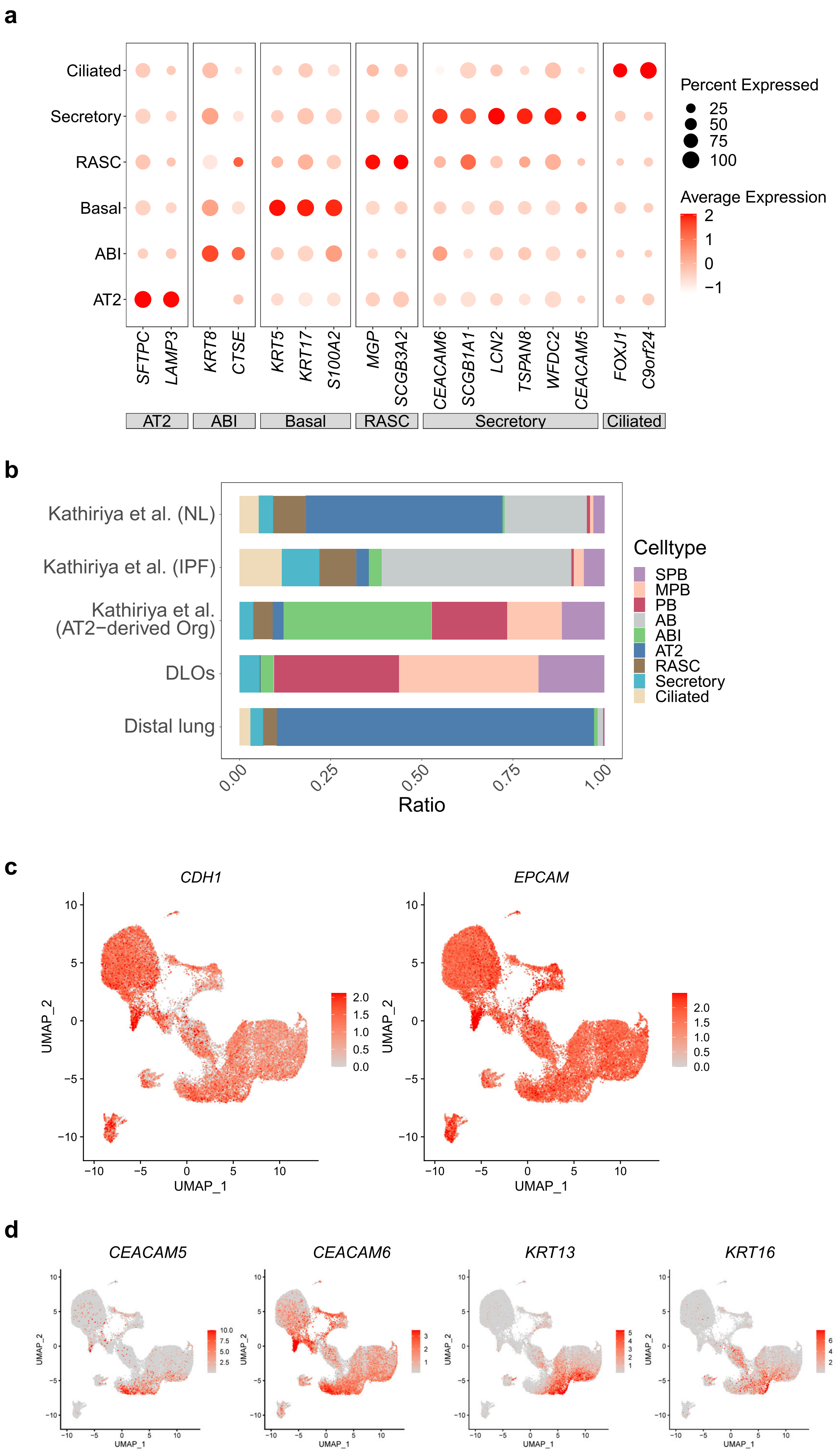
a
Ciliated
Secretory
RASC
Basal
ABI
AT2
SFTPC
LAMP3
KRT8
CTSE
KRT5
KRT17
S100A2
MGP
SCGB3A2
CEACAM6
SCGB1A1
LCN2
TSPAN8
WFDC2
CEACAM5
FOXJ1
C9orf24
AT2
ABI
Basal
RASC
Secretory
Ciliated
Percent Expressed
25
50
75
100
Average Expression
2
1
0
−1
b
Kathiriya et al. (NL)
Kathiriya et al. (IPF)
Kathiriya et al. (AT2−derived Org)
DLOs
Distal lung
0.00
0.25
0.50
0.75
1.00
Ratio
Celltype
SPB
MPB
PB
AB
ABI
AT2
RASC
Secretory
Ciliated
c
CDH1
EPCAM
UMAP_1
UMAP_2
d
CEACAM5
CEACAM6
KRT13
KRT16
UMAP_1
UMAP_2

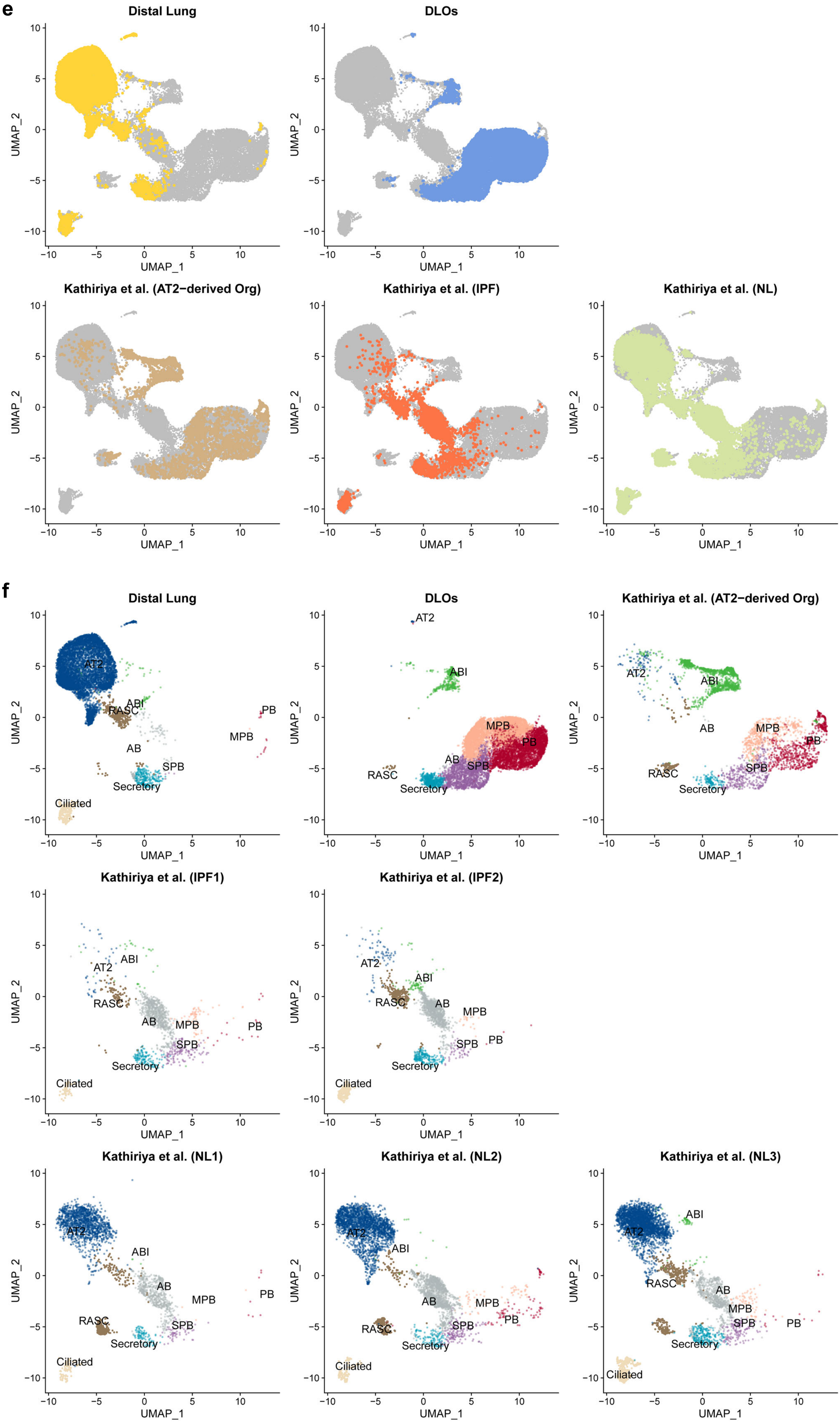

e
Distal Lung
DLOs
Kathiriya et al. (AT2−derived Org)
Kathiriya et al. (IPF)
Kathiriya et al. (NL)
UMAP_1
UMAP_2
f
Distal Lung
DLOs
Kathiriya et al. (AT2−derived Org)
Kathiriya et al. (IPF1)
Kathiriya et al. (IPF2)
Kathiriya et al. (NL1)
Kathiriya et al. (NL2)
Kathiriya et al. (NL3)
AT2
ABI
RASC
AB
MPB
PB
SPB
Secretory
Ciliated
UMAP_1
UMAP_2

Figure S4. Integrated single-cell analysis reveals conserved epithelial states and enrichment of secretory-primed basal cells across distal lung organoids and IPF lungs.

All panels are derived from a single integrated scRNA-seq dataset comprising epithelial cells from the indicated sources.

(a) Dot plot showing representative marker gene expression defining major epithelial cell populations in the integrated scRNA-seq dataset, comprising epithelial cells from uncultured distal lung tissue, day-14 distal lung organoids (DLOs), AT2-derived organoids, and normal or IPF human lungs (Kathiriya et al., 2022). Cell-type annotations include alveolar type 2 cells (AT2), alveolar–basal intermediates (ABI), activated basal cells (AB), secretory-primed basal cells (SPB), multipotent basal cells (MPB), proliferating basal cells (PB), respiratory airway secretory cells (RASC), secretory cells, and ciliated cells.
(b) Stacked bar plots showing the relative proportions of epithelial cell subsets across distal lung tissue, DLOs, and published datasets, highlighting the enrichment of SPBs in DLOs and IPF samples.
(c) UMAP plots of the integrated epithelial dataset showing broad epithelial identity, as indicated by expression of the pan-epithelial markers *CDH1* and *EPCAM*.
(d) UMAP feature plots showing the distribution of secretory-primed basal cell marker genes (*CEACAM5*, *CEACAM6*, *KRT13*, and *KRT16*) across the integrated dataset, demonstrating conserved localization of SPBs in DLOs and IPF lung samples.
(e) UMAP embeddings of the integrated scRNA-seq dataset colored by sample origin.
(f) Sample-resolved UMAP projections illustrating epithelial cell-type composition and heterogeneity across individual datasets included in the integrated analysis.

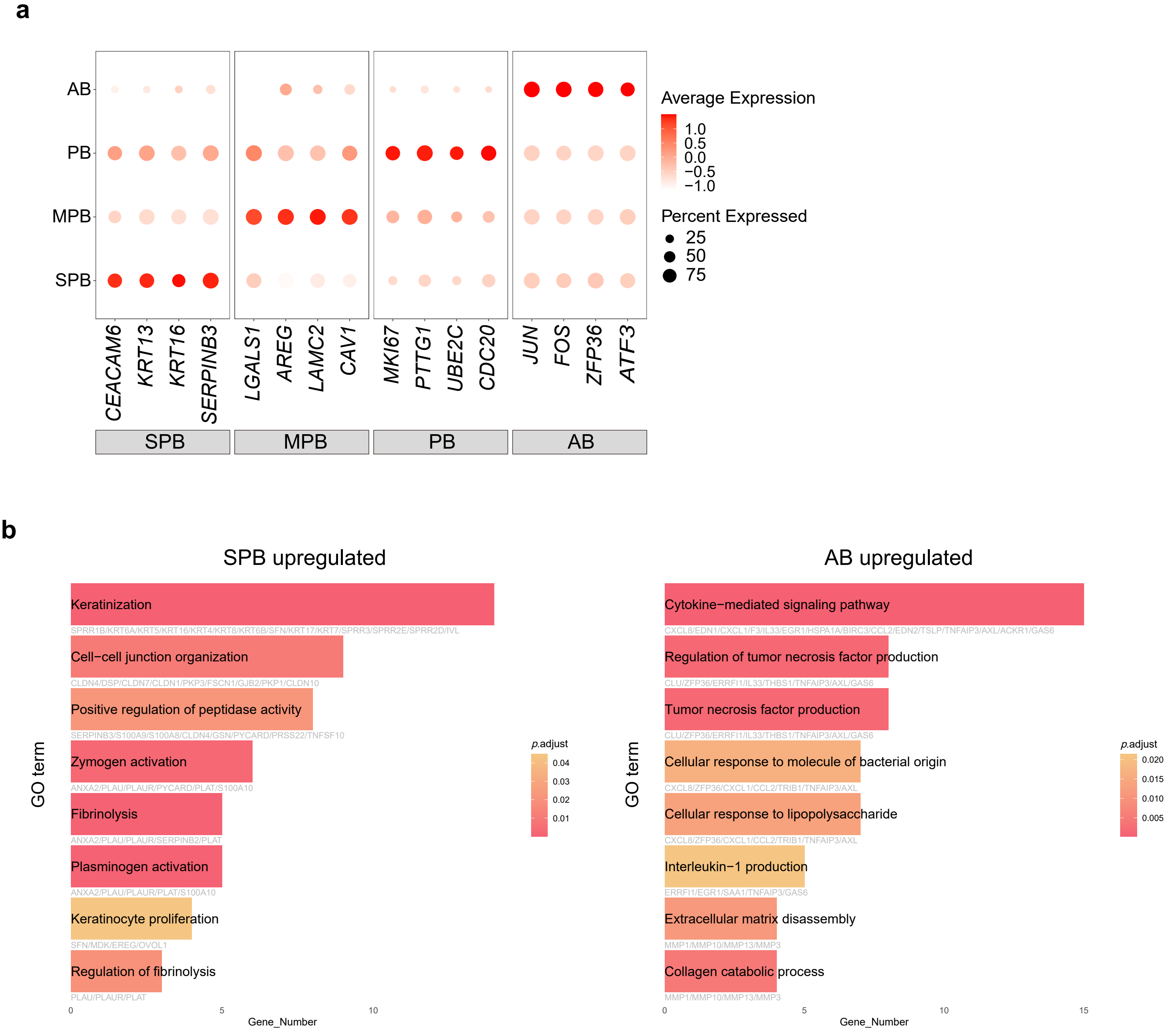

Figure S5. Transcriptional features and functional programs of basal cell subtypes derived from an integrated epithelial scRNA-seq dataset.

All panels are derived from a single integrated scRNA-seq dataset comprising epithelial cells from the indicated sources.

(a) Dot plot showing marker gene expression distinguishing four conserved basal cell (BC) subtypes—secretory-primed basal cells (SPBs), multipotent basal cells (MPBs), proliferating basal cells (PBs), and activated basal cells (ABs)—identified from basal cells extracted from the integrated scRNA-seq dataset described in Figure S4. Dot size indicates the fraction of expressing cells, and color denotes average expression. (b) Gene Ontology (GO) biological process enrichment analysis of genes differentially expressed between SPBs and ABs. Left: pathways enriched in SPBs; right: pathways enriched in ABs.

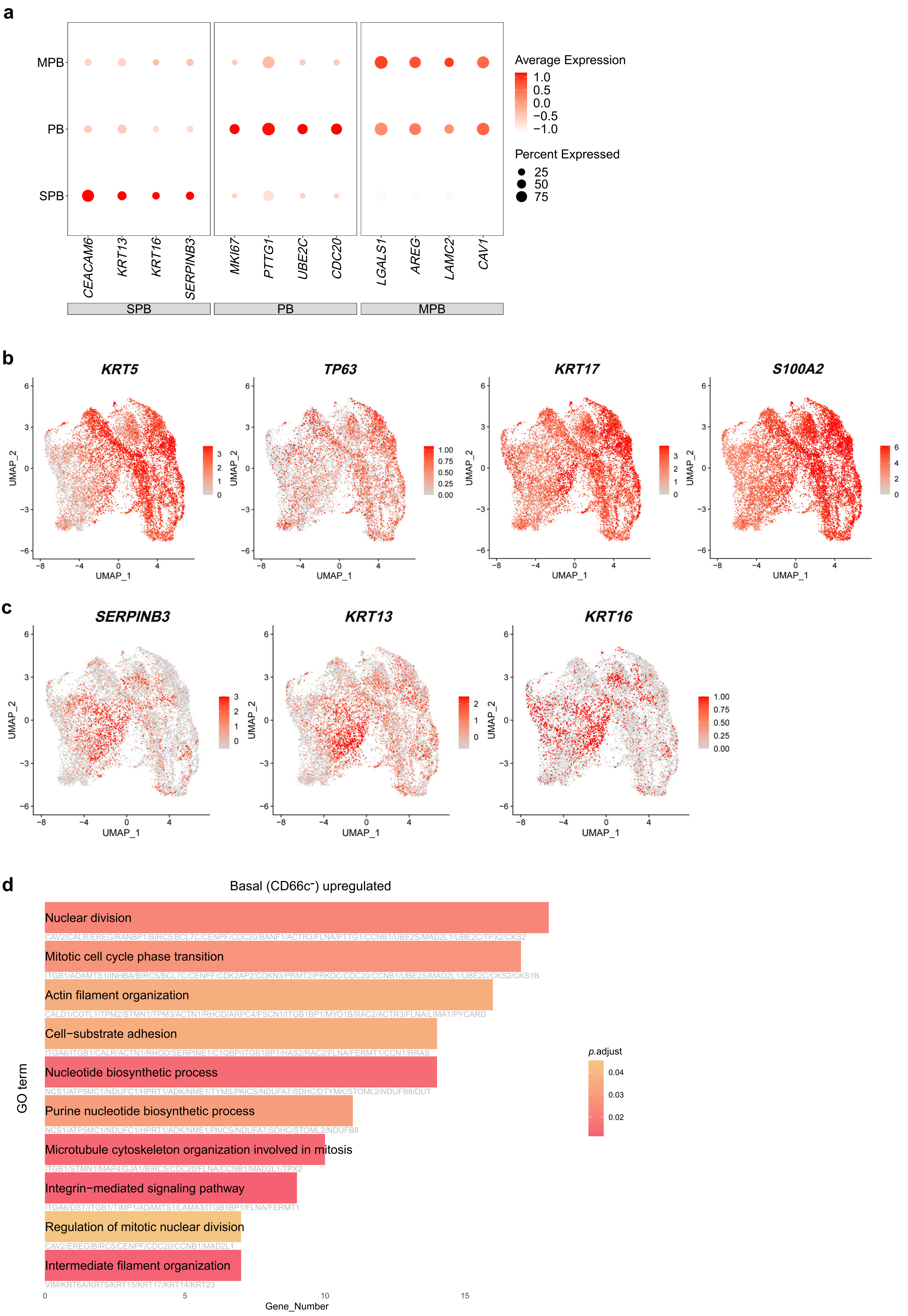
a
MPB
PB
SPB
CEACAM6
KRT13
KRT16
SERPINB3
MKI67
PTTG1
UBE2C
CDC20
LGALS1
AREG
LAMC2
CAV1
SPB
PB
MPB
Average Expression
1.0
0.5
0.0
−0.5
−1.0
Percent Expressed
25
50
75
b
KRT5
TP63
KRT17
S100A2
UMAP_1
UMAP_2
c
SERPINB3
KRT13
KRT16
UMAP_1
UMAP_2
d
Basal (CD66c⁻) upregulated
Nuclear division
CAV2/CALR/EREG/RANBP1/BIRC5/BCL7C/CENPF/CDC20/BANF1/ACTR3/FLNA/PTTG1/CCNB1/UBE2S/MAD2L1/UBE2C/TPX2/CKS2
Mitotic cell cycle phase transition
ITGB1/ADAMTS1/INHBA/BIRC5/BCL7C/CENPF/CDK2AP2/CDKN3/PRMT2/PRKDC/CDC20/CCNB1/UBE2S/MAD2L1/UBE2C/CKS2/CKS1B
Actin filament organization
CALD1/COTL1/TPM2/STMN1/TPM3/ACTN1/RHOD/ARPC4/FSCN1/ITGB1BP1/MYO1B/RAC2/ACTR3/FLNA/LIMA1/PYCARD
Cell−substrate adhesion
ITGA6/ITGB1/CALR/ACTN1/RHOD/SERPINE1/C1QBP/ITGB1BP1/HAS2/RAC2/FLNA/FERMT1/CCN1/RRAS
Nucleotide biosynthetic process
NCS1/ATP5MC1/NDUFC1/HPRT1/ADK/NME1/TYMS/PAICS/NDUFA7/SDHC/DTYMK/STOML2/NDUFB8/DUT
Purine nucleotide biosynthetic process
NCS1/ATP5MC1/NDUFC1/HPRT1/ADK/NME1/PAICS/NDUFA7/SDHC/STOML2/NDUFB8
Microtubule cytoskeleton organization involved in mitosis
ITGB1/STMN1/MAP4/GJA1/BIRC5/CDC20/FLNA/CCNB1/MAD2L1/TPX2
Integrin−mediated signaling pathway
ITGA6/DST/ITGB1/TIMP1/ADAMTS1/LAMA3/ITGB1BP1/FLNA/FERMT1
Regulation of mitotic nuclear division
CAV2/EREG/BIRC5/CENPF/CDC20/CCNB1/MAD2L1
Intermediate filament organization
VIM/KRT6A/KRT5/KRT15/KRT17/KRT14/KRT23
GO term
Gene_Number
p.adjust
0.04
0.03
0.02

Figure S6. Single-cell characterization of basal cell states in FACS-sorted CD66c^{+} and CD66c^{-} basal cells.

All panels are derived from a single integrated scRNA-seq dataset comprising epithelial cells from the indicated sources.

(a) Dot plot showing representative marker gene expression defining basal cell (BC) subtypes identified from the integrated scRNA-seq dataset of FACS-sorted CD66c^{+} and CD66c^{-} basal cells, including secretory-primed basal cells (SPBs), proliferating basal cells (PBs), and multipotent basal cells (MPBs). Dot size indicates the percentage of expressing cells and color indicates average expression.
(b) Feature plots showing expression of canonical BC markers (*KRT5*, *TP63*, *KRT17*, and *S100A2*) across the integrated UMAP embedding of CD66c^{+} and CD66c^{-} BCs.
(c) Feature plots showing expression of SPB-associated genes (*SERPINB3*, *KRT13*, and *KRT16*) across the same integrated dataset.
(d) GO enrichment analysis of genes upregulated in FACS-sorted CD66c^{-} BCs, highlighting cell cycle and mitosis-related processes.

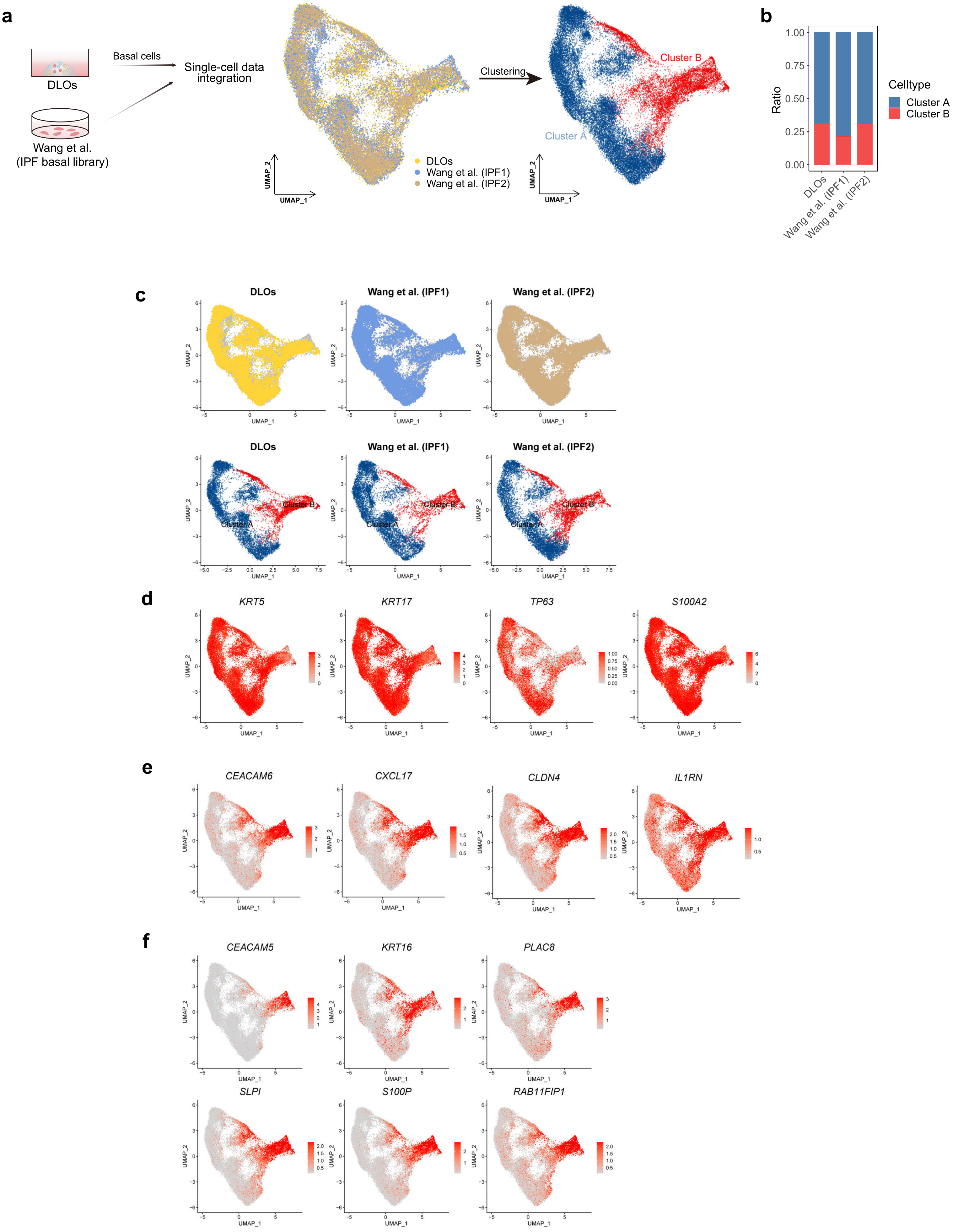
a
Basal cells
DLOs
Wang et al.
(IPF basal library)
Single-cell data integration
DLOs
Wang et al. (IPF1)
Wang et al. (IPF2)
Clustering
Cluster B
Cluster A
UMAP_1
UMAP_2
b
Ratio
1.00
0.75
0.50
0.25
0.00
Celltype
Cluster A
Cluster B
DLOs
Wang et al. (IPF1)
Wang et al. (IPF2)
c
DLOs
Wang et al. (IPF1)
Wang et al. (IPF2)
d
KRT5
KRT17
TP63
S100A2
e
CEACAM6
CXCL17
CLDN4
IL1RN
f
CEACAM5
KRT16
PLAC8
SLPI
S100P
RAB11FIP1

Figure S7. Integration of distal lung organoid–derived basal cells with IPF basal cell transcriptomes reveals conserved profibrotic basal cell states.

(a) Schematic overview of basal cell (BC) isolation from distal lung organoids (DLOs) and integration with published IPF basal cell single-cell RNA-seq datasets (Wang *et al.*, 2022). Integrated UMAP analysis resolves two transcriptionally conserved BC clusters, designated Cluster A and Cluster B.

(b) Relative proportions of Cluster A and Cluster B across DLO-derived BCs and IPF basal cell datasets.

(c) Sample-resolved UMAP projections showing the distribution of integrated BCs derived from DLOs and IPF patient samples (Wang *et al.*, IPF1 and IPF2).

(d) UMAP feature plots showing uniform expression of canonical basal cell markers (*KRT5*, *KRT17*, *TP63*, and *S100A2*) across the integrated BC population, confirming shared basal identity of both clusters.

(e) UMAP feature plots showing genes enriched in Cluster B basal cells, including *CEACAM6*, *CXCL17*, *CLDN4*, and *IL1RN*, across the integrated datasets.

(f) UMAP feature plots showing the expression of secretory-primed basal cell (SPB) marker genes, including *CEACAM5*, *KRT16*, *PLAC8*, *SLPI*, *S100P*, and *RAB11FIP1*, within Cluster B.

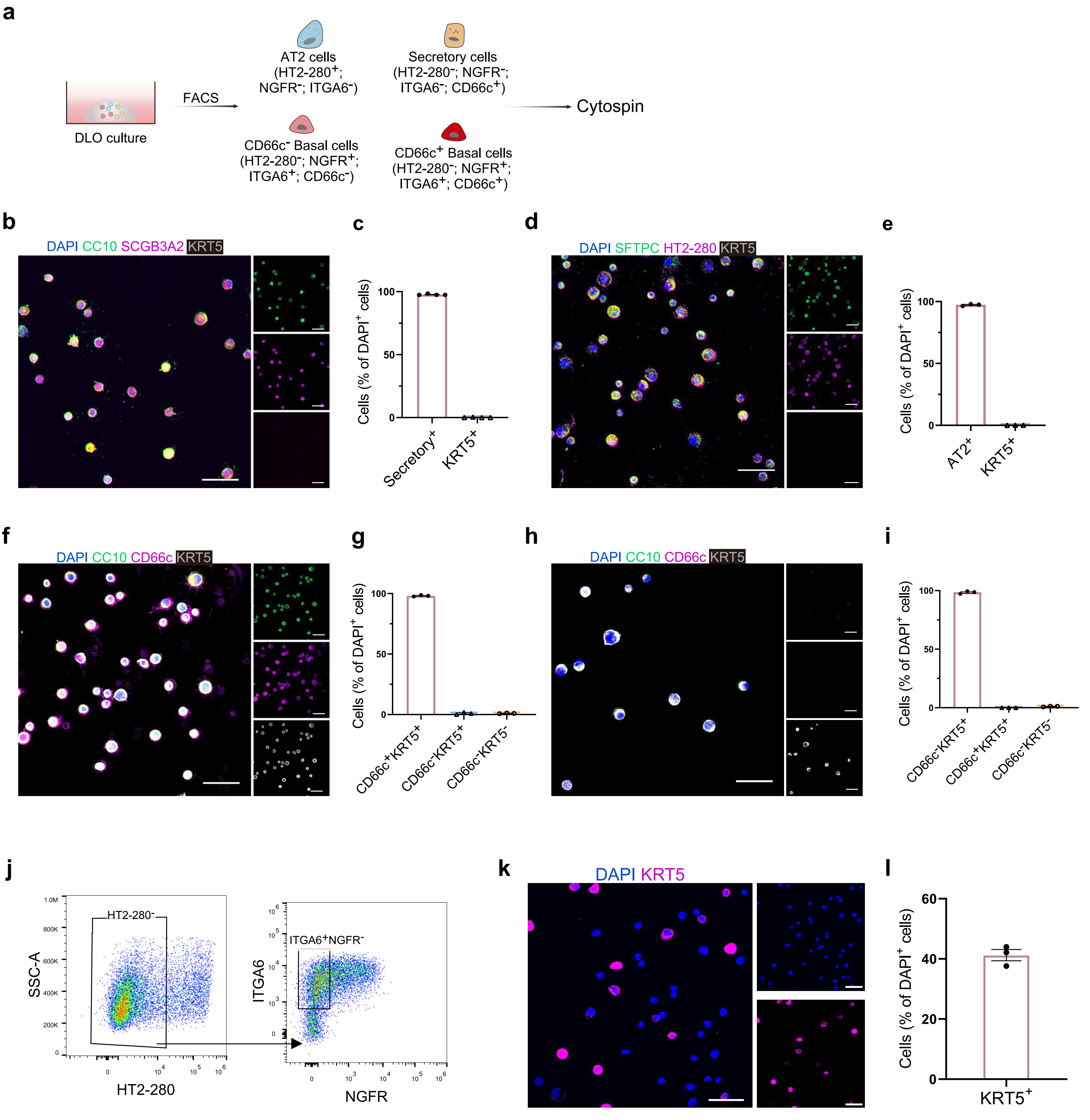

Figure S8. Validation of FACS sorting purity for distal lung epithelial populations by cytospin immunofluorescence.

(a) Schematic overview of the experimental workflow for fluorescence-activated cell sorting (FACS), cytospin-based immunofluorescence validation.

(b) Representative cytospin immunofluorescence images of sorted secretory cells stained for CC10 (green), SCGB3A2 (magenta), and KRT5 (gray). Scale bars, 50 µm.

(c) Quantification of secretory cell purity based on cytospin immunofluorescence. Sorted cells were predominantly CC10^{+}, SCGB3A2^{+}, or CC10^{+} SCGB3A2^{+}, with rare KRT5^{+} cells detected (n = 4 independent sorts).

(d) Representative cytospin immunofluorescence images of sorted AT2 cells showing co-expression of SFTPC (green) and HT2-280 (magenta), with minimal KRT5 (gray) signal. Scale bars, 50 µm.
(e) Quantification of AT2 cell purity. HT2-280^{+} SFTPC^{+} cells predominated, with negligible KRT5^{+} contamination (n = 3 independent sorts).
(f) Representative cytospin immunofluorescence images of CD66c^{+} basal cells isolated from distal lung organoids, stained for CC10 (green), CD66c (magenta), and KRT5 (gray). Scale bars, 50 µm.
(g) Quantification of sorting purity for FACS-isolated CD66c^{+} basal cells based on cytospin immunofluorescence counting, showing enrichment of CD66c^{+} KRT5^{+} cells (n = 3 independent sorts).
(h) Representative cytospin immunofluorescence images of CD66c^{-} basal cells isolated from distal lung organoids. Scale bars, 50 µm.
(i) Quantification of cell composition within the CD66c^{-} basal cell fraction, showing enrichment of CD66c^{-} KRT5^{+} cells with minimal contamination (n = 3 independent sorts).
(j) Representative flow cytometry plots showing the gating strategy used to define the ungated epithelial cell population. Ungated cells were identified as EPCAM^{+} HT2-280^{-} ITGA6^{+} NGFR^{-} cells that were not assigned to canonical AT2, secretory, or basal cell gates.
(k) Cytospin immunofluorescence staining of ungated epithelial cells, showing expression of KRT5 (magenta) and nuclei counterstained with DAPI (blue). Right panels show single-channel views. Scale bars, 50 µm.
(l) Quantification of KRT5^{+} cells among ungated epithelial cells, expressed as the percentage of DAPI^{+} cells (n = 3 independent sorts).
Bars represent mean ± SEM; each dot indicates an independent biological replicate.

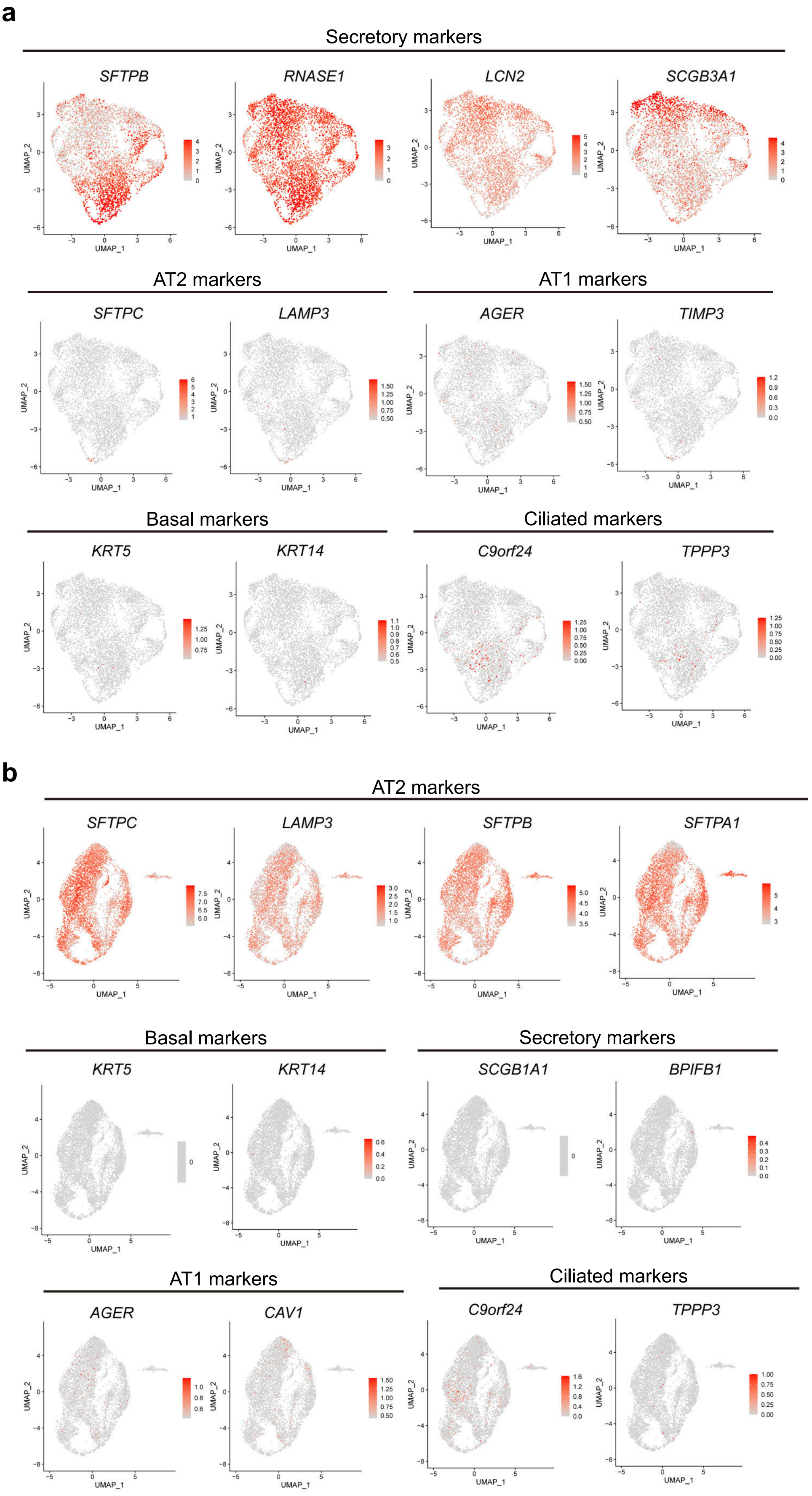

Figure S9. Single-cell transcriptomic validation of secretory and AT2 cell isolation purity.
(a) UMAP visualization of FACS-sorted secretory cells showing robust expression of canonical secretory markers, with minimal expression of markers associated with alternative epithelial lineages.
(b) UMAP visualization of FACS-sorted AT2 cells demonstrating high-purity isolation, characterized by strong expression of AT2 markers and negligible contamination from non-AT2 epithelial populations.

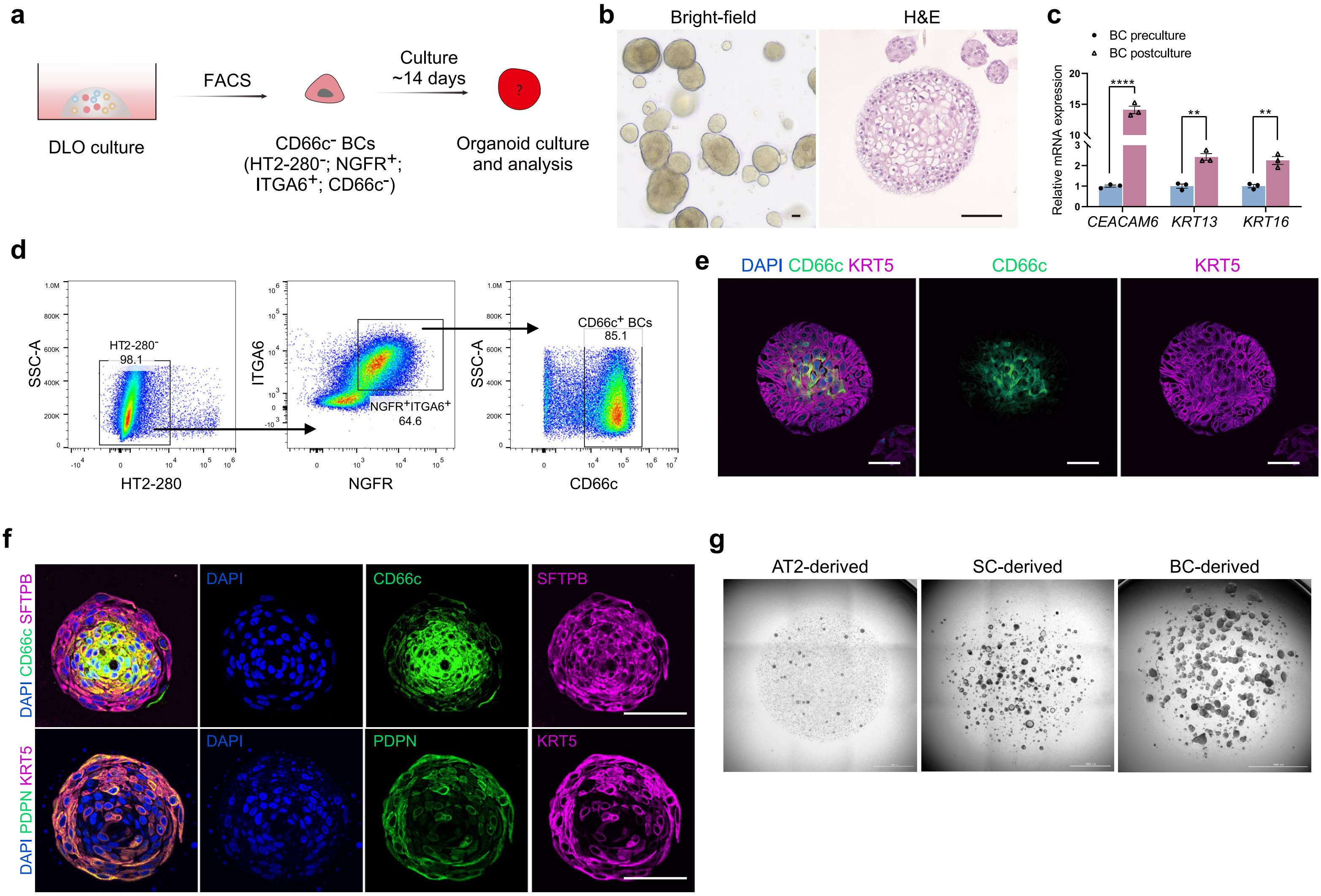

Figure S10. AT2-, secretory-, and CD66c⁻ basal cell–derived organoids exhibit convergent acquisition of CD66c⁺ basal features.

(a) Experimental workflow for FACS-based isolation of CD66c⁻ BCs (HT2-280⁻ NGFR⁺ ITGA6⁺ CD66c⁻) from distal lung organoids (DLOs), followed by organoid culture for ~14 days.

(b) Representative bright-field (left) and H&E-stained (right) images of CD66c⁻ BC-derived organoids after 14 days of culture. Scale bar, 50 μm.

(c) RT–qPCR analysis of CD66c⁻ BCs before and after organoid culture, assessing *CEACAM6* (CD66c), *KRT16*, and *KRT13* expression. Data are presented as mean ± SEM (n = 3; unpaired two-tailed Student's t-test). $^{**}p < 0.01$, $^{****}p < 0.0001$.

(d) Representative flow cytometry plots of single-cell suspensions generated from CD66c⁻ BC-derived organoids.

(e) Immunofluorescence staining of CD66c⁻ BC-derived organoids showing CD66c (green), KRT5 (magenta), and nuclei (DAPI, blue). Scale bar, 50 μm.

(f) Immunofluorescence staining of serial sections from secretory cell–derived organoids for secretory markers (CD66c, SFTPB) and basal cell markers (KRT5, PDPN). Scale bar, 50 μm.

(g) Whole-well bright-field images of organoids derived from AT2 cells, secretory cells, and CD66c⁻ BCs. Scale bar, 2000 μm.

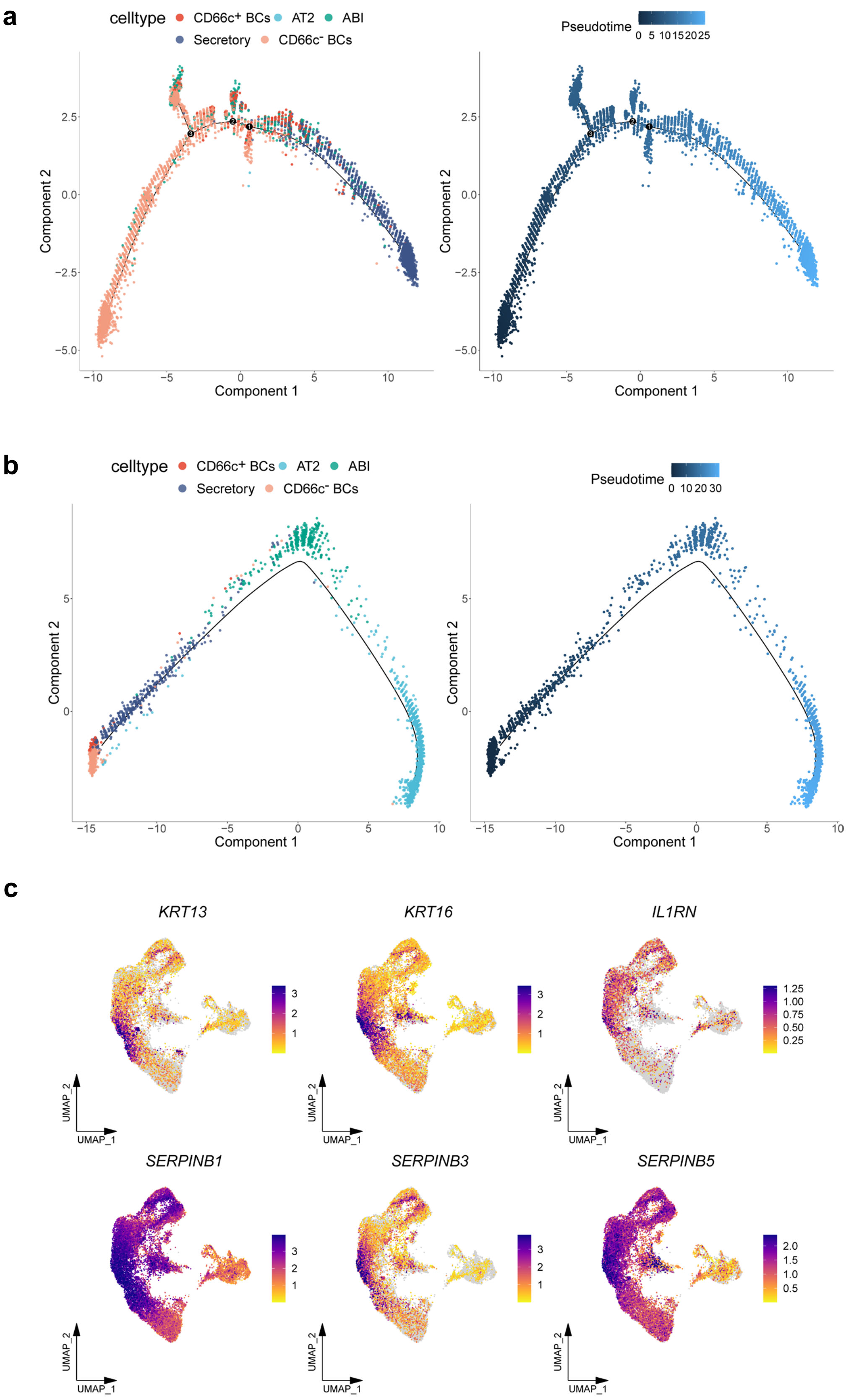

Figure S11. Pseudotemporal trajectories and conserved secretory-primed basal cell programs across distal lung organoid models.

(a) Monocle2 trajectory analysis of CD66c⁻ basal cell (BC)–derived organoids, revealing lineage progression toward CD66c⁺ BC and secretory states. Cells are colored by annotated cell type (left) or pseudotime (right).

(b) Monocle2 trajectory analysis of AT2-derived organoids, including AT2 cells, secretory cells, and basal intermediates, showing lineage relationships among epithelial populations. Left, colored by cell type; right, colored by pseudotime.

(c) UMAP feature plots showing expression of representative secretory-primed basal cell (SPB) markers in an integrated scRNA-seq dataset comprising organoids derived from AT2 cells, secretory cells, and CD66c⁻ BCs (this study), together with AT2-derived organoids from Kathiriya et al., 2022.

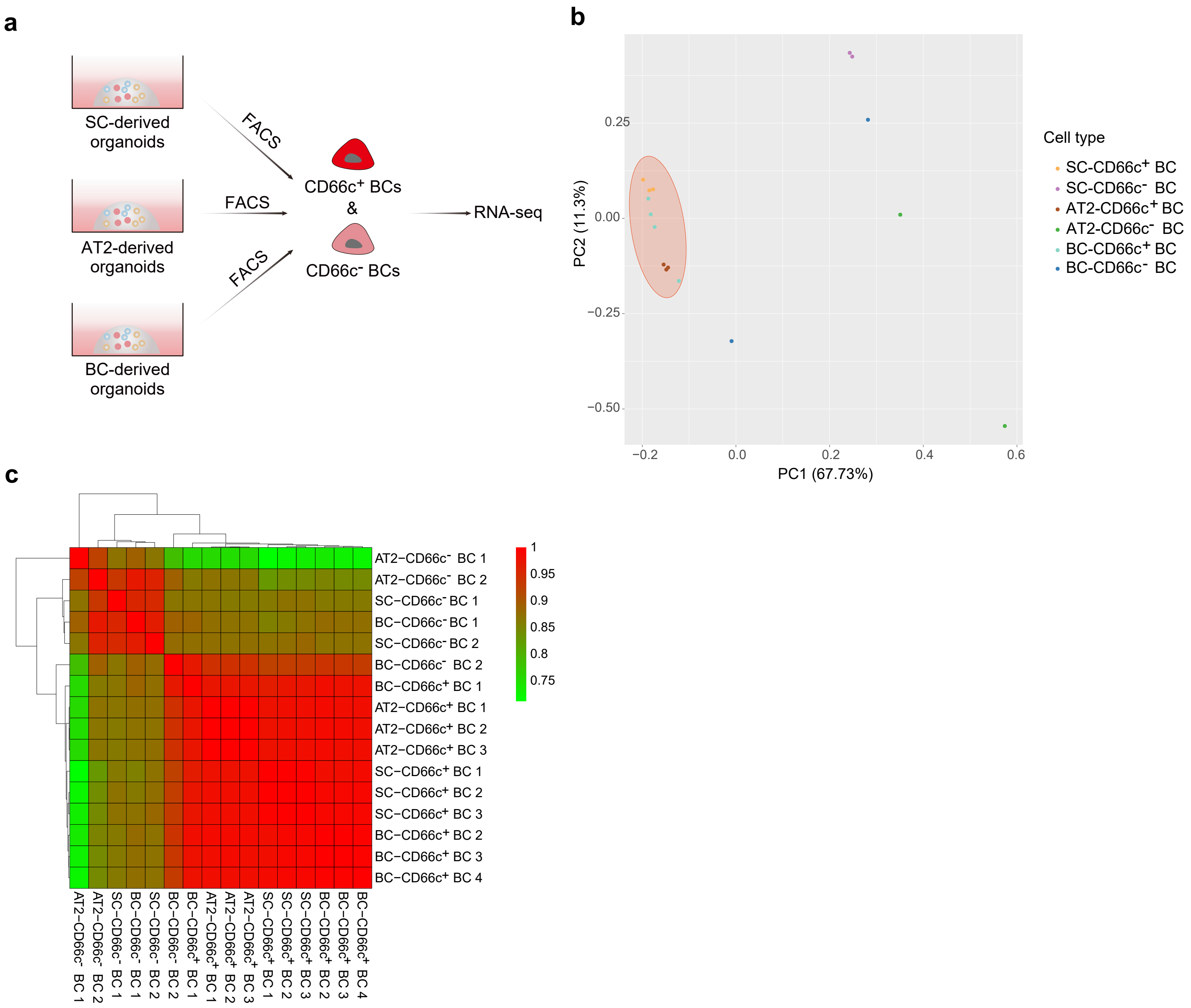

Figure S12. CD66c⁺ basal cells derived from distinct progenitors converge on a shared transcriptional program.

(a) Workflow for FACS-based purification of CD66c$^+$ and CD66c$^-$ BCs from AT2-, secretory-, and BC-derived organoids, followed by bulk RNA sequencing.

(b) Principal component analysis (PCA) of bulk RNA-seq profiles of CD66c$^+$ basal cells derived from distinct progenitor populations, including AT2 cells, secretory cells, and CD66c$^-$ basal cells. CD66c$^+$ basal cells cluster tightly along PC1 (67.73%) and PC2 (11.3%), indicating convergence toward a shared transcriptional state (n = 2-4).

(c) Pearson correlation analysis of bulk RNA-seq data demonstrating highly similar transcriptional profiles among CD66c$^+$ basal cells derived from AT2 cells, secretory cells, and CD66c$^-$ basal cells, irrespective of progenitor origin (n = 2-4).

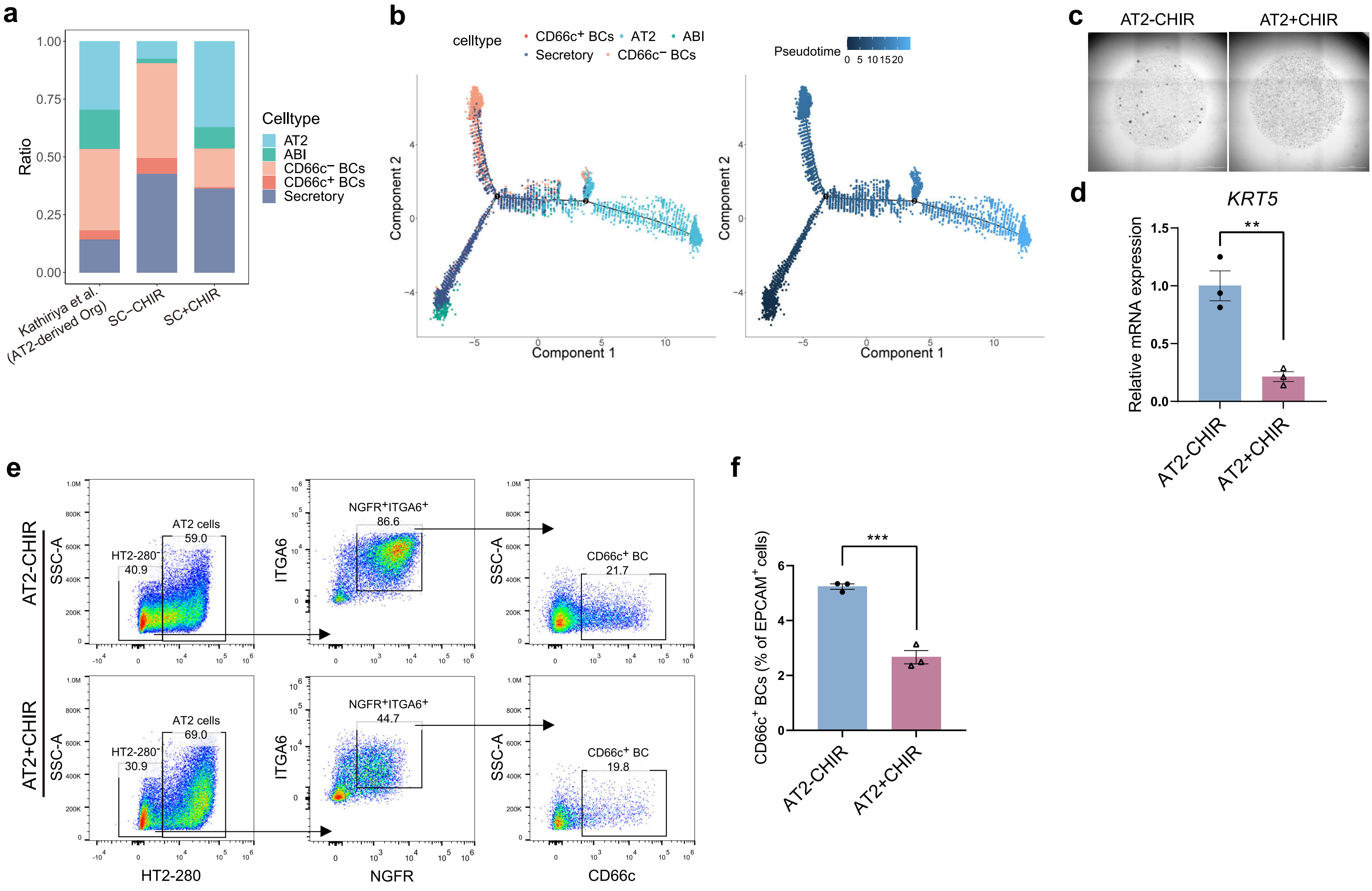

Figure S13. Effect of CHIR99021 treatment on epithelial cell composition and basal cell differentiation in AT2- and secretory cell (SC)-derived organoids.

(a) Stacked bar plots showing proportions of major epithelial populations in AT2-derived organoids (Kathiriya et al., 2022) and SC-derived organoids cultured with or without CHIR99021 (CHIR).

(b) Monocle pseudotime trajectory analysis of SC-derived organoids cultured with CHIR. Cells are colored by cell type (left) or pseudotime (right), showing limited progression toward CD66c^{+} BC–associated states under CHIR treatment.

(c) Bright-field whole-well images of AT2-derived organoids cultured with or without CHIR. Scale bar, 2000 µm.

(d) RT–qPCR analysis of AT2-derived organoids ± CHIR showing relative mRNA expression of the basal marker *KRT5*. Data are presented as mean ± SEM (n = 3; unpaired two-tailed Student's t-test). $^{**}p < 0.01$.

(e) Representative flow cytometry gating strategy for AT2-derived organoids ± CHIR, illustrating reduced proportions of CD66c^{+} BCs upon CHIR treatment.

(f) Flow cytometry–based quantification of CD66c^{+} BC proportions in AT2-derived organoids ± CHIR. Data are presented as mean ± SEM (n = 3; unpaired two-tailed Student's t-test). $^{***}p < 0.001$.

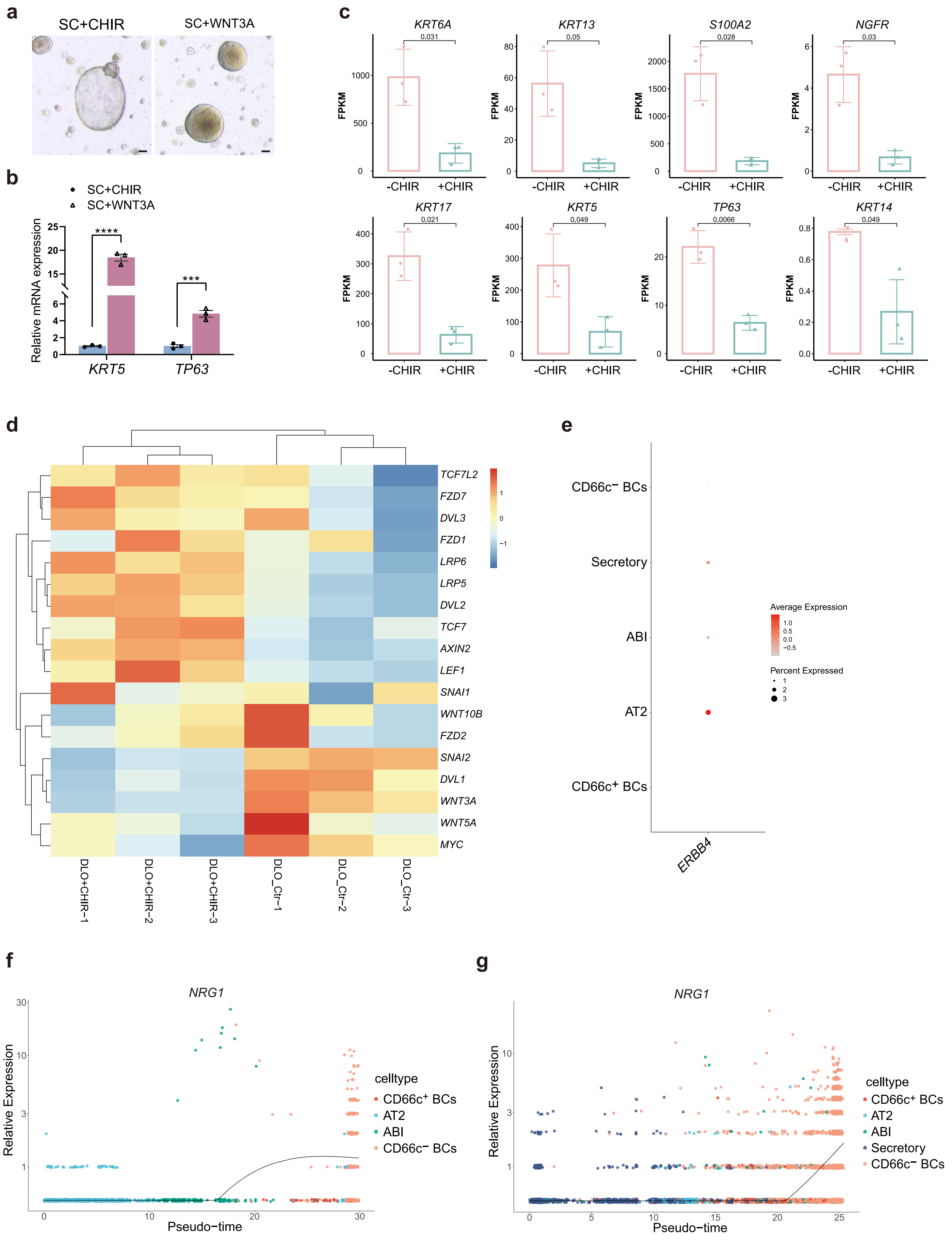
a
SC+CHIR
SC+WNT3A
b
SC+CHIR
SC+WNT3A
Relative mRNA expression
****
***
KRT5
TP63
c
KRT6A
KRT13
S100A2
NGFR
KRT17
KRT5
TP63
KRT14
FPKM
-CHIR
+CHIR
d
TCF7L2
FZD7
DVL3
FZD1
LRP6
LRP5
DVL2
TCF7
AXIN2
LEF1
SNAI1
WNT10B
FZD2
SNAI2
DVL1
WNT3A
WNT5A
MYC
DLO+CHIR−1
DLO+CHIR−2
DLO+CHIR−3
DLO_Ctr−1
DLO_Ctr−2
DLO_Ctr−3
e
CD66c⁻ BCs
Secretory
ABI
AT2
CD66c⁺ BCs
ERBB4
Average Expression
Percent Expressed
f
NRG1
Relative Expression
Pseudo−time
celltype
CD66c⁺ BCs
AT2
ABI
CD66c⁻ BCs
g
NRG1
Relative Expression
Pseudo−time
celltype
CD66c⁺ BCs
AT2
ABI
Secretory
CD66c⁻ BCs

Figure S14. Dissection of CHIR99021-associated transcriptional programs reveals limited overlap with canonical WNT signaling and highlights ERBB4–NRG1–related features during basal lineage progression.

(a) Bright-field images of secretory cell (SC)-derived organoids cultured with CHIR or recombinant WNT3A. Scale bar, 50 µm.

(b) RT–qPCR analysis of SC-derived organoids treated with CHIR or WNT3A, showing relative mRNA expression of *KRT5* and *TP63*. Data are presented as mean ± SEM (n = 3; unpaired two-tailed Student's t-test). ***$p < 0.001$, ****$p < 0.0001$.

(c) Box plots showing FPKM values of canonical basal cell markers in distal lung organoids (DLOs) cultured with or without CHIR. Data are shown as mean ± SD (n = 3). Statistical significance was assessed using an unpaired two-tailed Student's t-test.

(d) Heatmap showing expression patterns of WNT pathway–related genes across DLO samples cultured with or without CHIR treatment. Gene expression values are row-normalized to highlight relative changes between conditions.

(e) Dot plot showing *ERBB4* expression across epithelial cell clusters in integrated scRNA-seq datasets from AT2-derived organoids (Kathiriya *et al.*, 2022) and SC-derived organoids ± CHIR.

(f, g) Monocle pseudotime analysis showing dynamic *NRG1* expression in SCs (f) and AT2 cells (g) along pseudotime trajectories associated with basal cell–directed lineage progression.

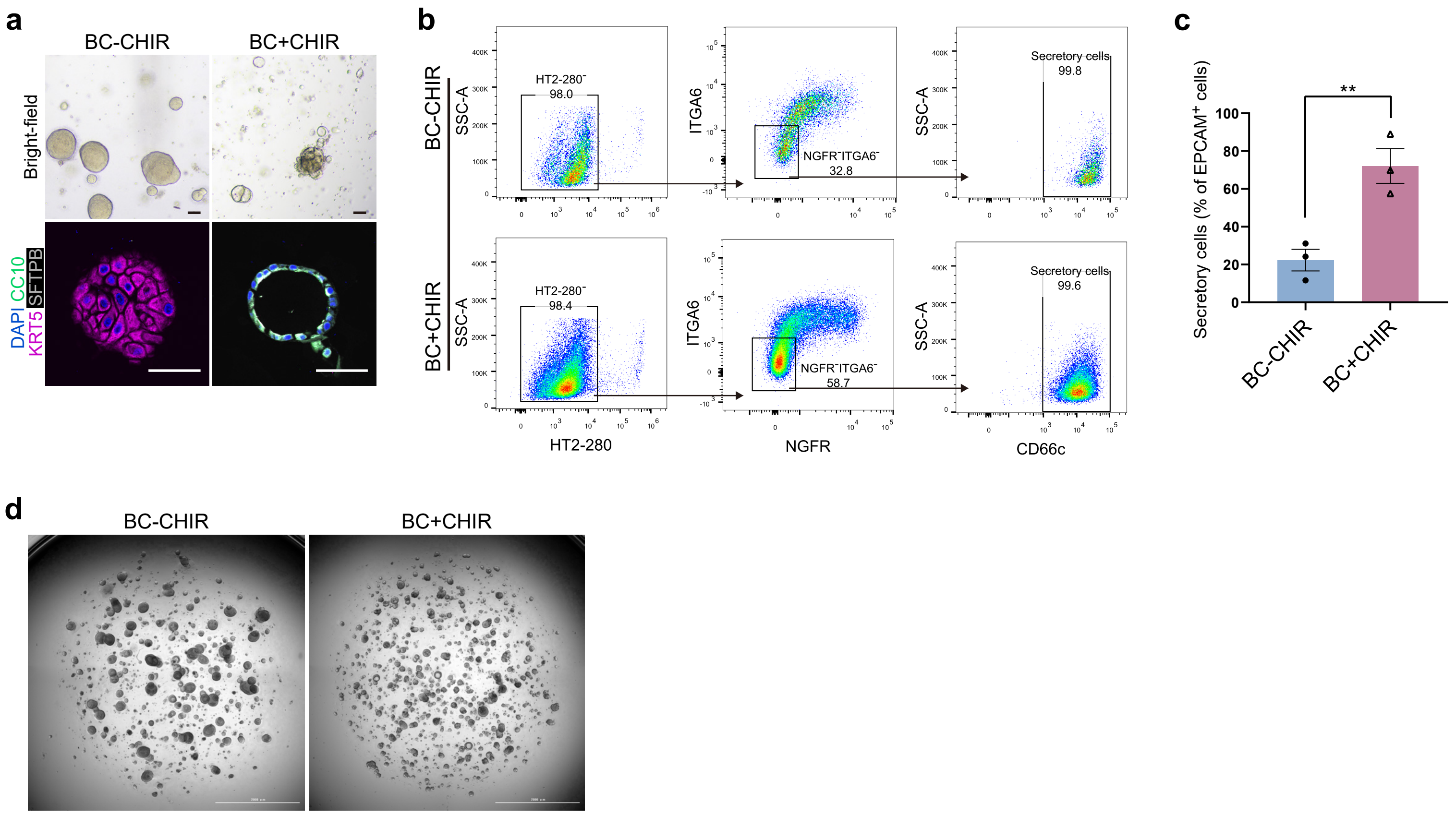

Figure S15. CHIR99021 promotes secretory features in CD66c⁻ basal cell (BC)–derived organoids.

(a) Representative bright-field (top) and immunofluorescence (bottom) images of CD66c⁻ BC–derived organoids cultured with or without CHIR99021 (CHIR) for 14 days. Immunostaining for KRT5 (magenta), CC10 (green), and SFTPB (gray) indicates acquisition of secretory features upon CHIR treatment. Scale bar, 50 μm.

(b) Representative flow cytometry plots of single-cell suspensions from CD66c⁻ BC–derived organoids cultured with or without CHIR, showing gating of EPCAM⁺ epithelial cells and identification of secretory cell populations.

(c) Quantification of secretory cell proportions among EPCAM⁺ epithelial cells from CD66c⁻ BC–derived organoids cultured without or with CHIR. Data are presented as mean ± SEM (n = 3; unpaired two-tailed Student's t-test). $^{**}p < 0.01$.

(d) Whole-well bright-field images of CD66c⁻ BC–derived organoids cultured with or without CHIR, showing no obvious difference in organoid-forming capacity. Scale bar, 2000 μm.

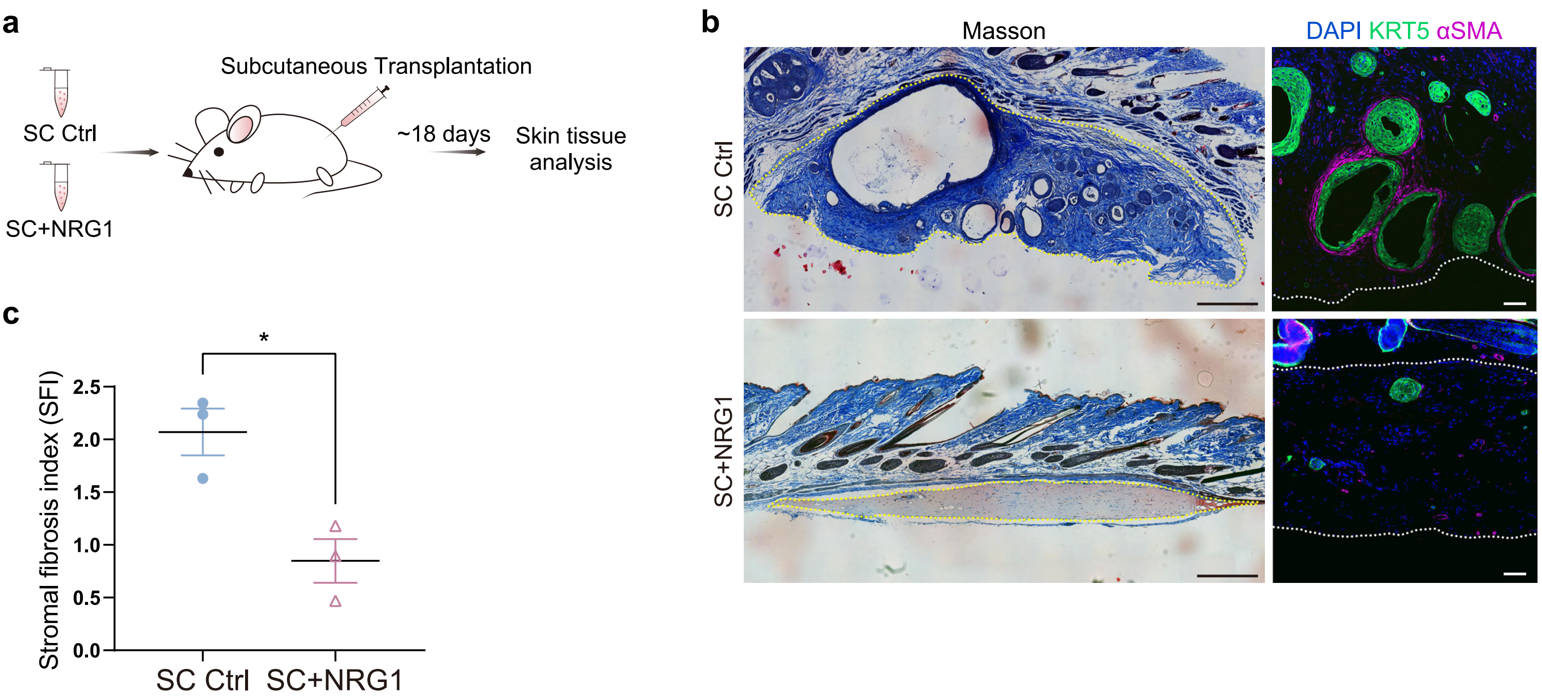

Figure S16. NRG1 treatment attenuates stromal fibrosis induced by secretory cell–derived organoid grafts in vivo.

(a) Subcutaneous transplantation of secretory cell–derived organoids (SC Ctrl vs. SC+NRG1) into NOD/SCID/IL2Rγ-null (NSG) mice. Grafts were analyzed at day 18 post-transplantation.

(b) Histological analysis of grafts. Masson's trichrome staining indicates reduced collagen deposition (blue) in NRG1-treated grafts. Immunofluorescence staining shows fewer αSMA^+ myofibroblasts (magenta) in the stromal regions surrounding $KRT5^+$ basal cells (green). Scale bars, 250 µm (Masson); 100 µm (IF).

(c) Stromal fibrosis index (SFI) quantification showing a significant reduction in NRG1-treated grafts compared with control grafts. Data are presented as mean ± SEM (n = 3; unpaired two-tailed Student's t-test). $^*p < 0.05$.

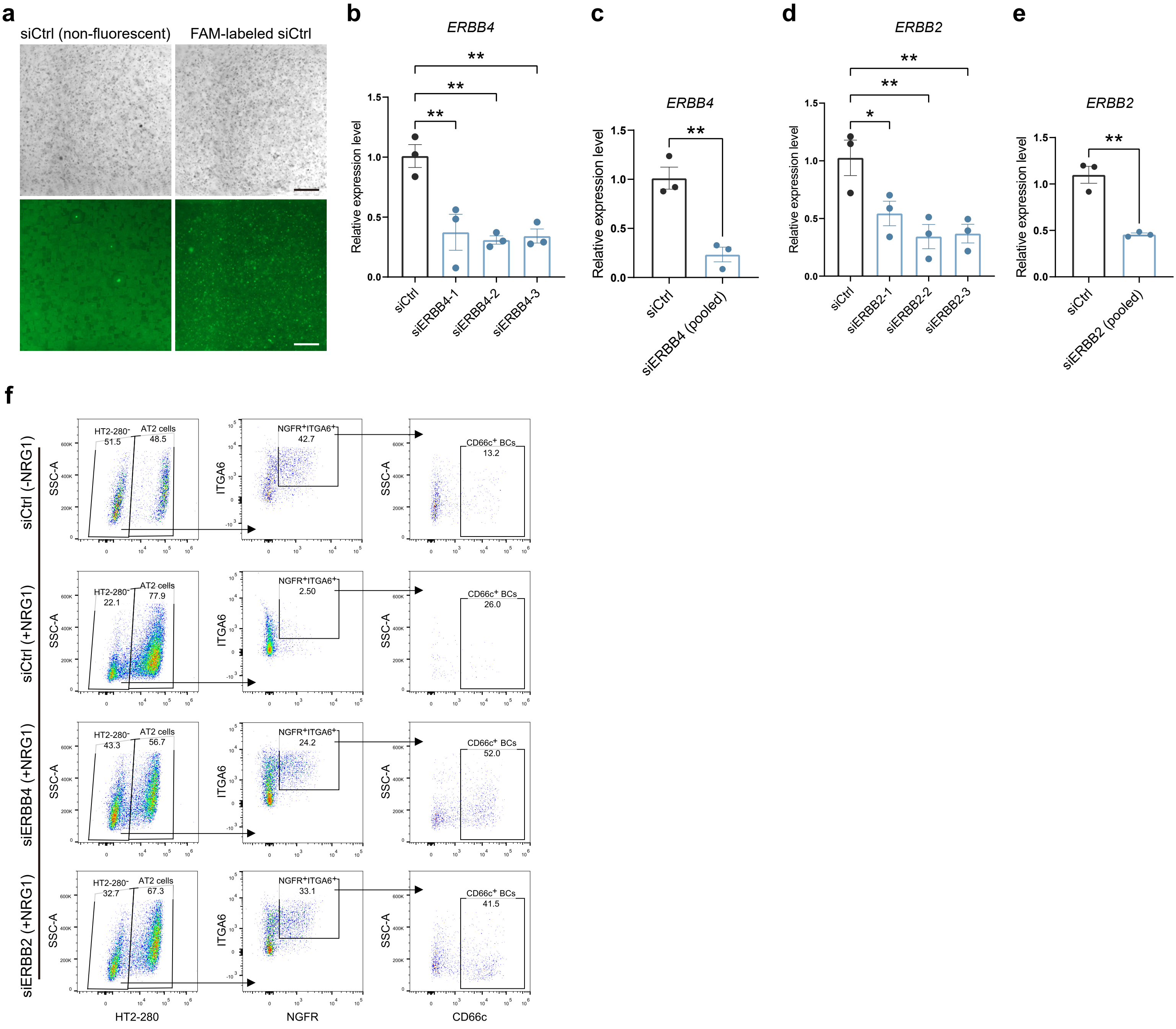

Figure S17. Validation of *ERBB4* and *ERBB2* knockdown efficiency and flow cytometric analysis of AT2 cell fate under NRG1 treatment.

(a) Representative bright-field and fluorescence images of organoids transfected with non-targeting control siRNA (siCtrl), shown without fluorescence or with FAM-labeled siCtrl to assess transfection efficiency.

(b–e) Validation of siRNA-mediated knockdown efficiency of *ERBB4* and *ERBB2* in human lung organoids. Relative mRNA expression levels of *ERBB4* (b, individual; c, pooled) and *ERBB2* (d, individual; e, pooled) were measured by RT–qPCR and normalized to siCtrl. Data are presented as mean ± SEM (n = 3; unpaired two-tailed Student's t-test). $^{*}p < 0.05$, $^{**}p < 0.01$.

(f) Representative flow cytometry plots showing the gating strategy and phenotypic analysis of AT2 cells ($HT2\text{-}280^{+}$ $NGFR^{-}$ $ITGA6^{-}$), indicating $CD66c^{+}$ basal cell proportions following siRNA transfection and NRG1 treatment.

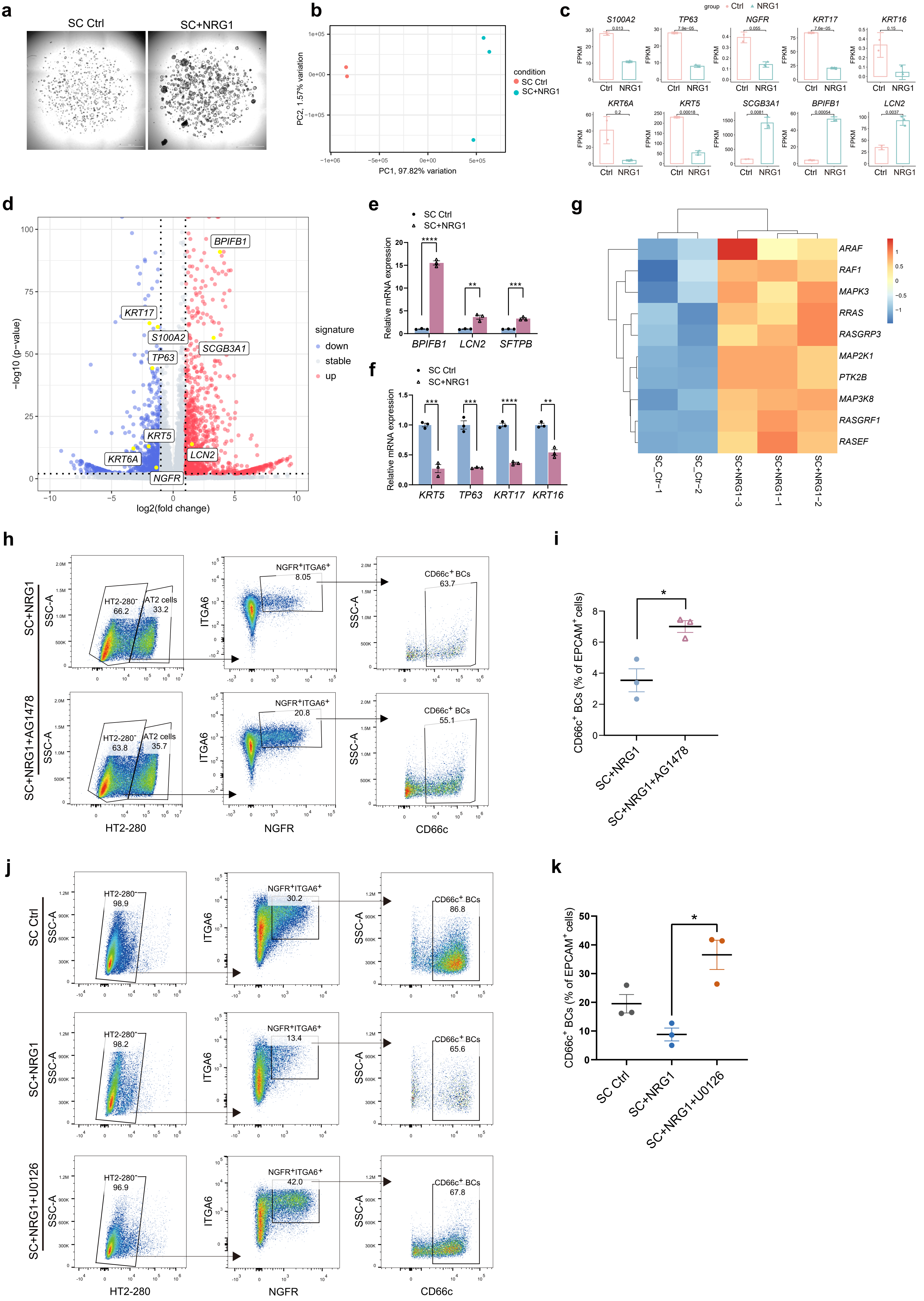
a
SC Ctrl
SC+NRG1
b
PC1, 97.82% variation
PC2, 1.57% variation
condition
SC Ctrl
SC+NRG1
c
group
Ctrl
NRG1
S100A2
TP63
NGFR
KRT17
KRT16
KRT6A
KRT5
SCGB3A1
BPIFB1
LCN2
FPKM
d
BPIFB1
KRT17
S100A2
SCGB3A1
TP63
KRT5
KRT6A
LCN2
NGFR
−log10 (p−value)
log2(fold change)
signature
down
stable
up
e
SC Ctrl
SC+NRG1
Relative mRNA expression
BPIFB1
LCN2
SFTPB
f
SC Ctrl
SC+NRG1
Relative mRNA expression
KRT5
TP63
KRT17
KRT16
g
ARAF
RAF1
MAPK3
RRAS
RASGRP3
MAP2K1
PTK2B
MAP3K8
RASGRF1
RASEF
SC_Ctr−1
SC_Ctr−2
SC+NRG1−3
SC+NRG1−1
SC+NRG1−2
h
SC+NRG1
SC+NRG1+AG1478
SSC-A
HT2-280⁻
66.2
AT2 cells
33.2
63.8
35.7
ITGA6
NGFR⁺ITGA6⁺
8.05
20.8
CD66c⁺ BCs
63.7
55.1
HT2-280
NGFR
CD66c
i
CD66c⁺ BCs (% of EPCAM⁺ cells)
SC+NRG1
SC+NRG1+AG1478
j
SC Ctrl
SC+NRG1
SC+NRG1+U0126
SSC-A
HT2-280⁻
98.9
98.2
96.9
ITGA6
NGFR⁺ITGA6⁺
30.2
13.4
42.0
CD66c⁺ BCs
86.8
65.6
67.8
HT2-280
NGFR
CD66c
k
CD66c⁺ BCs (% of EPCAM⁺ cells)
SC Ctrl
SC+NRG1
SC+NRG1+U0126

Figure S18. NRG1 suppresses basal cell and SPB-associated transcriptional programs in secretory cell–derived organoids through ERBB–MAPK–associated signaling.

(a) Bright-field whole-well images of secretory cell (SC)–derived organoids cultured with NRG1 or under control conditions. Scale bar, 2000 µm.

(b) Principal component analysis (PCA) showing clear segregation between NRG1-treated and control secretory cell–derived organoids.

(c) Box plots of FPKM values for representative basal cell (BC) markers (e.g. *S100A2*, *NGFR*, *KRT17*) and secretory-primed basal cell (SPB) markers (e.g. *KRT16*), as well as SC markers (e.g. *SCGB3A1*, *BPIFB1*, *LCN2*), in control versus NRG1-treated SC–derived organoids (n = 2-3). Data are shown as mean ± SD. Statistical significance was assessed using an unpaired two-tailed Student's t-test.

(d) Volcano plot of differentially expressed genes (DEGs) in NRG1-treated versus control SC–derived organoids, showing upregulation of secretory markers and downregulation of BC and SPB markers.

(e, f) RT–qPCR validation of selected DEGs in SC–derived organoids. (e) Secretory markers were increased, whereas (f) BC and SPB markers were reduced upon NRG1 treatment (n = 3).

(g) Heatmap showing MAPK pathway–related genes upregulated in SC–derived organoids following NRG1 treatment.

(h) Representative flow cytometry plots illustrating $CD66c^+$ BC analysis in SC–derived organoids treated with NRG1 alone or in combination with the ERBB4 inhibitor AG1478.

(i) Quantification of $CD66c^+$ basal cells among $EPCAM^+$ epithelial cells in SC–derived organoids treated with NRG1 alone or with AG1478 (n = 3).

(j) Representative flow cytometry plots of SC–derived organoids cultured under control conditions, treated with NRG1, or co-treated with NRG1 and the MEK inhibitor U0126.

(k) Quantification of $CD66c^+$ BCs among $EPCAM^+$ epithelial cells under the indicated conditions (n = 3).

Data are shown as mean ± SEM unless otherwise indicated. Statistical significance was determined by unpaired two-tailed Student's t-test for comparisons between two groups and by one-way ANOVA with appropriate multiple comparisons tests for analyses involving three groups. n represents independent biological replicates. *$p < 0.05$, **$p < 0.01$, ***$p < 0.001$, ****$p < 0.0001$.

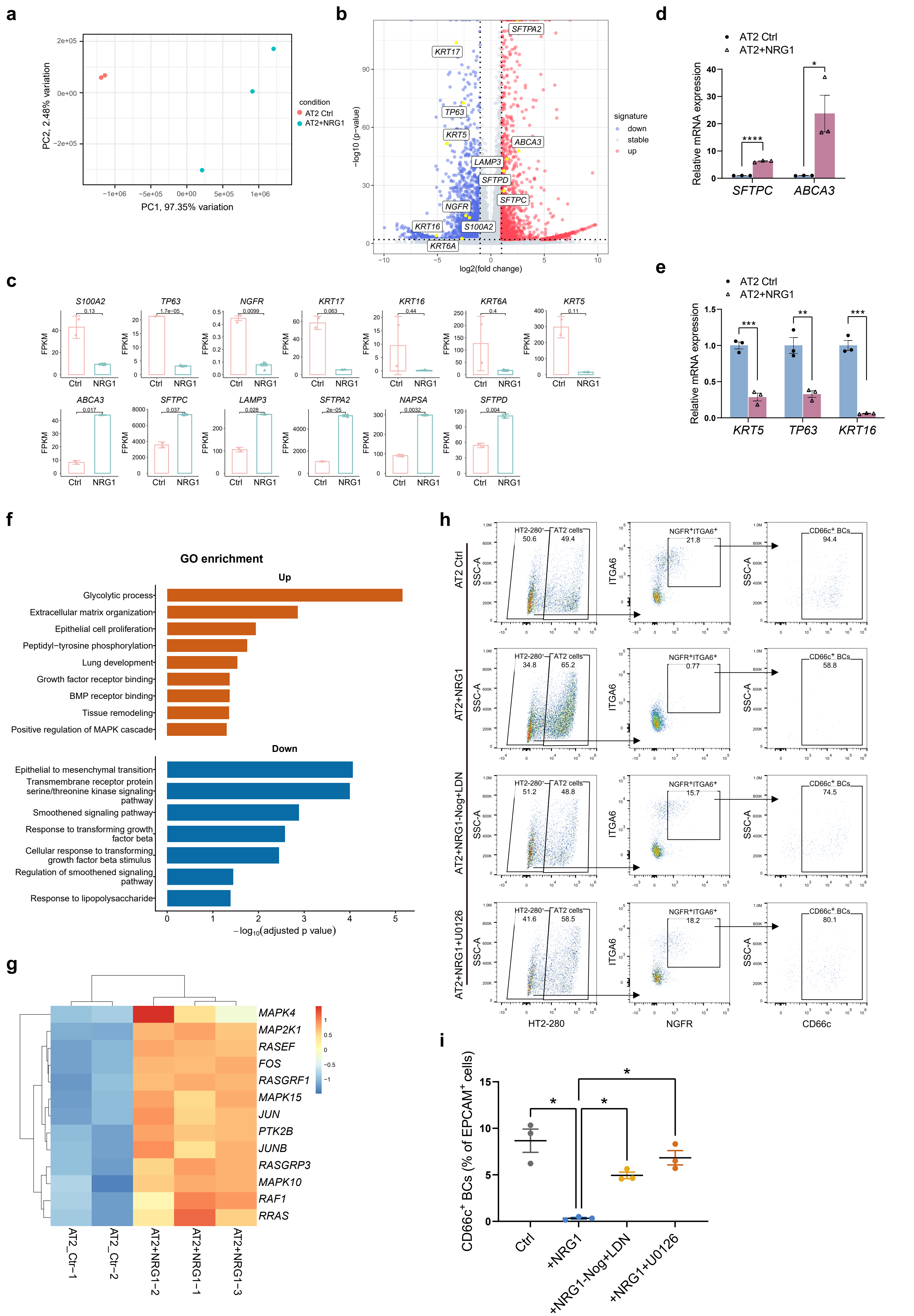

a
PC2, 2.48% variation
PC1, 97.35% variation
condition
AT2 Ctrl
AT2+NRG1
b
SFTPA2
KRT17
TP63
KRT5
ABCA3
LAMP3
SFTPD
SFTPC
NGFR
KRT16
S100A2
KRT6A
−log10 (p−value)
log2(fold change)
signature
down
stable
up
c
S100A2
TP63
NGFR
KRT17
KRT16
KRT6A
KRT5
ABCA3
SFTPC
LAMP3
SFTPA2
NAPSA
SFTPD
FPKM
Ctrl
NRG1
d
AT2 Ctrl
AT2+NRG1
Relative mRNA expression
SFTPC
ABCA3
e
AT2 Ctrl
AT2+NRG1
Relative mRNA expression
KRT5
TP63
KRT16
f
GO enrichment
Up
Glycolytic process
Extracellular matrix organization
Epithelial cell proliferation
Peptidyl−tyrosine phosphorylation
Lung development
Growth factor receptor binding
BMP receptor binding
Tissue remodeling
Positive regulation of MAPK cascade
Down
Epithelial to mesenchymal transition
Transmembrane receptor protein serine/threonine kinase signaling pathway
Smoothened signaling pathway
Response to transforming growth factor beta
Cellular response to transforming growth factor beta stimulus
Regulation of smoothened signaling pathway
Response to lipopolysaccharide
$-\log_{10}$(adjusted p value)
g
MAPK4
MAP2K1
RASEF
FOS
RASGRF1
MAPK15
JUN
PTK2B
JUNB
RASGRP3
MAPK10
RAF1
RRAS
AT2_Ctr-1
AT2_Ctr-2
AT2+NRG1-2
AT2+NRG1-1
AT2+NRG1-3
h
AT2 Ctrl
AT2+NRG1
AT2+NRG1-Nog+LDN
AT2+NRG1+U0126
HT2-280⁻
AT2 cells
NGFR⁺ITGA6⁺
CD66c⁺ BCs
SSC-A
ITGA6
HT2-280
NGFR
CD66c
i
CD66c⁺ BCs (% of EPCAM⁺ cells)
Ctrl
+NRG1
+NRG1-Nog+LDN
+NRG1+U0126

Figure S19. NRG1 preserves AT2-associated transcriptional programs and suppresses basal cell and SPB features in AT2-derived organoids via MAPK-associated signaling.

(a) PCA illustrating distinct separation between NRG1-treated and control AT2-derived organoids.

(b) Volcano plot of differentially expressed genes (DEGs) between AT2-derived organoids treated with NRG1 (AT2+NRG1) and controls. Upregulated genes include AT2 identity markers, whereas downregulated genes are enriched for basal cell–associated transcripts. Dashed lines indicate statistical thresholds.

(c) Box plots of FPKM values for representative basal cell (BC) markers (e.g. *S100A2, NGFR, KRT17*), secretory-primed basal cell (SPB) markers (e.g. *KRT16*), and representative AT2 markers (e.g. *ABCA3, SFTPC, LAMP3*) in control versus NRG1-treated AT2-derived organoids (n = 2–3). Data are shown as mean ± SD. Statistical significance was assessed using an unpaired two-tailed Student's t-test.

(d, e) RT–qPCR analysis of AT2-derived organoids cultured ± NRG1. (d) AT2 markers were upregulated, whereas (e) BC and SPB markers were downregulated upon NRG1 treatment (n = 3).

(f) Gene Ontology (GO) enrichment analysis of genes differentially expressed in AT2-derived organoids following NRG1 treatment.

(g) Heatmap showing MAPK pathway–related genes upregulated in AT2-derived organoids upon NRG1 treatment.

(h) Representative flow cytometry plots of basal cell analysis in AT2-derived organoids cultured under control conditions, treated with NRG1, or co-treated with NRG1 and LDN193189 in the absence of Noggin (Nog), or the MEK inhibitor U0126, as indicated.

(i) Quantification of $CD66c^{+}$ basal cells among $EPCAM^{+}$ epithelial cells under the indicated signaling perturbations (n = 3).

Data are shown as mean ± SEM unless otherwise indicated. Statistical significance was determined by unpaired two-tailed Student's t-test for comparisons between two groups and by one-way ANOVA with appropriate multiple comparisons tests for analyses involving three or more groups. n represents independent biological replicates. $*p < 0.05$, $**p < 0.01$, $***p < 0.001$.

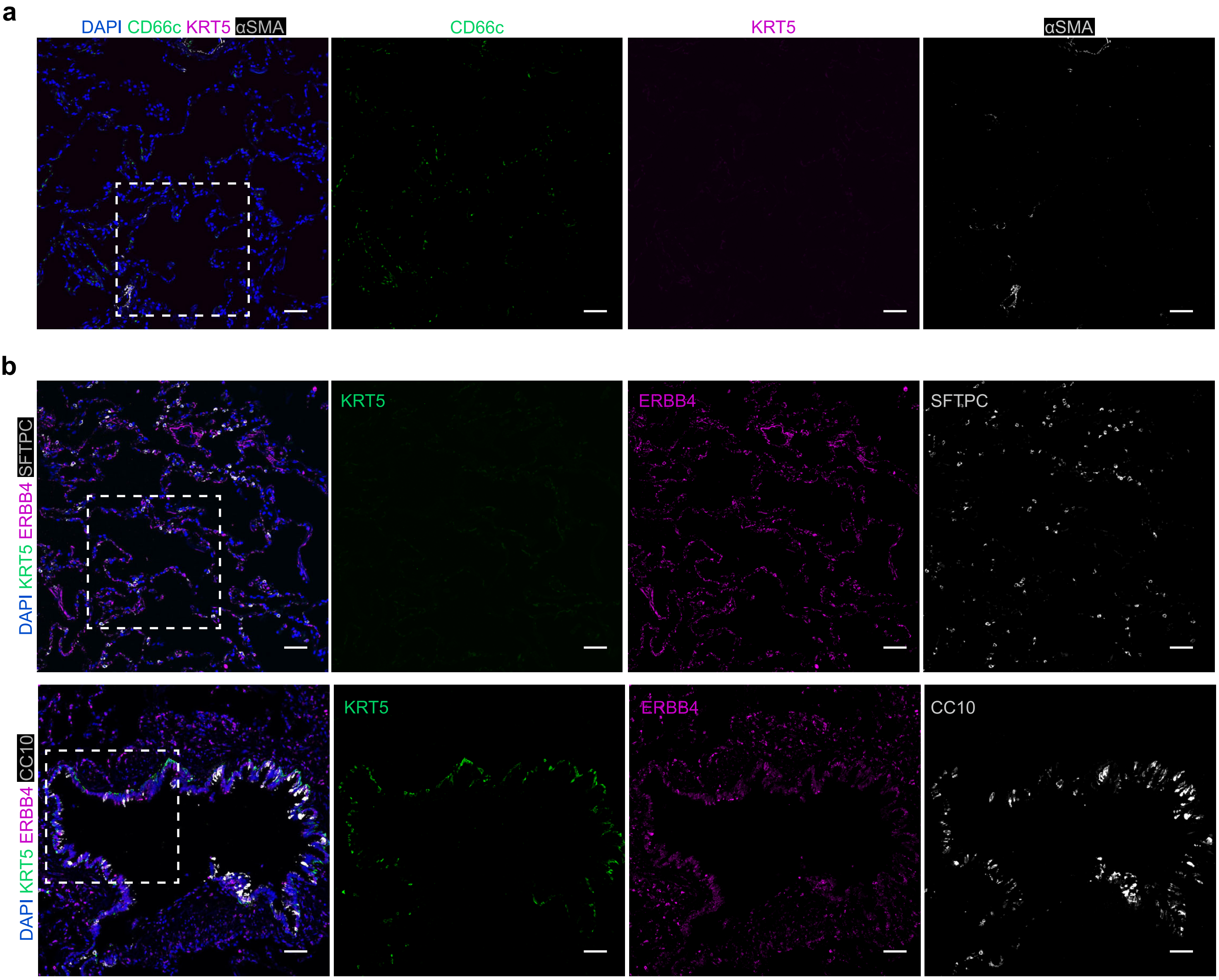

Figure S20. Low-magnification immunofluorescence images corresponding to Figure 8.
(a) Low-magnification view of healthy human lung tissue corresponding to the healthy control shown in Figure 8b. Dashed box indicates the region shown at higher magnification in Figure 8b. Scale bars, 50 μm.
(b) Low-magnification views of normal alveolar and airway regions corresponding to Figure 8d and Figure 8e, respectively. Dashed boxes indicate regions shown at higher magnification in the main figure. Scale bars, 50 μm.

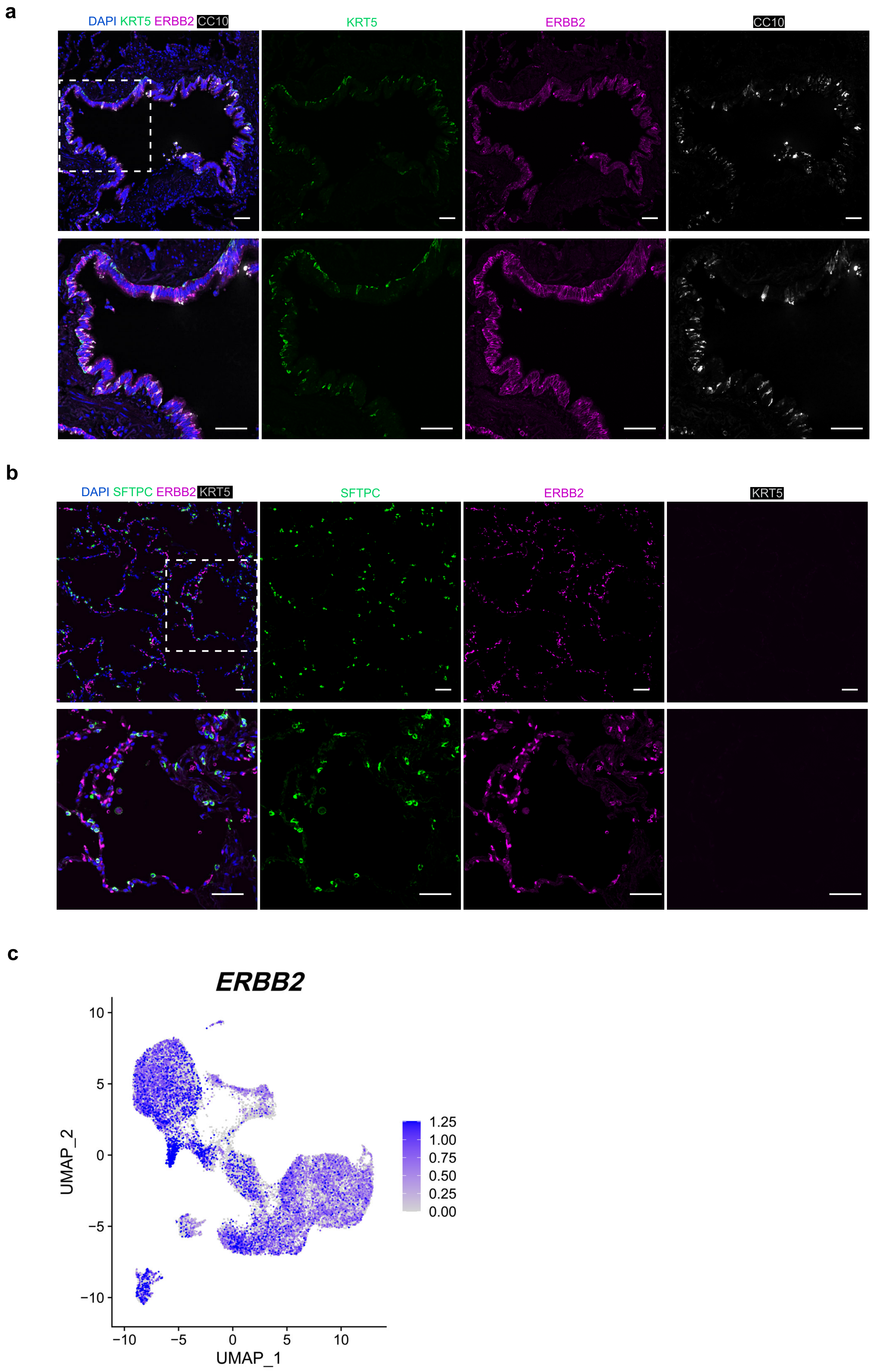

a
DAPI KRT5 ERBB2 CC10
KRT5
ERBB2
CC10
b
DAPI SFTPC ERBB2 KRT5
SFTPC
ERBB2
KRT5
c
ERBB2
10
5
0
−5
−10
UMAP_2
−10
−5
0
5
10
UMAP_1
1.25
1.00
0.75
0.50
0.25
0.00

Figure S21. Broad epithelial expression of ERBB2 in normal human lung tissue.

(a) Multiplex immunofluorescence staining of normal human airway epithelium showing ERBB2 expression across the airway lining. Upper panels show low-magnification views; lower panels show higher-magnification views of the boxed regions. Markers: ERBB2 (magenta), KRT5 (green), CC10 (gray), nuclei (DAPI, blue). Scale bars, 50 μm.

(b) Multiplex immunofluorescence staining of normal human alveolar regions demonstrating widespread ERBB2 expression in alveolar epithelium. Upper panels show low-magnification views; lower panels show higher-magnification views of the boxed regions. Markers: ERBB2 (magenta), SFTPC (green), KRT5 (gray), nuclei (DAPI, blue). Scale bars, 50 μm.

(c) Feature plot of *ERBB2* expression in the integrated single-cell RNA-seq dataset, including epithelial cells from uncultured distal lung tissue, day-14 DLOs, AT2-derived organoids, and normal or IPF lungs (Kathiriya et al., 2022).

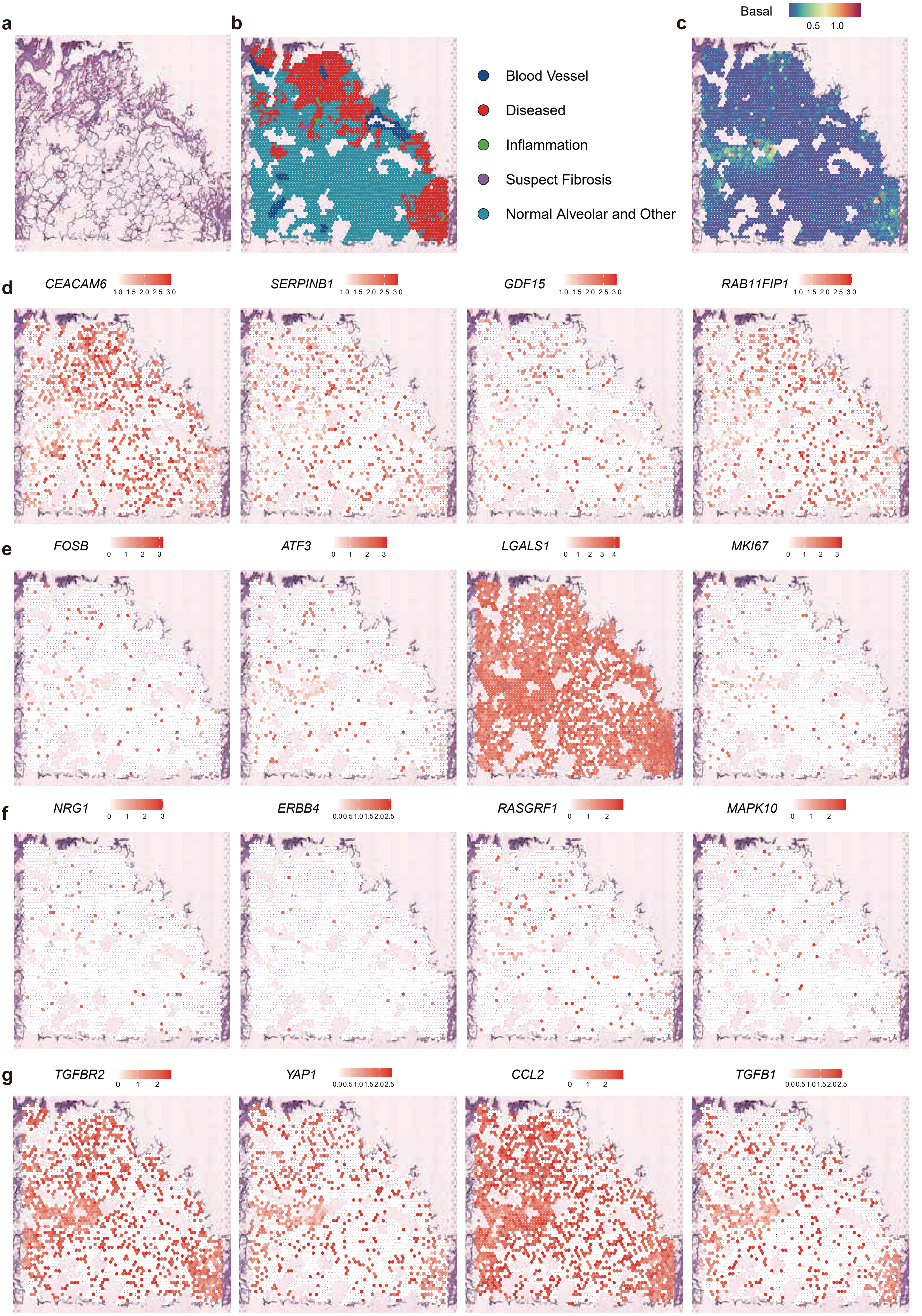
a
b
c
Basal
0.5
1.0
Blood Vessel
Diseased
Inflammation
Suspect Fibrosis
Normal Alveolar and Other
d
CEACAM6
1.0 1.5 2.0 2.5 3.0
SERPINB1
1.0 1.5 2.0 2.5 3.0
GDF15
1.0 1.5 2.0 2.5 3.0
RAB11FIP1
1.0 1.5 2.0 2.5 3.0
e
FOSB
0 1 2 3
ATF3
0 1 2 3
LGALS1
0 1 2 3 4
MKI67
0 1 2 3
f
NRG1
0 1 2 3
ERBB4
0.00.51.01.52.02.5
RASGRF1
0 1 2
MAPK10
0 1 2
g
TGFBR2
0 1 2
YAP1
0.00.51.01.52.02.5
CCL2
0 1 2
TGFB1
0.00.51.01.52.02.5

Figure S22. Spatial gene expression profiling of basal cell states and signaling pathways in IPF lung tissue.

(a) Hematoxylin and eosin (H&E)–stained section of lung tissue from a mildly fibrotic region of an IPF patient used for 10x Visium spatial transcriptomics analysis.

(b) Histopathological annotation of the same tissue section, highlighting diseased regions that were the primary focus of subsequent spatial analyses.

(c) Spatial distribution of inferred basal cells across the tissue section.

(d) Spatial expression maps of secretory-primed basal cell (SPB) marker genes (e.g. *CEACAM6, SERPINB1, GDF15,* and *RAB11FIP1*), showing enrichment predominantly within diseased and fibrotic regions.

(e) Spatial expression of marker genes associated with other basal cell subtypes, including activated basal (AB) markers (*FOSB, ATF3*), multipotent basal (MPB) marker (*LGALS1*), and proliferating basal (PB) marker (*MKI67*).

(f) Spatial expression maps of key components of the NRG1–ERBB4–MAPK signaling axis (*NRG1, ERBB4, RASGRF1,* and *MAPK10*), which display generally low expression levels and lack overt spatial enrichment within fibrotic regions.

(g) Spatial expression of genes from signaling pathways previously implicated in promoting epithelial plasticity and fibrotic progression, including TGFβ (*TGFB1, TGFBR2*), NF-κB (*CCL2*), and HIPPO (*YAP1*) pathways, showing enrichment within diseased areas.

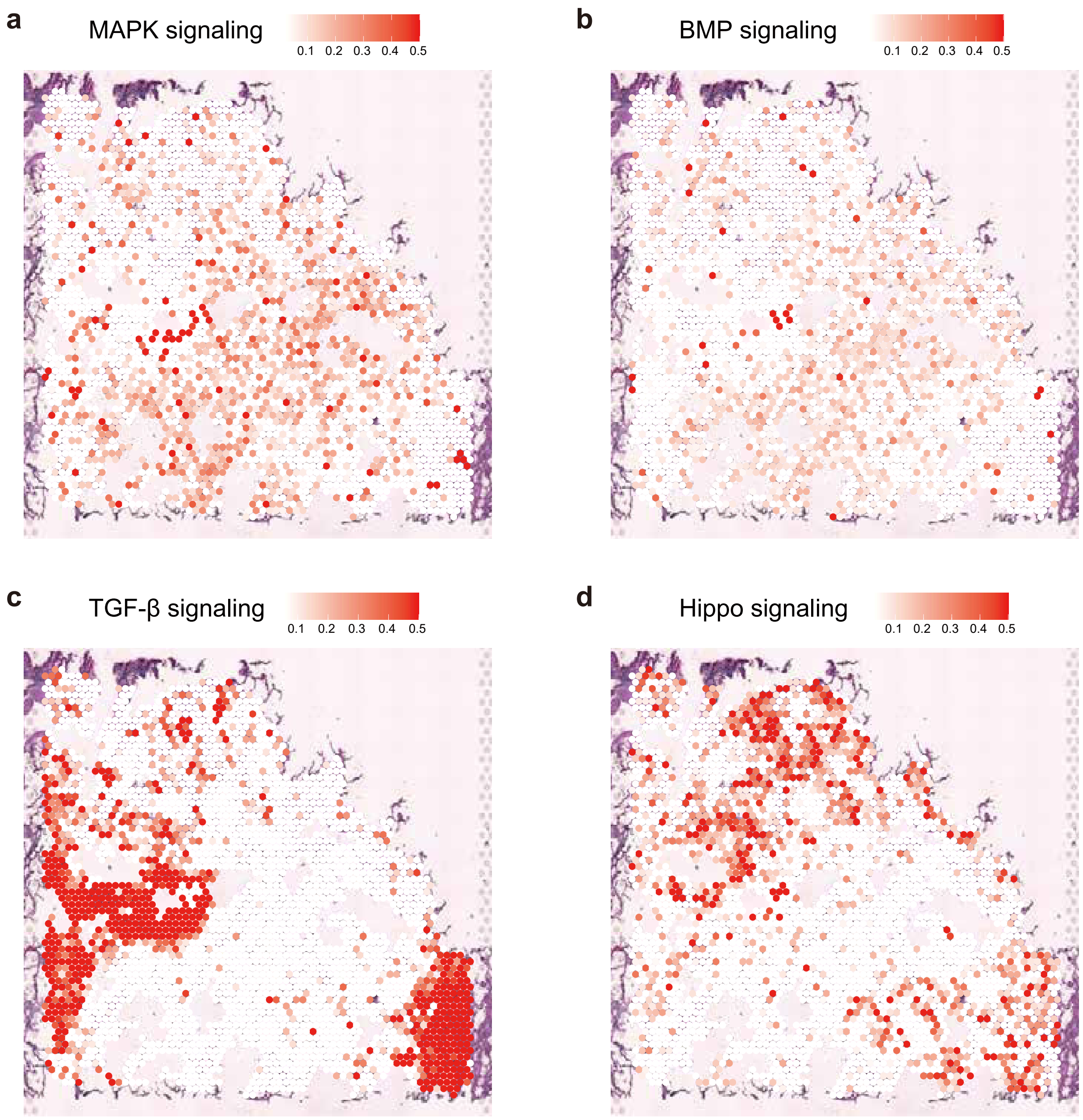

Figure S23. Spatial pathway activity analysis in IPF lung tissue.
(a–d) Spatial projection of pathway activity scores for MAPK (a), BMP (b), TGFβ (c), and Hippo (d) signaling onto 10x Visium sections from mildly fibrotic IPF lung tissue. Activity scores were calculated using MSigDB gene sets.

Supplementary References

1. Kathiriya, J. J., Wang, C., Zhou, M., et al. "Human alveolar type 2 epithelium transdifferentiates into metaplastic KRT5(+) basal cells." *Nat Cell Biol* 24, no. 1 (2022): 10-23. https://doi.org/10.1038/s41556-021-00809-4

2. Wang, S., Rao, W., Hoffman, A., et al. "Cloning a profibrotic stem cell variant in idiopathic pulmonary fibrosis." *Sci Transl Med* 15, no. 693 (2023): eabp9528. https://doi.org/10.1126/scitranslmed.abp9528

3. Franzén, L., Olsson Lindvall, M., Hühn, M., et al. "Mapping spatially resolved transcriptomes in human and mouse pulmonary fibrosis." *Nat Genet* 56, no. 8 (2024): 1725-36. https://doi.org/10.1038/s41588-024-01819-2
