# Supplementary material for: Activation of ERBB4 Pathway Inhibits Pathological Transdifferentiation of Lung Epithelial Progenitors into CD66c+ Basal Cells in Severe Lung Injury
Source: Adv Sci (Weinh). 2026 Apr 7:e19151. Online ahead of print. doi: 10.1002/advs.202519151 (PMC13334660; doi:10.1002/advs.202519151)
Supplement: Supplementary file 2 — Supporting File 2: advs75185‐sup‐0002‐TableS1‐S2.docx. [file ADVS-9999-e19151-s001.docx]

**Table S1. The culture medium of DLOs.**

|  | **Component** | **Final concentration** | **Treatment period** |
| --- | --- | --- | --- |
| **Base medium** | Advanced DMEM/F12 | | |
| **Compounds** | A83-01 | 0.5 uM | All Time |
|  | SB202190 | 0.5 uM | All Time |
|  | Y-27632 | 10 uM | Day 0 – 2 |
| **Recombinant human proteins** | EGF | 50 ng/mL | All Time |
|  | FGF10 | 100 ng/mL | All Time |
|  | FGF7 | 25 ng/mL | All Time |
|  | R-spondin | 500 ng/mL | All Time |
|  | Noggin | 100 ng/mL | All Time |
| **Supplements** | Nicotinamide | 5 mM | All Time |
|  | B-27 | 1x | All Time |
|  | Antibotic-Antimycotic | 100x | All Time |
|  | HEPES | 10 mM | All Time |
|  | GlutaMAX | 2 mM | All Time |
|  | N-Acetyl-L-Cysteine | 1.25 mM | All Time |

**Table S2. RT-qPCR primers used in this paper.**

| *Gene* | Direction | Sequence |
| --- | --- | --- |
| *HBEGF* | Forward  Reverse | ATCGTGGGGCTTCTCATGTTT  TTAGTCATGCCCAACTTCACTTT |
| *EREG* | Forward  Reverse | GTGATTCCATCATGTATCCCAGG  GCCATTCATGTCAGAGCTACACT |
| *NRG1* | Forward  Reverse | CGGTGTCCATGCCTTCCAT  GGGAGGCTGTTACTGTCATGC |
| *IL1RN* | Forward  Reverse | CATTGAGCCTCATGCTCTGTT  CGCTGTCTGAGCGGATGAA |
| *TGFA* | Forward  Reverse | AGGTCCGAAAACACTGTGAGT  AGCAAGCGGTTCTTCCCTTC |
| *LTBP1F* | Forward  Reverse | CAGCGTGCCTAAACTTTATCAGC  TCAGGAGGATGTTTCACATGGA |
| *CD44* | Forward  Reverse | CTGCCGCTTTGCAGGTGTA  CATTGTGGGCAAGGTGCTATT |
| *CAV1* | Forward  Reverse | ATGCCGTCAAAACTGTGTGTC  GCGACCCTAAACACCTCAAC |
| *CXCL8* | Forward  Reverse | ACTGAGAGTGATTGAGAGTGGAC  AACCCTCTGCACCCAGTTTTC |
| *MKI67* | Forward  Reverse | ACGCCTGGTTACTATCAAAAGG  CAGACCCATTTACTTGTGTTGGA |
| *COL1A1* | Forward  Reverse | GAGGGCCAAGACGAAGACATC  CAGATCACGTCATCGCACAAC |
| *TGFB1* | Forward  Reverse | GGCCAGATCCTGTCCAAGC  GTGGGTTTCCACCATTAGCAC |
| *IL11* | Forward  Reverse | CGAGCGGACCTACTGTCCTA  GCCCAGTCAAGTGTCAGGTG |
| *SMAD3* | Forward  Reverse | CCATCTCCTACTACGAGCTGAA  CACTGCTGCATTCCTGTTGAC |
| *TP63* | Forward  Reverse | GGACCAGCAGATTCAGAACGG  AGGACACGTCGAAACTGTGC |
| *KRT5* | Forward  Reverse | GCTGCCTACATGAACAAGGTGG  ATGGAGAGGACCACTGAGGTGT |
| *ABCA3* | Forward  Reverse | AGATGTAGCGGACGAGAGGA  GCTGCTCGTACACCTTGGAG |
| *LAMP3* | Forward  Reverse | AAGATGACCACTTTGGAAATGTG  GATGGCCCCAATCACAGGAA |
| *MUC1* | Forward  Reverse | TGCCGCCGAAAGAACTACG  TGGGGTACTCGCTCATAGGAT |
| *CEACAM6* | Forward  Reverse | TCAATGGGACGTTCCAGCAAT  CACTCCAATCGTGATGCCGA |
| *BPIFB1* | Forward  Reverse | CAGTGCCATGCGGGAAAAG  GCTGGAGGATGTTAGCTGTGA |
| *KDR* | Forward  Reverse | GTGATCGGAAATGACACTGGAG  CATGTTGGTCACTAACAGAAGCA |
| *CCND1* | Forward  Reverse | GCTGCGAAGTGGAAACCATC  CCTCCTTCTGCACACATTTGAA |
| *ACTA2* | Forward  Reverse | GTGTTGCCCCTGAAGAGCAT  GCTGGGACATTGAAAGTCTCA |
| *VIM* | Forward  Reverse | GACGCCATCAACACCGAGTT  CTTTGTCGTTGGTTAGCTGGT |
| *MMP1* | Forward  Reverse | AAAATTACACGCCAGATTTGCC  GGTGTGACATTACTCCAGAGTTG |
| *MMP3* | Forward  Reverse | AGTCTTCCAATCCTACTGTTGCT  TCCCCGTCACCTCCAATCC |
| *SCGB3A2* | Forward  Reverse | AAGCTGGTAACTATCTTCCTGCT  AGGGGCACTTTGTTGATGAGG |
| *TSPAN8* | Forward  Reverse | ACTTCTTGTTCTGGCTATGTGG  CACAGCAACGTAGGAGCTAGA |
| *SFTPB* | Forward  Reverse | GCAACGTCCTCCCCTTGAAG  AGTCAGTCTGGTTCTGGAAGTAG |
| *KRT16* | Forward  Reverse | GACCGGCGGAGATGTGAAC  CTGCTCGTACTGGTCACGC |
| *KRT17* | Forward  Reverse | GGTGGGTGGTGAGATCAATGT  CGCGGTTCAGTTCCTCTGTC |
| *NGFR* | Forward  Reverse | CCTACGGCTACTACCAGGATG  CACACGGTGTTCTGCTTGT |
| *KRT13* | Forward  Reverse | GACCGCCACCATTGAAAACAA  TCCAGGTCAGTCTTAGACAGAG |
| *SOX2* | Forward  Reverse | GCCGAGTGGAAACTTTTGTCG  GGCAGCGTGTACTTATCCTTCT |
| *NOTCH1* | Forward  Reverse | GAGGCGTGGCAGACTATGC  CTTGTACTCCGTCAGCGTGA |
| *NOTCH3* | Forward  Reverse | CGTGGCTTCTTTCTACTGTGC  CGTTCACCGGATTTGTGTCAC |
| *SFTPC* | Forward  Reverse | CACCTGAAACGCCTTCTTATCG  TTTCTGGCTCATGTGGAGACC |
| *SCGB1A1* | Forward  Reverse | TTCAGCGTGTCATCGAAACCC  ACAGTGAGCTTTGGGCTATTTTT |
| *ERBB4* | Forward  Reverse | GCAGATGCTACGGACCTTACG  GACACTGAGTAACACATGCTCC |
| *RASA4* | Forward  Reverse | CCCGCCAAGGACATCACTG  TCATCCATGACATAGAAAGCCAC |
